# Testing Anti-Biofilm Polymeric Surfaces: Where to Start?

**DOI:** 10.3390/ijms20153794

**Published:** 2019-08-03

**Authors:** Cristina Cattò, Francesca Cappitelli

**Affiliations:** Department of Food Environmental and Nutritional Sciences, Università degli Studi di Milano, via Celoria 2, 20133 Milano, Italy

**Keywords:** anti-biofilm surfaces, polymeric surfaces, biofilm methods, biofilm analysis, biofilm devices

## Abstract

Present day awareness of biofilm colonization on polymeric surfaces has prompted the scientific community to develop an ever-increasing number of new materials with anti-biofilm features. However, compared to the large amount of work put into discovering potent biofilm inhibitors, only a small number of papers deal with their validation, a critical step in the translation of research into practical applications. This is due to the lack of standardized testing methods and/or of well-controlled in vivo studies that show biofilm prevention on polymeric surfaces; furthermore, there has been little correlation with the reduced incidence of material deterioration. Here an overview of the most common methods for studying biofilms and for testing the anti-biofilm properties of new surfaces is provided.

## 1. Introduction

Polymeric materials, given their low cost, high specificity and adaptability [1], are currently used for a very broad range of applications ranging from structural materials to coatings, health care [2], packaging [3,4], communication [5], heritage [6,7], energy [8], transportation [9] and the agri-food industry [10]. Indeed, the very easy manipulation of molecular structure and chemical composition allows the production of innovative, advanced materials with specific chemical, biological, and physical features [1]. Polymer materials can be lightweight, hard, strong, and flexible, and can have peculiar thermal, electrical, and optical properties [1]. Consequently, in the last decade, material science has been experiencing an ever-growing active demand for innovative polymers of notable importance in present-day life.

Although polymeric materials play an invaluable role in providing solutions for a wide range of applications they are also easily colonized by biofilm, microorganisms that live in a self-organized, cooperative community attached to a substratum and covered by a self-produced matrix of extracellular polymeric substances (EPS) [11]. On the global scale, the impact of biofilm on present-day life is incalculable, with the spending of billions of dollars throughout the different sectors of the economy [12]. Biofilms are potentially able to contaminate all polymeric structural and infrastructural elements, systems, and devices, such as plumbing, medical implants, food processing facilities, and heating and air conditioning systems [13]. The result is a reduced industrial yield as well as the physical degradation of industrial systems such as pipe obstruction and corrosion [14]. In food-processing plants and drinking water networks, biofilm is a persistent source of microbial contamination that can affect the quality and safety of food products and water [15,16,17]. The worst biofilm reputation is most probably that of biofilm associated with medical implants, causing more than 60% of all microbial infection in humans [18,19]. Indeed, infection can give rise to complications, such as life-threatening systemic infections, contributing to post-operative morbidity, mortality, protracted hospitalization and re-operation rate, diagnostic tests and treatments increase, resulting in medical and financial burden [20,21]. It has been estimated that catheter-associated urinary tract infections cause approximately 40% of worldwide hospital-acquired infections, there being approximately 900,000 cases each year in the United States alone, at an annual cost ranging from 296 million to 2.3 billion dollars [22,23].

Strategies to alleviate the effects of biofilm formation on polymeric material have focused on cleaning and disinfection treatments aimed at killing microbial sessile cells already present on the surface. However, such treatments are not totally effective as biofilm microorganisms have features that provide successful conditions for microbial life, including enhanced resistance to antibiotic and biocide treatments [24,25]. Indeed, biofilm-associated resistance is due to several factors like the physiological state of the sessile cells themselves and their physical structure, as well as the presence of EPS that act as a barrier for such cells [26]. Furthermore, resistance towards many antibiotics has increased in several pathogenic microbial taxa, reducing the chances to treat effectively infections and increasing the risk of complications and fatal outcomes [27]. 

Consequently, in the past 20 years, studies in the field have addressed the development of preventive strategies, rather than approaches that kill microorganisms after their surface colonization. Indeed, the development of polymeric materials that can prevent microbial adhesion or weaken biofilm structure has emerged as a promising approach to overcome material-associated biofilm problems [28]. 

However, despite promising results, many experimental polymeric anti-biofilm surfaces reported in the literature have never been translated into real applications, nor have all newly created anti-biofilm surfaces undergone the critical step of validation of their anti-biofilm performance [29]. The in vivo testing of new anti-biofilm materials is an arduous task due to limited experimental control. It has been shown that in vivo assays can partially predict biofilm outcomes in humans, though there can be poor correlation with the clinical outcome [30,31]. Furthermore, and this is no less important, it is becoming more and more difficult to get approval for animal studies. Indeed, in most countries, the approval for animal experiments depends on convincing in vitro evidence of efficacy [29,32]. Tissue cultures have been used as a surrogate for in vivo biofilm studies, but the construction of a three-dimensional tissue culture is labour intensive and expensive. Moreover, experiments can only be conducted for short periods of time (i.e., in less than 24 hours) due to the cytotoxic effects on the cells, this cytotoxicity being due to both the biofilm itself and to the anti-biofilm surface, thus reducing the utility of these studies as biofilms generally take multiple days to reach maturity [20]. 

Therefore, attention here is paid to in vitro evaluation methods, which are a compromise between the reality of the in vivo ecosystem and the simplification of the system. However, a well-devised model and studies allow researchers to get relevant results [33]. Whereas there are several in vitro industrial standard tests to evaluate the antimicrobial (i.e., killing of microorganisms) efficacy of medical and non-medical products, there are no accepted standardized assays and validated methods to properly assess the activity of anti-biofilm material [34]. Indeed, current in vitro evaluation standard tests, especially tailored for specific action mechanisms that lead to cell death, are inadequate for today’s different advanced anti-biofilm surface designs. Note that the surface evaluation standard tests available today are mostly intended to test the ability of the material to abate microbial viability, without taking into consideration the differences in the mechanism of action [29]. Indeed, the tests attempt to evaluate biofilm inhibition and eradication without proper investigation of the variability in biofilm architecture and the complexity of its development [34]. This deficiency in methodology has adversely affected the translation of research into practical industrial and medical applications, and to regulatory agencies that assess the real-life usefulness of anti-biofilm surfaces.

Over the last couple of decades, a variety of simplified in vitro systems have been proposed to study biofilm formation [35]. Therefore, given the lack of standardized procedures to test anti-biofilm properties of materials, the only solution to test novel anti-biofilm surfaces for clinical purposes is to adapt the lab-scale devices and procedures presently used to obtain biofilms [36].

This review provides an overview of the most commonly used in vitro methods to study biofilm formation, and the recent findings available, and methods suitable for adaptation to test anti-biofilm polymeric surfaces. After presenting a brief description of the properties of the innovative anti-biofilm polymers provided to date by the scientific community, the authors propose currently available methods for evaluating the anti-biofilm activity of new anti-biofilm surfaces, and guidelines that would help readers choose the most appropriate test according to their objectives. Notably, the experimental procedure takes into account three steps: (i) identification of the relevant model microorganisms; (ii) selection of the experimental design for growing the biofilm on the new surface and (iii) determination and execution of the appropriate analysis.

## 2. Anti-Biofilm Polymeric Surfaces 

In the past, surface treatments aimed at killing microbial cells were proposed as valuable tools to counteract biofilm formation on polymeric materials. Indeed, the abatement of biofilm was obtained by spreading antimicrobial agents onto surfaces or by incorporating them into synthetic polymer-based products [37,38]. A number of antimicrobial-releasing surfaces have been proposed in the literature, and some of them have also reached the marketplace [38,39,40,41,42,43]. In these systems, the antimicrobial agent is actively eluted when the polymer surface comes into contact with an aqueous environment but, generally, control of the action is poor [44,45,46,47]. Indeed, smart responsive materials are designed to undergo an end-to-end chain reaction that releases the antimicrobial agent when activated by a stimulus at the terminal chain, i.e., the microbial presence [48,49].

In passive strategies, no antimicrobial agents are released into the surrounding environment. The physiochemical properties of a material surface, including composition, charge, hydrophobicity, roughness and porosity, are modified by: (i) applying to the surface, or mixing in the bulk, polymer anti-adhesive substances able to reduce the adhesion force between the microorganism and the solid surface [50,51,52,53,54]; and (ii) minimizing microbial attachment by providing the surface with a specific microstructure [55]. These approaches are relatively straightforward and economic. However, materials that preserve their resistance to microbial colonization are difficult to obtain by surface chemistry or surface structuring alone. Indeed, non-adhesive surfaces are often: (i) subjected to quick degradation or desorption over time [56,57]; and (ii) not always compatible with tissue cells, making them less suitable for biomaterial implants and devices requiring tissue integration [29]. 

In the last few years increased interest has been shown to metal nanoparticles. Nanoparticles have been successfully spread on, or incorporated into, a number of polymeric materials with versatile applications, including medical devices [58,59], marine industry paints [60] and food-contact surfaces [61]. Furthermore, nanotechnology has offered many opportunities for innovation. However, the use of nanomaterials has sometimes raised safety [62], environmental [63] and regulatory issues [64] that are still unresolved.

Nowadays, the literature reports strategies that exploit the potential of some compounds to interfere with microbial ability to develop biofilm by modalities perceived safe for human health. These approaches interfere with the following key steps that orchestrate the genesis of biofilm formation: (i) the surface sensing process to maintain the pioneering cells in a planktonic form, enabling easy removal of microorganisms before biofilm formation [65]; (ii) the disruption of biofilm physical integrity, by interfering with cell-to-cell communication or destroying the biofilm architecture by targeting the matrix [66]; and (iii) favoring biofilm dispersal by forcing the planktonic state [67,68]. In these approaches, microorganisms are still alive but deprived of their virulent properties. Thus, selection pressure decreases, limiting resistant-drug development, and potentially reinstating the efficacy of traditional antimicrobials [69]. Several natural and synthetic compounds, as well as matrix-targeting enzymes based on the previous biocide-free anti-biofilm mechanisms of action, have been coated or immobilized on polymeric surfaces, providing promising, eco-friendly, bio-inspired, anti-biofilm materials able to replace, or integrate with, presently dominating biocide-based approaches [69,70,71,72,73,74].

For example, Kim et al. [75] incorporated natural eugenol and clove oil into a biocompatible poly(D,L-lactide-coglycolide), markedly inhibiting biofilm formation and virulence of *Escherichia coli* O157:H7. Dell’Orto et al. [76] covalently grafted modified natural compounds, i.e., zosteric acid and salicylic acid, onto a low density polyethylene surface that was able to reduce *E. coli* adhesion, and thus biofilm formation, up to 73%. Sajeevan et al. [77] impregnated silicon catheter tubes with anacardic acids that efficiently inhibited *Staphylococcus aureus* colonization and biofilm formation on its surface both in vitro and in vivo. Spadoni-Andreani et al. [73] demonstrated that polypropylene surfaces coated with proteases weakened adhesion and increased the dispersion of *Candida albicans* biofilm cells and Cattò et al. [74] proved that the proteases α-chymotrypsin prevented *E. coli* biofilm formation on polyethylene materials

For further reading: recent progress in biofilm-resistant polymeric surfaces, provided by the material science community, has been extensively reviewed by Cattò et al. [36], Francolini et al. [18], Riga et al. [13] and Li et al. [49].

## 3. Microbial Choice

The selection of microorganisms to be included in experiments is a crucial choice. Keeping in mind the translation of the new material into real applications, the strain can be chosen ad hoc from among those existing in the natural environment where the material is to be placed. Indeed, as species vary a lot, depending on the environment, it is most important to choose and study the environment of interest.

**Figure 1:**
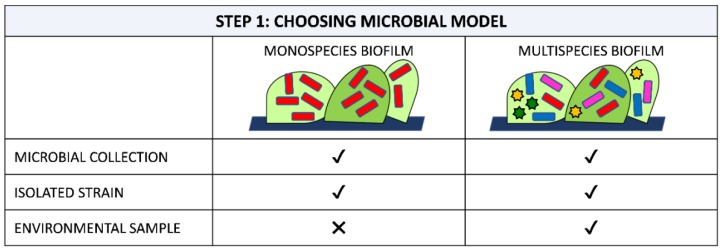
Scheme representing the first step in the experimental procedure for testing new anti-biofilm materials. The choice of the relevant model microorganisms includes the use of strains from microbial collections, strains isolated from the environment or complex environmental community samples used without any cultivation steps, in both mono- and multi-species biofilm models.

Choices include the use of strains in microbial collections [78,79,80], strains isolated from the environment [81,82] or complex environmental community samples used without any cultivation steps [53,83] (Figure 1). 

The simplest approach for studying a new material is to select a low-diversity model composed of a well-known, well-characterized, convenient and accessible laboratory strain. Such organisms should be representative of the living beings for which they are to serve as proxy. Some model microorganisms include *E. coli*, *Bacillus subtilis*, *Klebsiella pneumoniae*, *Pseudomonas* spp. and *Staphylococcus* spp. for bacteria, *Synechocystis* spp. for cyanobacteria, *C. albicans* and *Saccharomyces cerevisiea* for yeasts and *Fusarium oxysporum*, *Aspergillus* spp. and *Paenicillium* spp for filamentous fungi [78,84,85,86,87]. As these model microorganisms are frequently used, dedicated tools and resources for such organisms, e.g., databases, molecular kits, collections of techniques and methods, have been accumulated over the years, contributing to facilitate and standardize analysis [88,89]. 

In general, such monospecies systems have been proposed to achieve high reproducibility, short experimental timeframes and the application of widespread and well set up methodologies. They also provide several additional advantages such as low cost, easy set-up, and amenability to high throughput screens, addressing basic questions about biofilm development, physiology and architecture [90]. However, the results obtained with these systems cannot be completely translated into natural environments as the model strains were not isolated at the same time, nor at the place where the material is expected to work [91]. Indeed, as these lab strains are normally kept in laboratory stocks and have been cultured routinely, they may not exhibit the same phenotype as fresh isolates [92].

The approach based on isolated strains is better for obtaining a more representative view of biofilm behavior. Indeed, it is reported that, if repetitively cultured, microorganisms can evolve, resulting in a reduced capacity to form biofilm [93]. However, isolated strains are less known and distantly related to well-described model organisms from collections, resulting in a more complex application of conventional methods and assays. Another question is how to select the most relevant microorganisms among other isolates. At the moment, no consensus exists in the field, making results very difficult to compare between different works [92]. In the study of Rzhepishevska et al. [92], 19 strains of *P. aeruginosa* originating from hospitalized patients were studied and compared to the lab reference strain PAO1 and a rmlC lipopolysaccharide PAO1 mutant. The authors observed two sets of isolates, a group with high adhesion to a polymeric anti-biofilm coating and a group with low adhesion, including PAO1. Notably, they demonstrated that the properties of clinical isolates differed from that of the lab strain. Moreover, they highlighted the importance of choosing the right model strains to provide better predictability with respect to how materials inhibit biofilm formation.

Biofilm in a natural system consists of multiple microorganisms of different species, which often results in an enhanced survival capability, including improved tolerance against antimicrobial agents and virulence infections or increased stress tolerance, biomass production, metabolic cooperation, level signalling, compared to monocultures [94,95,96,97]. Most polymeric surfaces have been tested by examining monospecies biofilm formation, and the resulting information has been translated, by a variety of approaches, to biofilms composed of multiple species. However, the results published to date have revealed that biofilm features related to multispecies consortia cannot be predicted by studies performed with monospecies [98]. The number of papers focusing on multispecies biofilms on polymeric surfaces has increased in recent years to fulfill the need to study new materials in more appropriate experimental model systems, closer to the real environment of material application. Studies with multispecies biofilm have regarded all relevant areas, including materials for medical [70,99,100], industrial [101] and marine applications [81]. The experimented microbial communities have ranged from a relatively low diversity, two to four species, to complex systems consisting of hundred taxa. Notably, the types and number of interplays within multispecies biofilms grow exponentially with the increasing number of species. Therefore, many research groups have, to date, focused on biofilms comprised of two to four species. For these studies differential imaging of single species can be obtained by marking the different taxa with different fluorescent protein genes or probes. Cossu et al. [101] evaluated a novel anti-biofilm polymer formed using poly(vinyl alcohol-*co*-ethylene) with halamine suitable for food contact surfaces by exposing the new material to high loads of *Listeria innocua* and *E. coli O157:H7*. Nowatzy et al. [70] developed a new salicylic acid-releasing polyurethane acrylate polymer for anti-biofilm urological catheter coatings purpose and tested it by using a pool of *P. aeruginosa* and *E. coli*. Kommerein et al. [102] developed a highly reproducible in vitro four-species biofilm model consisting of the highly relevant oral bacterial species *Streptococcus oralis*, *Actinomyces naeslundii*, *Veillonella dispar* and *Porphyromonas gingivalis,* with a percentage distribution closely reflected the situation in early native plaques. These systems are typically highly reproducible and fast-growing. However, they may not completely replicate communities in their native environments in terms of number and relative proportion of species, phenotypes (laboratory stock instead of wild type) or culture conditions. 

In contrast, natural multispecies biofilm systems stem from isolated organisms or organisms directly taken from the environment of interest. For instance, Le Norcy et al. [81] reported the assessment of a varnish based on a biodegradable polymer, poly(ε-caprolactone-co-δ-valerolactone) incorporating a hemibastadin derivative, against a mixture of *Paracoccus* sp., *Pseudoalteromonas* sp. and *Bacillus* sp., isolated from the Gulf of Morbihan in France. After isolation, the strains were cultivated and used in different proportions to assess the anti-biofilm performance of the new varnish. In a more complicated system, Zhang et al. [83] synthesized an anti-biofilm dental composite by combining 2-methacryloyloxyethyl phosphorylcholine with quaternary ammonium dimethylaminohexadecyl methacrylate, and evaluated the materials against an oral biofilm plaque from the saliva of 10 donors. These natural biofilm systems better mimic the species composition of the environment than do engineered model systems. However, these methods use a limited subset of isolated species, which in most of cases corresponds to the most highly abundant species within the community, giving less importance to the less abundant taxa despite their playing a key role within the community [98]. Furthermore, strains from an environment of interest can be isolated using culture-based approaches, with the risk of excluding important non-cultivable species. Another problem encountered in these systems is reproducibility, as a complex experimental design is commonly detrimental to reproducibility [103]. 

In light of these considerations, system selection is a trade-off between the use of a mono species system that follows a simple, easy, highly reproducible, time- and cost-effective approach, but less reflective of the real environment, and a more laborious, complex and time intensive method that closely mimics reality [98]. The ideal scenario would be to use the new material in the real environment to more easily find out the succession, organization, and role of each specific species [53]. However, in situ observations are often not feasible due to the high complexity of the system. The relevant system for testing new anti-biofilm surfaces might be one that mimics the real environment in a simplified design without losing the relevant interplays and dependencies of natural biofilm.

## 4. In Vitro Methods to Culture Biofilm on Anti-Biofilm Polymeric Surfaces

**Figure 2:**
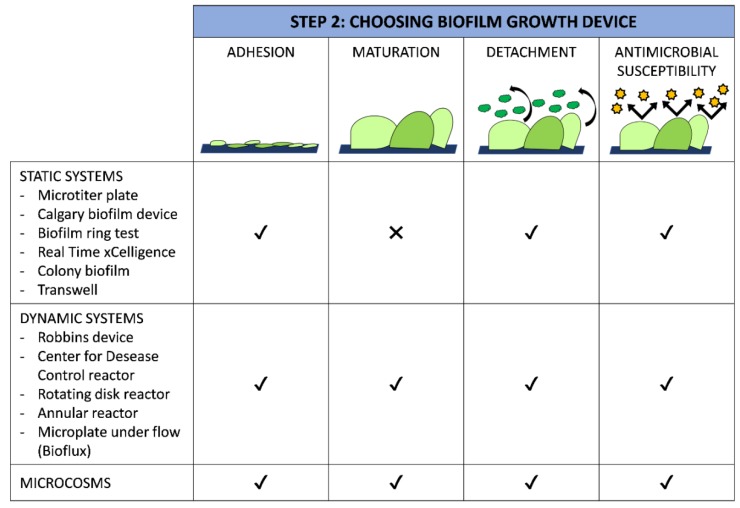
Scheme representing the second step in the experimental procedure for testing new anti-biofilm materials. The choice of the biofilm growth device includes the use of static, dynamic or microcosms systems, according to the step of biofilm formation to be analysed.

Biofilm culturing techniques should be chosen according to the study goals, simulating the environment and according to the availability of resources and skills (Figure 2). No less important is the fact that different devices present advantages and disadvantages, which must be considered before using them.

Technical information of the most popular laboratory devices is discussed below, and their strengths and limitations are shown in Table 1.

### 4.1. Static Methods

Static methods are particularly meaningful for examining early events in biofilm development, detecting cell attachment within a 15–60 minute time frame [109]. Such assays can be used to identify the impact of innovative surfaces in modulating the transition from a planktonic to a sessile mode of existence. Indeed, they effectively identify factors that initiate biofilm formation, like flagella, pili, adhesions, enzymes associated with cyclic-di-GMP binding and metabolism [110].

Static systems are widely used because they are simple to use, high producible, and controllable, and show limited contamination, and cost-effective properties. These easy-to-use and cost-effective assays make them amenable for large-scale high-throughput screening purposes, like genetic screens, and are useful for studying multiple strains under various growth conditions [110].

However, these closed models do not allow for the flow of media, product or waste materials into or out of the system, with the consequence that the experimental conditions change progressively because of nutrient depletion and the metabolic products build-up [32]. Thus, the biofilm growth rate is rapid at the beginning when there is an ample amount of nutrients [33]. However, in the natural environment this is uncommon [44], which means that the physiological and biological features of experimentally induced biofilm are not comparable with natural biofilm, precluding a full evaluation of the effect of new materials on biofilm development and dispersion.

#### 4.1.1. Microtiter Well Plate

The simplest experimental system to study microbial adhesion on a surface relies on the use of microtiter well plates, growing biofilm in static conditions. The surface of microtiter plates can be modified or, alternatively, a novel material can be inserted into the wells. The general protocol of microtiter plates permits the inoculation of microtiter wells with a cell suspension over a desired time period, while allowing cells to sediment on a substratum. After a specific time period in which microbial adhesion occurs, the wells are emptied or the material previously inserted in the well is carefully removed. Surfaces are then washed to remove planktonic cells and the number of adhering viable microorganisms is assessed. The removal of the liquid phase above the substratum must be done carefully to avoid the inadvertent removal of adhering cells [109].

For instance, Lin et al. [111] directly coated the surface wells of a 96-well polystyrene microtiter plate with 1,2,3,4,6-penta-*O*-galloyl-β-d-glucopyranose, an active plant ingredient commonly used in Chinese medicine, to test the coating efficacy against *S. aureus* biofilm. Alternatively, Swartjes et al. [112] demonstrated that polymethylmethacrylate (PMMA), a commonly employed biomaterial, coated with DNase I significantly reduced adhesion of *S. aureus* (95%) and *P. aeruginosa* (99%), and inhibited biofilm development for up to 14 h in static conditions. Salta et al. [113] coated glass discs with a paint composed by PMMA and a natural compound derived from walnut trees, and tested the new material against marine biofilm formation in a 24-well plate.

#### 4.1.2. Calgary Biofilm Device

In this system biofilm development is studied at the coverlid, composed of pegs that fit into the wells of a microtiter plate containing nutrients and microorganisms [114]. The advantage of this approach is that the biofilm is not the result of the cell sedimentation but the adhesion of cells to the pegs. This reduces the interference of those planktonic cells that remain at the bottom of the microtiter plate wells after the washing step [115]. In order to investigate biofilm formation on specific abiotic supports, coverlid can be customized with materials with specific anti-biofilm features or pegs coated with anti-biofilm molecules. Harrison et al. [116] used the Calgary biofilm device to demonstrate that *C. tropicalis* biofilm increased on polystyrene pegs coated with L-lysine [116]. Nowatzki et al. [70] coated the peg of the Calgary biofilm device with a polyurethane acrylate polymer partly composed by salicyl acrylate, which degrades in aqueous conditions, releasing salicylic acid.

However, Calgary device assays also require the washing off of non-adherent microorganisms and cell recovery, resulting typically between 5% and 90% of the entire community [35].

#### 4.1.3. The Biofilm Ring Test (BRT)

The principle of this assay is based on the capacity of microorganisms to immobilize magnetic microbeads when developing a biofilm at the well surface of a microtiter plate. Once a magnetic field is applied, beads are free to move and gather in the center of the well bottom. Once a biofilm has developed, the center of the well cannot accumulate the beads as the biomass prevents it, and this outcome can be measured using a specific plate reader [117,118]. After paramagnetic microbeads are added to the microbial suspension, they are loaded into the wells of a microtiter plate. The microtiter plate is then incubated, and direct measurements can be carried out [35]. The measurement of this super-paramagnetic microbead immobilization by adherent cells at different time points can be used to evaluate the kinetics of biofilm development. Indeed, the microtiter well can be modified by anti-biofilm coatings as well as customized with a desired material, and the anti-biofilm properties of the new surface can be analyzed.

This assay requires neither washing nor staining, thus avoiding procedures that can generate some significant bias in the outcomes. Moreover, it is easy to handle, and more importantly, the results are achieved in a few hours. Additionally, it could be well-suited to study the synergism of the new material with a biocide.

Unfortunately, BRT can only measure thick biofilms that develop at the bottom of the microtiter well [34]. Furthermore, it is only useful when the new anti-biofilm materials display good magnetic properties. Additionally, the procedure does not provide, in a single experiment, direct information about biofilm production on the different anti-biofilm surfaces as the strength of the magnetic field is influenced by both the type of materials and/or the thickness of the coating [119]. Indeed, there can be differences in the magnetic field strength between the new anti-biofilm material and the relative control, leading to analytical bias in the results. To date, to the best of the authors’ knowledge, no studies have investigated the performance of new anti-biofilm surfaces using BRT.

#### 4.1.4. Real-Time xCelligence

The xCelligence system uses specially designed microtiter plates with inter-digitized gold microelectrodes to non-invasively study the status of adherent cells, using electrical impedance as the readout [120]. Indeed, biofilm growth impedes the flow of an alternating microampere electric current between the electrodes. This impedance is measured automatically at intervals defined by the user, and allowes a highly sensitive readout of cell amount, cell size/shape, and cell–surface adhesion strength [121].

Because each xCelligence well can collect thousands of data, each individual well gives a complete time course for biofilm deposition or dissipation, significantly reducing the number of wells needed and the total workload. Additionally, the manual collection of endpoint data is eliminated, and multiple drugs/conditions can be analyzed simultaneously in a single plate, greatly improving throughput. Recently, the system was used to measure microbial biofilms and to study the effect of antimicrobial treatments on biofilm growth [122].

This system is non-invasive, label-free, fast, and reproducible [123]. The microtiter well can be modified by anti-biofilm coatings, as well as customized with a desired material, and the anti-biofilm properties of the new surface can be analyzed. On the other hand, the manufacture of microtiters with specific materials and correct gold biosensor insertion in each well is technically challenging. Furthermore, like BRT, xCelligence technology is not really suitable for the comparison of microbial adhesion on different surfaces as electrical impedance is influenced by both the type of material and the thickness of the coating [124]. Moreover, the xCelligence machine is costly and requires specialized detection equipment, making its availability not always affordable by the individual researcher [34].

#### 4.1.5. Transwell Device

This consists of a plastic transwell insert support inlaid in a well plate with cultural medium, providing semi-batch working conditions and two separate chambers [125,126,127]. A semipermeable membrane-like thin film of the new anti-biofilm material is placed in the support, and the cell broth in the well of the culture plate. The microbial cells are not allowed to move across the two compartments, and the anti-biofilm surface to which the cells attach themselves is the only avenue for meeting nutritional needs and removing waste. The supports are periodically relocated to fresh plates, thus providing the biofilm with a semi-constant fresh supply of nutrients. After one to three incubation days, the transwell inserts are removed and the biofilm formation on the surface support is analyzed. Transwell systems have a fine control of the experimental conditions in the two chambers, a semi-constant nutrient support and the possibility to collect the metabolites by biofilm culture in the basolateral media of the plate well compartment, without the risk of contamination by planktonic cells [128]. Transwell devices are now commonly used to form biofilm under various physiological conditions [125,126,129].

#### 4.1.6. Colony Biofilm

This technique analyzes biofilm formation at the air–surface interface, without submerging the biofilm in liquid [130]. Biofilms are cultivated directly on the anti-biofilm material that must be customized in the form of a semipermeable membrane-like thin film (e.g., wound gauzes, polycarbonate membranes), which are placed on the surface of agar Petri plates. At regular intervals, the film materials are transferred to a fresh medium, giving the biofilm a semi-continuous supply of fresh nutrients. The thick membrane is a convenient substratum that is easily maneuverable, allowing the surface-grown biofilm handling from one medium source to another. These colony biofilms can grow in a short period of time, are easy to grow and require inexpensive laboratory materials.

However, since there is no continuous flow of medium, the microorganisms are not forced to adhere to the surface, and, since there is no wash-out, planktonic cells can interfere with the biofilm assay [125]. Additionally, the stable and spatially restricted nature of this system makes cell number counts more easily attributable to cell lysis rather than detachment [109]. Furthermore, microbial taxa that differ in surface motility will spread across the material at different rates. Other disadvantages include difficulty in handling membranes when colony biomass enlarges [130].

Colony biofilm assays allowed Tran et al. [131] to successfully examine the effectiveness of cellulose discs coated with organoselenium in inhibiting *P. aeruginosa* and *S. aureus* in biofilm-related wound infections. Epstein et al. [57] used colony biofilm to show that a slippery liquid-infused porous surfaces prevented 99.6% of *P. aeruginosa* biofilm attachment over a 7-day period, as well as *S. aureus* and (97.2%) and *E. coli* (96.0%).

### 4.2. Dynamic Methods

Dynamic systems resemble in vivo conditions better than static ones, due to the control of nutrient delivery and flow, simulating, for instance, the flow forces in urinary and cardiovascular catheters, industrial installations and water pipelines [132].

Dynamic methods usually begin with an adhesion step performed in a low nutrient suspension, as the presence of nutrient-rich suspensions reduces the need for planktonic cells to adhere to a substratum. In subsequent steps, the continuous pumping of nutrients into the reactor leads to stress conditions that promote biofilm growth on the potential anti-biofilm surfaces [133]. Nutrient availability is accurately controlled in such a way as to promote biofilm development without producing artifacts, e.g., complete nutrient depletion that could lead to inhibition of biofilm growth.

Dynamic systems are highly suitable to assess contact-killing materials as, after the adhesion step, the suspension of non-adhered cells is flushed out of the bioreactor, allowing only adhered cells to develop into mature biofilm. On the contrary, they are less suitable to assess antimicrobial-releasing materials as the antimicrobials released are washed out of the system.

Flow systems have the advantage of simultaneously testing different surface materials, analysing samples in a non-invasive manner and with standardized protocols [134]. Additionally, bioreactors allow the sampling of materials aseptically at different time points during biofilm development, without compromising the entire experiment. Furthermore, they are convenient for studies where a large biofilm biomass amount is desirable and for those studies involving microsensor monitoring [135]. Notably, the dry weight of biofilm grown in dynamic systems is often reported to be around 100–175 g dry weight kg^−1^ biofilm, with values comparable to the solid content of centrifuged biomass from suspended cultures [136].

While each of these methods is a useful tool for studying biofilm under controlled conditions, they all need specialized apparata, are technically challenging, and are not suitable for rapid high throughput assays. Another weakness of these systems is that only a single strain can be analysed per experiment. Moreover, biofilm models are difficult to compare due to the differences in biofilm development times, growth media, and the microbial taxa employed.

#### 4.2.1. Robbins (RO) Reactor

This reactor consists of a plug where coupons (biofilm growth surfaces) can be mounted. The plug has two ports for fluid entry and exit. Coupons of different materials can be customized and simultaneously mounted on the device.

The Robbins (RO) reactor is not designed to allow direct observation of biofilm growth. Thus, the coupons must be dislodged for further studies. Additionally, the flow dynamics inside the device need to be accurately adjusted to make sure that the flow is constant along the plug [35]. Indeed, a trend towards higher numbers adhering to the coupons at the in-flow end of the RO reactor than at the outflow end was recorded, likely reflecting reduction of adherent bacteria in the interacting stream [137].

The original RO reactor was employed to evaluate biofilm development under different fluid velocities in a simulated drinking water facility [138]. Linton et al. [137] used a modified RO reactor to compare the adhesion of *S. epidermidis* to glass, siliconized glass, plasma-conditioned glass, titanium, stainless steel and Teflon in a medical environment. Oosterhof et al. [139] tested the fungal and bacterial biofilm responses on tracheoesophageal shunt prostheses to quaternary ammonium silane coatings. Ramage et al. [140] employed an RO reactor to grow *C. albicans* on PMMA. Ginige et al. [141] installed a modified RO reactor in a full-scale water distribution system to investigate biofouling on a high-density polyethylene surface.

#### 4.2.2. Center for Disease Control (CDC) Reactor.

This reactor is a vessel with 8 rods hosting removable coupons for a total of 24 samples. The coupons can be tailor-made from various materials that can be examined simultaneously in a single assay. Moreover, coupons can be removed or exchanged during the experiment allowing time-course studies.

The rotation of the baffled stir bar leads the coupons to undergo a consistent high shear and, as they are placed at the same radial distance, they are subjected to the same shear stress. Nutrients are continuously pumped into and out of the reactor at the same rate [133]. These stress conditions promote biofilm formation on the polymer substrata.

Two standard methods have been accepted by the American Society for Testing and Materials, namely protocols ASTM E2562-17 [105] and ASTM E2871-13 [106], which are methods for developing biofilm and for assessing disinfectant efficacy against sessile *P. aeruginosa* cells in a Center for Disease Control (CDC) reactor. In the CDC reactor, Cai et al. [142] tested the anti-biofilm performance of a diazeniumdiolate-doped poly(lactic-co-glycolic acid)-based nitric oxide releasing film applied to indwelling biomedical devices. Li et al. [143] used the CDC reactor to mimic acidogenic meals and snacks of an oral environment in order to test new dentin-composite and hydroxyapatite disks against multi-species oral biofilm. Ganewatta et al. [38] provided a new contact-killing surface by modifying the natural resin acids (from gum rosin) into quaternary ammonium compounds and employed the CDC reactor to prove the strong anti-biofilm activity of the new material against *S. aureus* and *E. coli*. Dell’Orto and colleagues [76] obtained new medical materials by grafting *p*-aminocinnamic or *p*-aminosalicylic acids on low density polyethylene surfaces, and proved their anti-biofilm efficacy against *E. coli* biofilm in the CDC reactor.

#### 4.2.3. Rotating or Spinning Disk (RD) Reactor

Like the CDC reactor the rotating or spinning disk (RD) reactor contains coupons, made of any material, held by a disk attached to a magnet that allows an adjustable rotational speed when the reactor is kept on top of a magnetic stirrer. The disk rotation establishes a liquid shear on the coupon surfaces [134]. Different shear stresses can be assessed at the same time by placing the coupons at different radial orbits. In contrast to the CDC reactor, which allows a quick and easy removal of coupons during the experiment, the RD reactor coupon sampling can only be done by carefully removing the entire disk from the reactor and returning the disk to the reactor for further biofilm studies.

The RD reactor was originally used to evaluate the biocidal efficacy against toilet bowl sessile cells [144]. This method was subsequently developed into the standardized biofilm method ASTM E2196-17 [107] that describes the assessment of a *P. aeruginosa* biofilm grown with shear and continuous flow. For example, Cotter et al. [145] tested the ability of *S. epidermidis* biofilm to grow on polyethylene coupons by the RD reactor. Barry et al. [146] used the RD reactor to accelerate biofilm formation on latex samples from an external male catheter.

#### 4.2.4. Annular (AN) Reactor

The annular (AN) reactor has been used for several decades to develop biofilm in turbulent flowing environments [147]. Indeed, it is well suited to mimicking biofilm formation on a water treatment process surface. The AN reactor consists of two cylinders, one a static external cylinder made of actual pipe materials and the other a rotating internal cylinder, its speed of rotation able to be finely adjusted in order to obtain the desired shear stress. The inner cylinder supports the coupons, which are in the form of rectangular slides that can be manufactured from any machinable material [147]. Some annular reactors also have a jacket to set desired temperatures.

For example, Pintar and Slawson [148] tested the incidence of temperature on a disinfectant procedure in a drinking water distribution system, using a bench-scale approach provided by an AN reactor. Indeed, there was biofilm development on the polyvinyl chloride surfaces at all the examined temperatures, but at low temperatures the disinfectant had a less biofilm inhibitory effect. Similarly, Ndiongue et al. [149] used the AN reactor to investigate the effects of temperature and biodegradable organic matter on the free chlorine residual needed to control biofilm accumulation in plastic pipes distributing water. For 15 months Jang et al. [150] investigated the effect of four pipe materials on biofilm growth and water quality, using an AN reactor under hydraulic conditions similar to a real plumbing system. The steel and copper surfaces, suffering progressive corrosion, showed substantially increased bacterial concentrations, whereas the stainless steel and polyvinyl chloride surfaces were revealed to have biofilm growth that was mainly affected by water temperature.

#### 4.2.5. Drip flow (DF) Reactor

This is made of four/six parallel chambers with vented lids, each one containing a glass-slide-shaped coupon or tubes manufactured with a variety of materials. The medium enters the individual chambers through a needle, the reactor being kept at an angle of 10° so that the liquid flows along the length of the coupons or tubes [151]. The drip flow (DF) reactor is used in the ASTM Method E2647-13 [108] for the development, sampling and study of *P. aeruginosa* sessile cells grown under low shear and continuous flow, mimicking the environmental conditions found in indwelling devices and the human body (e.g., lung infections, tooth biofilm, microbial colonised catheters). Sawant and colleagues [152] assessed the anti-biofilm capacity of silver nanocomposites, showing that the new materials reduced *E. coli* biofilm development by six orders of magnitude. Pérez-Díaz et al. [84] tested the anti-biofilm properties of chitosan gels loaded with silver nanoparticles on clinical isolate strains. The preparation of a multi-species biofilm of oxacillin resistant *S. aureus* and *P. aeruginosa*, obtained from a human chronic wound infection, was performed employing a standard DF reactor under conditions that mimic the nutrient flow in the human skin. Goodwin et al. [153] used the DF reactor to investigate biofilm development on polymer nanocomposites containing carbon nanotubes when they come into contact with microorganisms in aqueous environments post-consumer use. Zaltzman et al. [58] tested the ability of nanoparticles incorporated in commercial dental resin material to inhibit biofilm formation of the cariogenic *S. mutans* by using a DF reactor.

#### 4.2.6. Flow Cells (FC)

Flow cells (FC) were designed to evaluate biofilm processes directly using microscopy and image analysis in a non-invasive way [154]. The biofilm is grown encapsulated in a reactor (flow) chamber provided with an inspection glass that allows the microscope lens to directly record images of the biofilm [35]. The FC is connected to nutrient and waste carboys by silicone rubber tubing, and nutrients are continuously pumped inside the cells. The employment of fluorescent probes coupled with confocal laser scanning microscopy (CLSM) makes flow chambers especially useful for in situ gene expression studies. Biofilm in FC is exposed to the passage of air bubbles that could cause the detachment of biofilm parts.

Several FC devices of different design have been developed. The coupon evaluation FC are single or dual channel flow cells provided by wells (up to 3 per channel) at the bottom to accommodate coupon samples for testing. Coupons can be customized in all materials, and a standard microscope coverslip is used as a viewing window. The trasmission FC is similar to the coupon evaluation FC except that it is provided with a unique recess able to allocate any irregularly shaped materials (suture, catheter section, porous media, etc.). In the capillary FC, biofilm is grown inside glass capillaries that can be directly put under the microscope. This FC is less suitable for the study of new surfaces as it requires that the new material be cast in glass, in a capillary shape and with high optical properties. The treatment imaging FC is a round cell provided with a unique well designed to favour the quick installation of disc coupons with biofilm pre-grown in the RO, RD and CDC reactors. It is used to provide images of pre-grown biofilm during treatment with biocides and other chemical agents (See Section 6.2.1). The small liquid volume of the cell minimizes the use of valuable chemical compounds. The flow chamber is sealed with a round microscope cover glass.

Jaramillo et al. [155] used FC to test the efficacy of a polystyrene surface coated with benzalkonium chloride against early adhesion and biofilm formation of oral and dental root canal bacteria. Francolini et al. [156] loaded usnic acid, a secondary lichen metabolite, into modified polyurethane. The polymers were then incorporated in a FC and *S. aureus* and *P. aeruginosa* biofilm development was analysed using CLSM. In this research both *S. aureus* and *P. aeruginosa* were first transformed with green fluorescent proteins to make them fluorescent. In another work, Fabbri et al. [157] used a FC device to grow marine biofilms on plastic coupons coated with six different biocidal antifouling coatings and one inert non-biocidal coating for 8 weeks.

#### 4.2.7. Microplate under Flow (Bioflux)

The device combines small volumes and high-throughput ability of microtiter plates with the biological relevance of a laminar flow cell [158]. The system consists of microtiter well plates in which reagents flow through microfluidic channels running between the wells. Indeed, a pneumatic pump forces the fresh medium from the inlet wells to the outlet, through the microfluidic channel in which biofilm develops. The pump provides for a fluid flow of up to 96 individual biofilms, allowing fine control of adjustable continuous or intermittent fluid flow rates. Once labelled with a fluorescent probe, biofilm can be viewed with a microscope or scanned with a plate reader [159]. Indeed, time-lapse CLSM images of biofilm formation can be performed [160]. To the best of the authors’ knowledge, there is no research in the literature about its use to prove the anti-biofilm performance of new materials, the Bioflux device could be suitable to easily study new coatings. However, in order not to obstruct the Biolux channels, the method is suitable for only thin coatings.

### 4.3. Microcosms

Microcosms are simplified systems developed under strictly controlled conditions, used to mimic natural ecosystems with their relevant microorganisms [32]. Therefore, microcosms are the systems that closely replicate the in vivo conditions of the real environment, e.g., wound, oral and stream biofilms [90].

Both static and dynamic systems can be turned into microcosms. In comparison to the previously described methods, microcosms consider more environmental parameters and better mimic the complexity and heterogeneity of natural environments. Indeed, these systems include a high diversity of species and require internal processes to reach and maintain system stability. As the complexity of these systems increases, the interpretation of the outcomes and reproducibility become more complicated, compared to both static and dynamic approaches [154].

Abdulkareema et al. [161] coated material for dental implants with zinc oxide nanoparticles and hydroxyapatite. The authors determined the anti-biofilm activity of the new material by a microcosm system using human saliva as an inoculum and artificial saliva and peri-implant sulcular fluid as medium. Li et al. [162] studied the effect of salivary pellicle on anti-biofilm activity of novel dental adhesives by means of a dental plaque microcosm biofilm model using mixed saliva from 10 donors. Similarly, Zhang et al. [83] studied the effect of water-aging on novel anti-biofilm and protein-repellent dental polymeric composite for 180 days using saliva from 10 healthy human donors who had not brushed their teeth for 24 h and had no food intake for 2 h.

## 5. Quantitative Analysis of Biofilm on Anti-Biofilm Polymeric Surfaces

**Figure 3:**
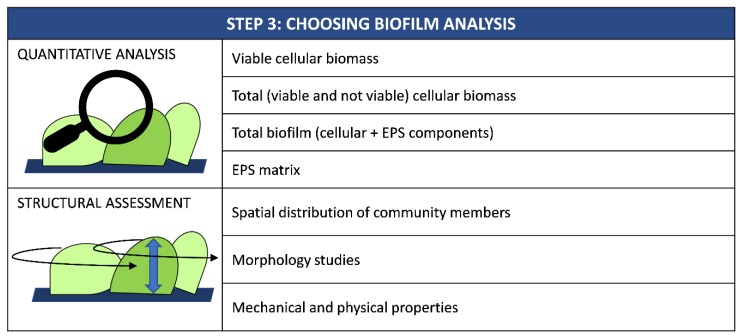
Scheme representing the third step in the experimental procedure for testing new anti-biofilm materials. The choice of the type biofilm analysis includes quantitative and structural assessment assays.

Once biofilm has been grown on a new surface, it is necessary to quantify its biomass to assess the material’s anti-biofilm performance (Figure 3).

Quantification methods include those suitable to assess only viable biomass, those able to detect both live and dead cells as well as techniques able to investigate the whole biofilm, including both cellular and EPS components. Indeed, the most appropriate method to be chosen depends on the type of materials (Table 2).

### 5.1. Viable Cellular Biomass

#### 5.1.1. Plate Count Assay

The most widely used technique to quantify a surface’s viable biomass is to determine the colony forming units (CFU) on agar media, after biomass detachment from the surface [35]. The CFU technique is generally accepted as a ‘gold standard’. However, the detachment procedure is a weak point as a soft procedure does not ensure the full detachment of all the sessile cells while a harsh approach, e.g., sonication, can injury cell viability and thus compromise the cell count, possibly resulting in false negative results [35]. Furthermore, many strains cannot be cultured and several cells within the biofilm, the persister cells, persevere in a non-growing, metabolically inactive state, and thus cannot be detected by the CFU approach [163]. Another important point is that CFU counting can be a valuable tool for quantifying bacterial biofilm, but it is not really suitable for the fungal biofilm that develops in filamentous structures.

#### 5.1.2. Biomarker Quantification

##### Phospholipid Fatty Acids

Phospholipid fatty acids are universally distributed, and are made at a relatively constant rate among the membranes of Bacteria and fungi. Therefore, their measurement has been proposed as an accurate estimation of biomass within a biofilm [164]. Indeed, since phospholipids degrade rapidly upon cell death, they represent only the viable microbial community [165]. Indeed, the analytical identification of phospholipids can also provide early indications of the structure of the microbial community and a quantitative biomarker of microbial response to environmental stressors [166].

Many analytical methods that successfully realize the qualitative and quantitative analysis of phospholipids have been developed. Gehron and White [167] introduced a protocol based on the employment of glycerol phosphate for measuring phospholipid concentration and microbial biomass. Their procedure involves the acid hydrolysis of the phosphate from lipid glycerol, and the analysis of labile glycerol by gas chromatography coupled with mass spectrometry (GC/MS) [168]. These methods are well-established techniques for fatty acid analysis, and are the best in terms of high sensitivity and specificity, high throughput and high accuracy. In the last decade, the development of both electron and chemical ionization technologies has significantly increased the performance of MS analysis, providing rapid analysis without derivatization or additional sample handling [169].

The main drawbacks of phospholipid fatty acid determination is the microbial membrane’s limited recovery rate in the extraction procedure, the amount of background lipid contamination and the sensitivity of the analytical equipment [35]. Moreover, the technique is unsuitable for microorganisms with membranes that are not composed by phospholipids, e.g., Archea [170].

##### Ergosterol

Ergosterol, a major component of fungal membranes, is another proper indicator for the quantification of viable fungal biomass. Based on the assumption that ergosterol is labile and undergoes rapid degradation upon cell death, many researchers employ this molecule as an indicator exclusively for living fungal biomass [171]. Ergosterol has been successfully quantified by GC/MS [172] as well as by high-performance liquid chromatography (HPLC) [171,173]. Indeed, conversion factors for microbial biomass have been obtained using representative fungal species.

#### 5.1.3. Metabolic Assays

##### Colorimetric Dyes

Most common assays are based on the conversion of specific substrates to a colored product measurable with a spectrophotometer. After microbial uptake, the dyes are transformed into fluorescent compounds [174]. FDA (fluorescein-diacetate), resazurin (7-hydroxy-3H-phenoxazin-3-one-10-oxide), and tetrazolium dyes XTT (2,3-bis-(2-methoxy-4-nitro-5-sulfophenyl)-2H-tetrazolium-5-carboxanilide inner salt) and MTT (3-(4,5-dimethyl-2-thiazolyl)-2,5-diphenyl-2H-tetrazolium bromide) are examples of metabolic dyes. FDA is degraded by cellular esterases to become fluorescent yellow while resazurin, a blue dye reduced by metabolically active cells, becomes pink resorufin, also fluorescent [175]. In XTT and MTT assays, an electron transport system across the microbial plasma membrane converts the yellow tetrazolium salt to insoluble purple formazan [176].

A significant limitation of these metabolic assays is the fact that the microorganisms in biofilm do not all display the same metabolic activity, and some of them live in a dormant non-metabolic active state. Moreover, metabolic colorimetric-based assays are often calibrated against planktonic cultures, introducing significant error as the metabolic rates differ greatly between the planktonic and the biofilm states [175].

##### Adenosine Triphosphate (ATP) Bioluminescence

Adenosine triphosphate (ATP) reacts with luciferin when the catalyst-luciferase enzyme is present, and the effect of this oxidation reaction is the emission of light, recorded by a luminometer and quantified in relative light units [177]. The presence of ATP on the surface is a proxy of metabolic activity and consequently of microbial viability and biomass [177]. One of the major advantages of ATP detection is that it is fast and easy to carry out. However, ATP biolumiscence is an invasive assay that requires biofilm destruction. Recently, some researchers introduced a non-destructive bioluminescence assay, by producing recombinant bacteria bearing a plasmid for the endogenous production of luciferase [178]. The luminescence produced, proportional to the number of microorganisms, can be quantified via a luminometer. Other disadvantages include the inability to differentiate extracellular and intracellular ATP. Therefore, prior to any experiments, the biofilm needs to be washed with water or buffer to remove extracellular ATP. Furthermore, it is not so suitable for multi-species biofilm analysis as variation in has been observed in ATP production among diverse microbial taxa [179].

##### Isothermal Microcalorimetry (ICM)

Isothermal microcalorimetry (IMC) measures the heat production of biological reactions, which is directly linked to the overall metabolism [180,181]. IMC is a label-free technique that allows precise measurements in conventional, solid, and opaque media. Isothermal microcalorimeters measure less than a microwatt of heat flow possible. Consequently, IMC can detect metabolic activity from as few as 10^4^ microbial cells [180]. Notably, it is suited to investigate microbiological samples in complex or heterogeneous environments as it does not necessitate optical clarity of the specimen [182]. No less important is that the samples need little preparation and after IMC measurements, the undisturbed samples can be studied by other techniques.

##### Tunable Diode Laser Absorption Spectroscopy (TDLAS)

Tunable diode laser absorption spectroscopy (TDLAS) allows non-invasive measurement of the microbial metabolic rate. TDLAS is used to detect change in the O_2_ and CO_2_ concentrations in biofilm systems, which is related to the metabolic activity of growing microorganisms [183]. Despite the TDLAS potential, there are few papers regarding its application to biofilm and its development on surfaces [181].

### 5.2. Total (Viable and Non-Viable) Cellular Biomass

#### 5.2.1. Chamber Counting

At the initial developmental stage, biofilm cell counting can be carried out using microscopy and a chamber counting slide, e.g., Thoma, Burker or Petroff-Hausser chambers. Counting chambers are specialized glass microscope slides able to allocate a defined sample volume, and equipped with a 2D grid at the bottom that can be employed to evaluate the cell density of suspended biofilm [184]. This very simple procedure can be performed with unstained cells, is inexpensive and only requires an optical microscope. However, it is time consuming, requires many counts for reproducibility and is subject to manual counting bias. The biofilm has to be dislodged from the surface, homogenized and suspended in a liquid solution prior to analysis. In mature biofilm a 3D structure is formed, making counting more problematic.

#### 5.2.2. Dye Binding

**Figure 4:**
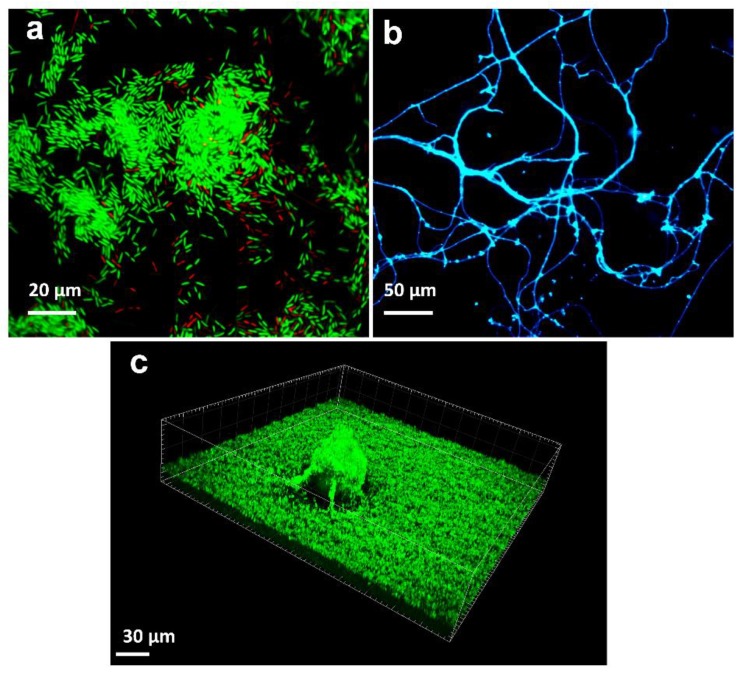
Epifluorescence microscope images of (**a**) *E. coli* biofilm grown on low density polyethylene surface and stained with Live/Dead BacLight; (**b**) *Aspergillus niger* biofilm grown on fluorinated coated glass surface and stained with the Fluorescent Brightener 28 dye; and (**c**) confocal laser scanning microscopy (CLSM) image of green fluorescent protein (GFP) *P. aeruginosa* biofilm grown on polycarbonate surface.

Dye binding to DNA and RNA, such as 4′,6-diamidino-2-phenylindole (DAPI) or Syto9/propidium iodide (PI), 3,3’-dihexyloxacarbocyanine iodide (DioC6)/PI, acridine orange (AO)/PI, carboxyfluorescein diacetate (CFDA)/PI, Calcein/PI, Hoechst/PI and many other combinations of dual staining, can be employed to study both live and dead microbial cells and give an insight into the total amount of microbial sessile cells [185]. Notably, these dyes do not allow discrimination between different microorganism populations in the biofilm. Fluorescence can be detected by both spectrophotometric measurements and microscopic observations [186] (Figure 4a). When relevant, the amount of dye taken up by the cells can be assayed, providing a quantitative indicator of the cellular amount within the biofilm. In most staining combinations, discrimination between viable and dead cells is based only on membrane integrity, so the effect of surfaces modified with molecules not affecting membrane integrity cannot be monitored. Moreover, the following question still remains open: how quantitative and reliable these methods are in the case of heterogeneous multi-species biofilms where variation in fluorescence emission has been observed depending on microbial strains. Stiefel et al. [187] found that staining of *S. aureus* cells with Syto9 alone resulted in equal signal intensity for both live and dead cells, whereas staining of *P. aeruginosa* cells led to 18-fold stronger signal strength for dead cells than for live ones, with an underestimation of viable cells. Indeed, the authors concluded that Gram-negative bacteria were not accessible for Syto9 staining as Gram-positive cells. Additionally, potential interference between the dyes and the surface to be tested needs to be considered, especially when the polymeric surfaces deliver specific anti-biofilm molecules. However, fluorescent staining is considered a fast and easy approach for the quantification of cellular biomass [187,188].

#### 5.2.3. Biomarker Quantification

Quantification of the various components of a cell, e.g., organic carbon, proteins or other molecules, e.g., chlorophyll, have been proposed as alternative methods to indirectly quantify biofilm biomass. The number of such biomarkers is determined and then related to cellular biomass using calibration curves prepared with appropriate standards, under the assumption that their cell content is similar. However, the molecular amount often varies across species, age and culture conditions [184]. Furthermore, the biomarkers and EPS must be separated before biomolecule quantification. Therefore, it is suggested to give these results in tandem with more direct methods, such as CFU or cell counting [184].

##### Total organic carbon

The total organic carbon of biofilm is usually quantified by a two-step process: First, the inorganic carbon is transformed to CO_2_, via heated acidification, and studied by infrared spectroscopy, then the total carbon in the sample is converted to CO_2_, usually via heated oxidation, and measured. The total organic carbon is the difference between the values of total carbon and inorganic carbon [189].

##### Proteins

Cellular lysis releases proteins in solution, these being measured by the change in color due the dye-protein interaction via an ultraviolet–visible (UV–Vis) spectrometer at a particular wavelength. The most commonly used colorimetric assays for protein quantification include the Bradford, Lowry, and bicinchoninic acid (BCA) methods. The Bradford assay consists of adding an acidic Bradford reagent containing Coomassie Brilliant Blue G-250 dye to the lysed sample. During a brief period of incubation, the protein binds to the dye, changing the color from brown to blue. The change in absorbance at 595 nm is recorded, and converted to a total protein concentration through a standard curve [190]. The Lowry method combines the reaction of Cu+, produced by peptide bond oxidation, with the Folin phenol reagent. The result of this reaction is the reduced Folin reagent (heteropolymolybdenum Blue), an intense blue molecule of which the concentration, measured by absorbance at 750 nm, is proportional to the protein amount [191]. Like the Lowry assay, at the beginning the protein complexes with copper ions. Then, this protein-bound copper chelates BCA, resulting in a deep purple color linear with that of the amount of proteins [192]. BCA provides a more uniform response to proteins than the Bradford assay, but it is strongly affected by the amino acids tyrosine, tryptophan, and cysteine. Moreover, chemicals that react with copper (such as ammonia) can affect with the BCA assay.

##### Chlorophyll

Chlorophyll-*a* is one of the most employed biomarkers for quantifying microalgal and cyanobacterial biomass [193,194,195,196] compared a number of methods for cyanobacterial biomass quantification on surfaces and confirmed that chlorophyll-*a* is a good estimator of sessile biomass. Chlorophyll-*a* can be determined spectrophotometrically after extraction in DMSO following a protocol described by Fernández-Silva et al. [197]. However, this method requires an invasive manipulation of the attached cells, and a destructive sample preparation. Alternately, biofilm biomass can be achieved by measuring chlorophyll fluorescence [198]. Recently, pulse amplitude modulated (PAM) fluorometry was used by Vázquez-Nion et al. [199] to measure the in vivo fluorescence signal in a non-destructive way. By measuring the minimal fluorescence signal of dark-adapted cells and the maximal fluorescence signal after a saturating light pulse in dark-adapted cells, the maximum photochemical efficiency of photosystem II, an indicator for the general level of fitness of the photosynthetic organisms, can be quantified. Moreover, the authors developed a standard curve that allowed the correlation of the minimal fluorescence signal of dark-adapted cells with the amount of chl-*a* content as a biofilm biomass estimator.

#### 5.2.4. Quantitative Polymerase Chain Reaction (qPCR)

Quantitative polymerase chain reaction (qPCR) has been used to study the total cellular portion of the biofilm community, evaluating the total amount of DNA from both live and dead cells [200]. Therefore, qPCR is not suitable to evaluate the anti-biofilm performance of surfaces with anti-microbial activity as it does not discern between live and dead cells. Indeed, cells might be killed but not removed from these surfaces.

This technique has limitations in that it tends to underestimate or overestimate the microbial count. Indeed, qPCR detects all the DNA in a sample, including that found in the environment [201]. To avoid the DNA quantification of not living cells, prior to DNA extraction, samples can be treated with nucleic acid intercalating dyes, such as propidium monoazide (PMA-qPCR), able to bind free extracellular DNA (eDNA) and DNA from damaged cells [202]. PMA intercalates into the DNA to which it can be covalently cross-linked when exposed to light, resulting in the suppression of PCR amplification [203].

#### 5.2.5. Flow-Based Cell Counting

##### Coulter System

A more automated way to count cells involves the use of systems in which cells in liquid culture flow through narrow apertures and are measured as they pass. In the Coulter system, charged particles in an electrolyte solution alter the impedance of an electrical circuit when they pass through the aperture. Changes in voltage are correlated to particle size and, if pulsed, are counted over a period of time, providing a cell number [184]. Although simple, the technique cannot discriminate between live and dead cells. Moreover, it requires the biofilm to be homogenized and suspended in liquid solutions.

##### Flow Cytometry

In flow cytometry, cells, previously marked with a fluorophore, flow through a narrow opening and a laser detects them as they pass via scattering, absorbance or intrinsic and extrinsic fluorescence measurements [184]. Flow cytometry accurately assesses the cell fractions, e.g., live and dead cells. Therefore, it is well suitable to study biofilm response to antibiotics and other cytotoxic chemicals [204]. Moreover, additional information about the cells, such as dimensions, surface properties, metabolic activity and growth state, can be recorded using specific fluorescent tags [184]. The technique allows the observation of several thousands of cells in a matter of minutes, providing statistically relevant results for the analysis of sessile populations [205]. However, it is expensive and requires specific equipment and highly skilled operators. Moreover, the cell counting is often limited by cells that adhere tightly one to the other [185].

### 5.3. Total Biofilm (Cellular + Extracellular Polymeric Substances (EPS) Components)

#### 5.3.1. Dry Weight

The simplest way to quantify total biofilm formation on a new anti-biofilm surface is to measure its dry weight. Measurements can be obtained by calculating the difference between the weight of surface material with biofilm and that of the same surface before biofilm formation. Dry mass is obtained by placing the biofilm in an oven at a constant temperature until water removal. Alternatively, if the biofilm surface is heat sensitive, the biofilm can be removed and suspended in a physiological buffer, precipitated with cold ethanol, and the precipitate collected for investigation [184]. Although not highly accurate, weight measurement is a very easy technique.

#### 5.3.2. Optical Density

The reduction in intensity of a transmitted light beam, reported as optical density, is used to measure biofilm total biomass. Indeed, optical density correlates with microbial total carbon and cell mass within a fixed range of cell size and shape [206]. The biofilm is detached from the surface, dispersed in a buffer and the total biomass measured by reading the optical density at 600 nm [207]. Standard curves for cell mass vs. absorbance can be generated for any combination of reactor, microbial strain and spectrophotometer, always allowing determination of mass density by optical density.

#### 5.3.3. Dye-Based Methods

Originally described by O’Toole and Kolter [208] to select biofilm-deficient mutants, these methods have a standard for quantifying biofilm in microtiter plates, owing to the easy use and relatively low cost. Crystal violet (CV) staining is one of the first methods used for biofilm biomass quantification [174]. Alternatively, Safranin Red and Congo Red could be used to quantify total biofilm biomass [207]. These dyes bind to negative charges and therefore target many different molecules of microorganisms and EPS [174]. Staining protocol are detailed reported by Stiefel et al. [207]. The amount of desorbed dye, measured by a spectrophotometer, is directly proportional to biofilm cell amount. Notably, the nonspecific nature of the stains does not allow species differentiation in multi-species communities. In spite it is widely used, CV has major drawbacks, including non-specific binding to anionic proteins and other negatively charged molecules, like capsules, lipopolysaccharides, and DNA/nucleic acids [34]. Additionally, this method provides no information on the actual number of living cells because both the living and dead cells, as well as the biofilm matrix, are stained [175]. Indeed, CV is quite unsuitable to evaluate the anti-biofilm efficacy of surfaces with biocidal activity. Some drawbacks of these assays also include the need for washing steps to remove the unattached cells and the unbound dye, which can lead to the detachment of some biofilm cells [35]. Finally, some studies have demonstrated that composition of cultural media dramatically alters the staining patterns, highlighting the importance of setting suitable biofilm growth conditions especially when a comparison between samples is expected [34].

#### 5.3.4. Color Measurements

On site color measurements can be applied to quantify phototrophic biomass even before the naked eye detects the presence of biofilm [194]. The first intuitive approach to the employment of color variations for evaluating changes in biomass was reported by Young et al. [209]. However, it was Prieto et al. [210] who, for the first time, proved the correlation between modifications in the number of organisms and changes in the parameters defining color. Indeed, most works [199,211,212,213,214] performed color measurements using the CIELAB color system [215], which represents each color by means of three scalar parameters: L*, lightness or luminosity of color; a*, associated with changes in redness-greenness; and b*, associated with changes in yellowness-blueness. Each color can also be represented by three angular parameters: L*, lightness or luminosity of color, defined in both scalar and angular color sets; C*_ab_, chroma or saturation, related to the intensity of color; and h_ab_, hue angle or tone of color, which refers to the dominant wavelength [214]. Sanmartín et al. [212] confirmed that CIELAB color coordinates significantly correlated with the chlorophyll-a, phycocyanin, allophycocyanin, and ATP contents. Color measurement has successfully been applied not only to quantifying biofilm growth, but also as a useful tool to assess the physiological state of phototrophic organisms on solid surfaces [216].

### 5.4. EPS Matrix

The amount of biomass retrieved from a substratum is an indicator of the anti-biofilm features of a new material. In fact, new materials can also act by destabilizing the biofilm matrix and its physical integrity. Therefore, EPS is a must in the assessment of the anti-biofilm performance of new materials.

In addition to water, EPS is made of extracellular polymeric substances, mainly polysaccharides, proteins, lipids and DNA. Characterization of the matrix requires the identification and quantification of each constituent. Generally, the analysis of molecules in the matrix can be investigated by ex situ and in situ methods [217].

#### 5.4.1. Ex Situ EPS Analysis

##### EPS Extraction

Ex situ quantification of EPS compounds greatly depends on the extraction methods. Indeed, fractions of exopolymers, colloidal and capsular, can be extracted from each biofilm. The colloidal fraction includes compounds that are loosely bound to microorganisms, while the capsular fraction contains tightly bound carbohydrates and proteins [218].

EPS extraction is a challenge due to the different physicochemical properties. Moreover, it is necessary to detach EPS from microorganisms without destroying the cells. The physical methods include ultrasounds, blending, high speed centrifugation, steaming, heating, cation exchange resin or lyophilization, whereas the chemical ones include the use of chemical reagents such as ethanol, formaldehyde, formamide, NaOH, EDTA or glutaraldehyde [219]. Cation exchange resin is another effective technique that has been used to separate EPS from cells [220,221]. No consensus exists on the best methodology as the amount and quality of recovered compounds depends on biofilm species and EPS complexity. A combination of physical, chemical and mechanical methods is often the best solution to ensure extraction of EPS enriched fractions with few contaminates of intracellular content [196,222]. Indeed, an adequate extraction protocol often depends on the scientific goal to be addressed.

Comparing five extraction methods of EPS from alga-bacteria biofilm, Pan et al. [223] found that biofilm pre-treatment with ultrasound at low intensity doubled the extracted matrix yield without significant changes in the composition of EPS. The addition of NaOH to EDTA or formaldehyde increases yield extraction of about one order of magnitude compared to the extraction performed with only EDTA or formaldehyde. Liu et al. [224] matched estimated EPS quantities extracted by formaldehyde–NaOH with CLSM observations and found that formaldehyde–NaOH extract limited only a portion of proteins and polysaccharides. Indeed, sonication coupled with formaldehyde treatments is more efficient for extracting proteins, while EDTA is better for extracting polysaccharides and humic acid substances [224]. McSwain et al. [220] found that the use of NaOH and heat extraction produces a higher protein and polysaccharide amount from cell lysis, highlighting the importance of finding a good extraction procedure, as contamination by cell lysis and dead biomass leads incorrect conclusions.

Once extracted, the amount of extracellular components can be quantified.

##### Proteins

Protein quantification has principally been performed by colorimetric methods, following the same procedure reported in Section 5.2.3. For example, the Bradford assay was used successfully by Villa et al. [225] to measure the amount of proteins in the matrix of a colony biofilm growing on a polycarbonate membrane. Similarly, Cattò et al. [226] quantified the amount of proteins in the *E. coli* biofilm EPS grown on polycarbonate coupons in the CDC. Alternatively, in Jachlewski et al. [219] extracellular proteins were quantified by a modified Lowry assay.

##### Polysaccharides

The phenol-sulfuric acid method proposed by Masuko et al. [227] is the simplest and most rapid colorimetric method to evaluate total carbohydrates in a specimen. The method detects virtually all classes of carbohydrates, including mono-, di-, oligo-, and polysaccharides. In this method, the concentrated sulfuric acid breaks down any polysaccharides that react with phenol producing a yellow-gold color. The yellow-gold color is proportional to the amount of total polysaccharides in the sample and can be measured by absorbance at 490 nm. The amount of polysaccharides can be estimated by constructing a standard curve using xylose or glucose as a standard. The color is stable for several hours, and the accuracy of the method is within ±2% under proper conditions [228]. Moreover, it is suitable for high-throughput screening in microtiter well plates.

The major disadvantage of the method is that it does not provide really exact quantitative values as different sugars cause unequal responses [229]. Furthermore, it does not distinguish the different monomeric, oligomeric, or polymeric carbohydrates in the samples. Therefore, a suitable cultivation medium is fundamental to obtain reliable outcomes [229]. Indeed, complex media containing carbohydrate compounds should be avoided or eliminated so as to avoid false values. Additionally, the use of hot temperature and concentrated sulfuric acid and phenol necessitates special precautions with regard to personal safety and laboratory equipment. Consequently, in the last decade, variants of the method have been proposed by removing the carcinogenic phenol reagent or by reducing the reaction time and removing the heat incubation step. A detailed overview and description of different colorimetric modifications to quantify total polysaccharides is discussed extensively in Rühmann et al. [229].

The screening of specific carbohydrates or fractions has also been proposed to study EPS [230,231,232]. One possibility is to screen uronic acids by the hydroxydiphenyl assay [233] using alginate as standard. In the presence of m-hydroxydiphenyl, uronic acids give a color reaction that is specific for mannuronic-, glucuronic-, and galacturonic-acids. As mannuronic-, glucuronic-, and galacturonic-acids show different responses, the uronic acid quantification is reliable only when a known uronic acid is present in the biofilm [229]. Furthermore, high concentrations of neutral sugars or proteins could cause erratic results [234].

##### Extracellular DNA

Different approaches have been used to extract extracellular DNA (eDNA) from a matrix; these include the use of ionic exchange, chelating agents, anionic surfactants, a strong alkaline solution and enzymes [235]. In any case it is quite difficult to extract eDNA from the biofilm matrices without contamination from the genomic DNA released after cell death. Moreover, eDNA binds with other biopolymers such as polysaccharides, proteins and metabolites, making its extraction from the complex matrix even more difficult [236].

Once extracted, the easiest approach to quantify eDNA is by reading eDNA absorbance at 260 nm, and assessing its purity by calculating the ratio between the absorbance at 260 nm and 280 nm [237]. Alternatively, nucleic acids can be stained with Syto9 or DAPI and the intensity of fluorescence intensity measured [238].

##### Antibody Microarrays

Antibody microarrays provide a fast and reliable analysis of up to hundreds of biomarkers simultaneously. In the fluorescent sandwich microarray immunoassay, selected antibodies (primary antibodies) recognize and bind to specific EPS proteins and polysaccharides with high specificity. Detection of this binding is accomplished by directly linking a variety of fluorophores to the primary antibody or introducing them through a secondary antibody that recognizes the primary antibody. Fluorescence intensity is measured and the experimental values can be transformed into a binary matrix and visualized as a heat map. Furthermore, clustering analysis permits the association of similar immunoprofiles or patterns with samples from apparently very different environments, showing that they share similar universal biomarkers. While this assay can only detect predefined antigens without providing precise characterization, it still works as a rapid fingerprinting technique for comparing different samples [218]. In recent work, Blanco et al. [218] used 80 diverse antibodies labeled with the fluorophore Alexa-647 to provide a protein and glycosidic immunoprofile of the capsular EPS fractions extracted from 20 biofilms taken from five extreme environments. Antibodies were set up to detect specific bioanalytes from Bacteria, Archaea and Eukarya, and specific proteins related to iron storage, transporters or metal reductases.

##### Spectroscopic Techniques

Biofilm matrix can be analyzed by vibrational spectroscopic techniques like attenuated total reflection/Fourier transform infrared spectrometry (ATR-IR) [217,239,240] and Raman spectroscopy [241,242].

Indeed, ATR-IR has been used to determine the chemical composition of biofilm by an analysis of the absorbance of infrared light by specific chemical groups. ATR-IR enables studies of the biofilm matrix, eliminating artefacts that can arise during the processes required to isolate specific matrix components. Raman spectroscopy measures photons scatterring from the molecule, which depend on the types of bonds in the molecule. Raman spectroscopy provides fingerprint information about the analyte of interest. As the Raman spectrum of each molecule is unique, the spectrum can be used to identify specific molecules within the biofilm. Diffraction-limited spatial resolution of Raman spectroscopy is around 1 μm, enabling detailed analysis of a complex mixture of various molecules such as proteins, carbohydrates, lipids, and nucleic acids [241]. Sample preparation is easy and there is no need for staining procedures. However, the acquisition of spectra from biomolecules can take a longer time and require higher laser power, leading to damage of the sample. In modified Raman spectroscopy-based techniques such as surface-enhanced Raman scattering (SERS), the sensitivity is significantly increased, the detection threshold is lowered and it is possible to detect EPS components that are not detectable by Raman microscopy [243].

##### Advanced Techniques

More sophisticated approaches allow the single components of EPS, or the entire composition, to be studied. Included are chromatographic techniques such as gel permeation chromatography [244] and HPLC [245]. The combination of liquid chromatography with UV and electrospray ionization ion trap detection (LC–UV-ESI-MS/MS) performs very well in the qualification and quantification of various sugars. Indeed, this method allowed the simultaneous analysis of hexoses, pentoses, deoxy, and amino-sugars, uronic acids as well as different sugar modifications in one single run [246]. GC/MS gave a detailed overview of the entire EPS composition [245,247] as well as of single components [239]. Matrix-assisted laser desorption/ionization (MALDI)-MS has been successfully used to detect and identify polysaccharides, proteins and lipids in complex biofilms [248,249]. Nuclear magnetic resonance (NMR) gave information about the extracellular polysaccharide composition, including the sequence of repeated units or linkages between the heteropolysaccharides [250]. Blanco et al. [218] measured the total concentrations of nine recoverable metals (Zn, Cu, Fe, Co, Ni, As, Cd, Cr and Pb) using X-ray fluorescence reflection (TXRF) and inductively coupled plasma-mass spectrometry (ICP-MS).

All these techniques have greatly advanced the state of art of biofilm matrix knowledge. However, they require complex and expensive equipment, long times to define experimental conditions, and a high level of expertise not commonly found in any laboratory. In addition, complex preparation steps of the samples are sometimes needed.

#### 5.4.2. In Situ EPS Analysis

##### Microscopic Techniques

Fluorescence microscopy is the pioneering technique used to analyze in situ EPS biofilm. Indeed, CLSM is a powerful tool to assess the EPS on material surfaces (see Section 6.2.1 for details) [251]. Proteins, polysaccharides and DNA can be stained by specific dyes and visualized by the microscope. A semi-quantitative analysis of acquired pictures provides information about the general architecture and the EPS distribution, generating reconstructed 3D images of biofilm grown on a new anti-biofilm surface. Indeed, it is possible to approximate the amount, concentrations and coverage of the various components by correlating the fluorescent intensity of the biofilm components to standard solutions. Examples of protein dyes include the SYPRO Ruby, the 3-(4-carboxybenzoyl)quinoline-2-carboxaldehyde (CBQCA), or the NanoOrange [207]. Polysaccharides can be stained by the Calcofluor White that binds to β-linked polysaccharides such as cellulose and chitin [252] (Figure 4b). Alternatively, ConA-FITC binds α-D-mannopyranosyl and α-D-glucopyranosyl residues in amylopectin and dextran [207]. Fluorescein isothiocyanate (FITC) should label proteins by the reaction of the isothiocyanate group with primary and secondary amine groups [253]. Alternatively, EPS compounds can be visualized with specific antibody labelled with fluorescent probes [254]. Fluorescence microscopy offers high-quality images but limited chemical information.

##### Spectroscopic Techniques

Spectroscopic techniques can be used in situ to characterize biofilm matrix. The crystal of the ATR-IR can be modified using a specific coating and the real time growth of biofilm on the surface can be monitored. For example, Sportelli et al. [255] deposited a thin film of silver nanoparticle Teflon-like composite on the infrared inactive region of the waveguide of the ATR crystal. The authors used ART-IR techniques to follow, in real-time, the inhibition of sessile *P. fluorescens* cells induced by the bioactive silver ions released from the nano-antimicrobial coating. Notably, ATR-IR is a powerful tool to assess the activity of materials with antimicrobial properties, as well as being suitable to study living bacteria. Furthermore, recent developments in instrumentation have allowed Raman spectrometry to define the matrix chemistry in a given location on a specimen with high spatial resolution (down to a few micrometers). For instance, Feng et al. [256] in situ characterized *P. aeruginosa* biofilm cultivated on a chip substrate made of polydimethylsiloxane on a microfluidic platform.

## 6. Structural Assessment of Biofilm on Anti-Biofilm Polymeric Surfaces

### 6.1. Identification and Spatial Distribution of Biofilm Community Members

#### 6.1.1. Fluorescence In Situ Hybridization (FISH)

FISH can be used to investigate the spatial organization of mixed microbial communities, especially in environmental samples, providing also semi-quantitative data of the taxa composition. This can give an insight into how microorganisms interact with each other and the key players occurring in biofilm. Standard FISH uses labeled nucleotide probes for the in situ identification of microorganisms by hybridization to the ribosomal RNA of fixed cells, which can subsequently be visualized and quantified by the microscope. Online databases containing probes developed for FISH are currently available [257]. Using probes with different fluorescent properties it is possible to distinguish and identify microorganisms of different taxa in a mixed biofilm, and assess their spatial organization by microscopy [258]. However, the method shows limitations related to cell permeability, hybridization affinity and target site accessibility, leading to sometimes poor signal-to-noise ratios and lack of target site specificity and sensitivity [259]. Indeed, cells with low rRNA copy numbers, slow growing, or starving, e.g., those in biofilms, often lie below the detection limits or are lost in high background fluorescence [258].

Peptide nucleic acid probes (PNA-FISH) present a quicker and stronger binding to DNA/RNA [259] than standard FISH. The hydrophobic nature of the PNA molecule allows easy cell penetration, and, theoretically, better diffusion through the biofilm matrix. Alternatively, in locked nucleic acids (LNA-FISH), a synthetic RNA analog with the ribose ring locked to a C3′ endo-conformation is used as higher affinity, specificity, thermal stability and resistance to degradation in comparison to traditional probes [260].

Improvements have also been introduced to enhance the probe fluorescent signals. In the catalyzed reporter deposition-FISH (CARD-FISH) the fluorescence signal is amplified from 26 to 41 times via the immobilization of multiple radicalized fluorescent tyramides by the enzyme horseradish peroxidase conjugated to the oligonucleotide probes, making the visualization of hard-to-detect cells feasible [261]. However, CARD-FISH is rather expensive, time-consuming and requires a harsh sample preparation protocol, with an enzymatic pretreatment and numerous washing steps that compromise biofilm integrity [262]. In other approaches, probes are double (double labeling of oligonucleotide probes FISH, DOPE-FISH) or multilabeled (Multilabeled FISH, MIL-FISH), so that the fluorescent signal is amplified several times [258,263]. Probes can be also combined in order to increase the number of target microorganisms. In the combinatorial labeling and spectral imaging FISH (CLASI-FISH) microorganisms of interest are labeled with a repertoire of monolabeled oligonucleotide probes carrying fluorochromes of closely overlapping spectra [264]. The combination of emitted wavelengths is recorded by spectral imaging with CLSM. CLASI-FISH allows the simultaneous identification of tens to potentially hundreds of microbial taxa in a single microscope image [265].

FISH combined with microautoradiography (FISH-MAR) has been used to make suggestions about metabolic phenotypes of microorganisms [266]. Indeed, biofilm is grown with radio-isotope labeled substrates and FISH reveals radioactivity in cells that have taken up the particular substrate, and the MAR image is then overlaid with the FISH image, providing a specific substrate uptake profile of the individual bacterial cells in the microbial community [267]. However, some chemical elements, e.g., nitrogen or oxygen, do not have a radioactive isotope with a suitable half-life time for use as a labeled tracer for FISH-MAR experiments, and thus cannot be monitored. Similarly, the combination of FISH with the Nano-scale Secondary Ion Mass Spectrometry (FISH-NanoSIMS) allows the simultaneous exploration of the microbial distribution, and quantifies the utilization of C and N isotopes, allowing the detection of the metabolic activity of identified microorganisms at the single-cell level [268].

Alternatively, confocal Raman microscopy (CRM) coupled with FISH (FISH-Raman) can provide information about FISH-identifiable microbes and the non-biological components within the biofilm, e.g., embedded mineral particles and variant chemical composition of the matrix. Unfortunately, to be compatible with Raman spectroscopic techniques, probes must be labeled with quantum dots or radionuclides, with serious trade-offs regarding cost efficiency and experimental safety [269].

The combination of flow cytometry and FISH (Flow-FISH) has also been proposed for high-throughput, rapid and accurate quantification of biofilm cells with simultaneous phylogenetic specificity being provided by the oligonucleotide FISH probes [270].

#### 6.1.2. Immunofluorescence Detection

Immunofluorescence is a very sensitive technique that exploits antibody binding to specific microorganisms in a mixed population, this binding being visualized by the fluorescence emitted by a marker molecule bound to the antibody. The location of the microbial cells can be viewed using a fluorescence microscope [271]. Indeed, antibody-based methods have been demonstrated to be very promising for high specificity and affinity detection in food and clinical samples [272,273]. The technique is also useful to visualize microorganisms bearing specific proteins accessible to antibodies [274]. For example, Vejborg et al. [275] studied the chain formation in *E. coli* K-12 biofilm on vinyl plastic surface, using an immunochemistry procedure to mark the antigen 43, a self-associating autotransporter protein that had already been implicated in auto-aggregation and biofilm development. The authors observed that Ag43 was concentrated at or near the cell poles, and that when the antigen was highly overexpressed a more uniform distribution of bacteria was present within the biofilm.

### 6.2. Biofilm Imaging for Biofilm Morphology Studies

#### 6.2.1. Confocal Laser Scanning Microscopy (CLSM)

CLSM is the most powerful tool to assess biofilm architecture on material surfaces, and as it is readily available in labs and core facilities it has made a considerable contribution to biofilm research [115]. CLSM combined with fluorescent staining and high-speed computing allows the acquisition of two-dimensional cross-sections of a biofilm at different depths, resulting, upon analysis by specific software, in the representation of the 3D architecture [276]. The use of water-immersible lenses that do not require a coverslip can help avoid pressure and distortion of the biofilm structure, offering high resolution with working distances of several millimetres and allowing the visualization of fluorescently labeled single cells and an accurate examination of irregular substrata.

Coupled with the use of various probes, CLSM allows the observation of various biofilm components simultaneously. Table 3 gives an overview of the probes binding the main biofilm components. Indeed, using CLSM in a multi-channel modus it is possible to map the individual biofilm components at the same time, e.g., cellular biomass with Syto9, dead cells with PI, extracellular polysaccharides with Concanavalin A. The coupling of CLSM with FISH probes leads to more information about the identification and localization of several microbial taxa. Another option is to take advantage of the intrinsic natural auto-fluorescence property of some microorganisms, e.g., phototrophic biofilm for imaging differentiation. It is also possible to genetically modify organisms in such a way as to make them auto-fluorescent, for instance through the expression of the green fluorescent protein (GFP) or multicolor variants (Figure 4c). GFP is expressed by metabolic active cells, and can be excited using light at 396 nm, emitting green fluorescence light at 508 nm.

**Figure 5:**
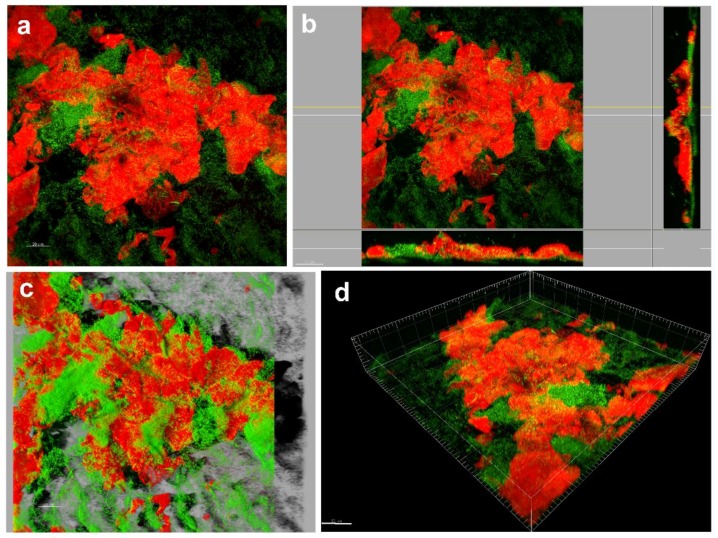
Comparison of different computer visualizations of a single confocal laser scanning microscopy (CLSM) image of *E. coli* biofilm grown on low density polyethylene surface. Computational analysis has been performed by Imaris software. (**a**) maximum intensity projection; (**b**) orthogonal projection; (**c**) blend projection and (**d**) 3D reconstruction. Biofilm was stained green with Syber green I (live cells) and red with Texas Red-labelled ConA (polysaccharidic matrix).

QQuantitative 3D digital image analysis of multichannel data sets allows the volumetric and structural quantification of each single biofilm constituent. Indeed, a number of highly advanced software packages, both commercial and free, coupled with high-speed computers, are available to analyze large data file sets [115,185,276]. Common image analysis software includes Imaris (Figure 5), ImageJ (https://imagej.nih.gov/ij/), Fiji (https://fiji.sc/), Comstat and Phlip [277,278,279]. A comprehensive book on digital image analysis has been edited by Miura [280].

**Figure 6:**
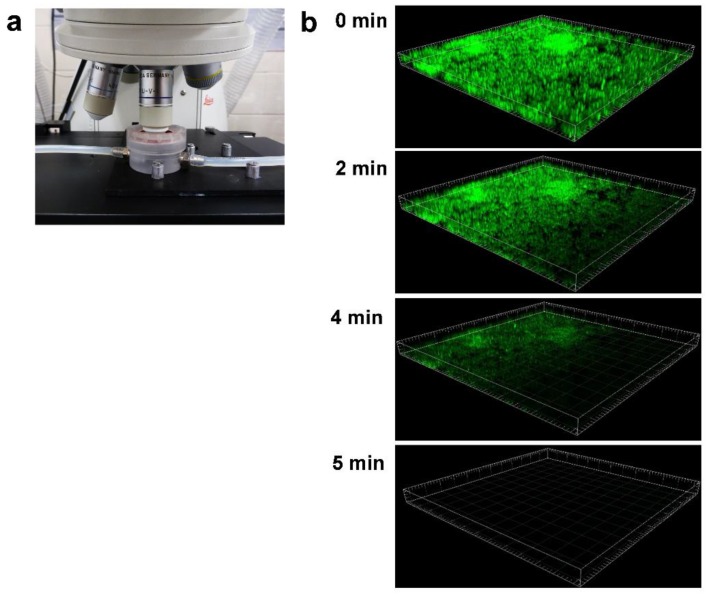
(**a**) Treatment imaging flow cell (FC) under CLSM for time lapse experiments; (**b**) time lapse CLSM of ethanol action performed on *E. coli* biofilm grown on low density polyethylene surfaces functionalized with *p*-aminosalicylic acid. The fluorescence loss from stained *E. coli* cells is used to monitor real-time loss in cell viability during the biocide action. Under ethanol treatment, biofilm displayed a total loss in fluorescent intensity in 5 min.

When assessing a sample, not only should the intensity of the excited fluorochrome can be measured but also its lifetime as this can reveal additional valuable information [281], e.g., the efficacy of disinfection treatment [282]. For example, the evaluation of an accurate threshold value up to which biofilm remains undisturbed on the surface under antimicrobial treatments is valuable information for their direct application in many industrial contexts where biofilm is unwanted. The technique, called time-lapse CLSM, consists in installing a disc coupon with biofilm pre-grown in a biofilm reactor in a Treatment Imaging FC (Figure 6a). Prior to being placed in the flow cell, the biofilm is stained with fluorescent dye that labels viable and dead cells (e.g., the LIVE/DEAD BacLight) or enzymatic activities (e.g., the esterase activity marker calcein acetoxymethyl ester). The imaging technique is based on evaluating the loss of fluorescence corresponding to the leakage of a fluorophore out of cells due to membrane permeabilization by the antimicrobial agent [283] (Figure 6b). In recent work, Cattò et al. [284] used time-lapse microscopy to evaluate the enhanced susceptibility of an *E. coli* biofilm pre-grown on a polyethylene surface functionalized with p-amino-cinnamic acid and p-amino-salicylic acid to ethanol. The method allows the decrease of fluorescence intensity at different locations within the biofilm to be measured, revealing that the ethanol antimicrobial action occurs very rapidly at the biofilm surface and slower where the bottom of the biofilm is in contact with the coupon substratum. Advanced CLSM-based tools including fluorescence recovery after photobleaching (FRAP), fluorescence correlation spectroscopy (FCS) and fluorescence lifetime imaging (FLIM) can also be used to assess the antibiotic diffusion-reaction within a biofilm locally. These techniques trace the diffusion of fluorescent labeled antibiotics within the biofilm, offering a spatiotemporal resolution not available with the commonly employed time-lapse CLSM imaging method [282].

The main disadvantage of CLSM is that the depth of penetration into the sample is limited to around 20-40 µm. Indeed, as depth increases, the scattering and absorption signals of both excitation and emission light lose intensity. Thus, CLSM is not suitable for biofilm of more than 50 µm thickness.

Another limitation of CLSM is the unequal resolution in XY and Z. However, this can be avoided by increasing the objective lens numerical aperture. By employing two opposed lenses and mounting the specimen between two coverslips, equally resolved XYZ data sets can be recorded [281].

Moreover, another major disadvantage of CLSM is the scanning process, which limits temporal resolution, and the need for more excitation energy, resulting in increased photobleaching [285].

However, these drawbacks have now been partially sidestepped as CLSM has been improved tremendously in the last decade with novel optics, lasers, fluorescent proteins and probes as well as CLSM derivatives, such as multiphoton microscopy (MPM), light sheet fluorescence microscopy [286,287] and spinning-disk confocal laser systems [288,289]. For example, instead of using a light source emitting a continuous intensity, MPM uses a laser emitting high-powered (peak power >2 kW) ultrashort pulse (in the femto- or picosecond range), so that two or more photons are able to interact simultaneously with a fluorescent dye molecule at the point of focus [290]. The use of near-infrared excitation light enables MPM microscopy to minimise sample damage and increase the light penetration depth. Moreover, due to multiphoton absorption, the background signal is strongly suppressed. Furthermore, MPM overcomes drawbacks of other optical microscopy techniques such as photobleaching and phototoxicity [291].

#### 6.2.2. Electron Microscopy

Conventional electron microscopy is widely used to achieve imaging of sub-nanometer resolution, providing a detailed insight into the ultrastructure of the biofilm and its environment [306,307]. Indeed, electron microscopy is often applied to visualize the initial formation of biofilm, starting with the single cells attached to an interface [308].

Several electron microscopy techniques have been used to examine the assembly of biofilm structure on solid surfaces, scanning electron microscopy (SEM) and transmission electron microscopy (TEM) being the predominant choices [309,310,311,312,313]. SEM and TEM have been extensively used for a qualitative observation of biofilm, but they are not generally recommended for quantitative evaluations [35,174]. A drawback of both SEM and TEM is the sample preparation, which involves fixation and dehydration, followed by coating with conductive materials such as gold or platinum. As biofilm consists mainly of water, so specimen dehydration could alter the morphology, and changes or artifacts be introduced [311,312,313]. Moreover, the sample is under vacuum, introducing artifacts into the biofilm structure [307].

By sidestepping the hydration and vacuum limitation, in cryo-SEM the biofilm is not dehydrated but kept frozen, thus achieving high-magnification images closer to the native state of the sample [314]. The advantage of cryo-SEM is the lack of pre- operational steps. Ultrafast freezing procedures, e.g., high-pressure freezing, are used for specimen fixation, therefore, sample preparation occurs within a maximum of one minute of time. However, compared to conventional SEM, cryo-SEM images are of poorer resolution due to the low frozen surface conductivity, compared to the dehydrated gold-sputtered surface used in conventional SEM [306]. Moreover, the heat generated by the focused electron beam causes the sample’s frozen surface melts and cracks at high magnifications [314].

Environmental SEM (ESEM) allows the imaging of biological specimens in their original hydrated conditions at relatively high resolution, with a total lack of sample preparation [315]. As in the case of cryo-SEM, ESEM provides images of poorer resolution than conventional SEM, because of the lack of conductivity in the wet sample.

A more sophisticated alternative to create 3D biofilm reconstruction is the FIB–SEM. The FIB sequentially mills away 10 nm-thick sections from the surface of a resin that contains the embedded samples. Images of each slice are subsequently recorded by SEM and processed by specific software to perform the 3D volume reconstruction.

Atmospheric scanning electron microscopy (ASEM) was developed to observe biological samples at atmospheric pressure [307]. ASEM consists of an inverted SEM to observe a wet sample from below while optical microscopy simultaneously observes it from above [316]. Sugimoto et al. [307] cultured a biofilm on an electron-transparent film. The biofilm was directly imaged from below using the inverted SEM, allowing the study of biofilm formation near the substrate. The authors were able to visualize the intercellular nanostructures, including membrane vesicles, cytoplasmic proteins and a thick dendritic nanotube network between microbes, suggesting multicellular communication between microorganisms [307].

#### 6.2.3. Super-Resolution Microscopy (Nanoscopy)

Super resolution microscopy techniques, also referred to as nanoscopy, have been developed to improve the resolving power of diffraction-limited optical and fluorescence microscopy. These tools are able to resolve structures below the limits of optical resolution (200 nm), even down to the 1 nm level [317].

Stimulated emission-depletion (STED) microscopy represents a major type of these techniques and was the first to be proposed and experimentally realized [318]. STED creates super-resolution images giving details smaller than 50 nm by the selective deactivation of fluorophores and minimizing the area of illumination at the focal point [318]. Photoactivated localization microscopy (PALM) and stochastic optical reconstruction microscopy (STORM) take advantage of a similar approach [319]. Instead, in structured illumination microscopy (SIM) and saturated structured illumination microscopy (SSIM) samples are illuminated by a pattern of light at three different angles that make visible the normally inaccessible high-resolution information. The result is a resolution increased of two times in comparison to the optical microscopies [320]. Compared to other super-resolution microscopies, SIM is quite popular as standard fluorophores can be used, does not require special mounting and preparation techniques [321]. The technical details of these methods have been extensively reviewed by Li et al. [321] and Sydor et al. [322].

In spite of their high resolution, all these techniques have certain disadvantages that need to be considered. Super-resolution methods allow the imaging of only thick samples and only a small number of fluorochromes can be used [323]. Moreover, when super-resolution data are processed and analyzed, it is important to choose adequate controls to avoid artefacts and generating high-quality data [323].

#### 6.2.4. Other Microscopic Techniques

CRM is a non-invasive, label-free technique to analyse in vivo biofilm formation. The sample is excited by a laser and the backscattered light signal is recorded, providing information on the chemical composition and conformation. In CRM, no fluorescent probe is necessary and, in contrast to other spectroscopic techniques, is only slightly sensitive to water, allowing the investigation of living specimens in fully hydrated conditions. The main advantage of CRM is that biofilms can be studied in their native, unaltered state, immersed in the medium. Specimen preparation is not necessary and information on the biofilm chemical nature is obtained [324]. A single Raman spectrum contains information about both organic compounds, e.g., polysaccharides, proteins, lipids, humic-like substances, and inorganic constituents like minerals. Specific Raman signals can be extracted and (semi-)quantitatively mapped [325]. CRM has been employed to map distributions of water and biomass [326], pigmented matrix and cellular content [327] and chemical components such as sugars or proteins [328]. Sandt et al. [327] used CRM to study the structure, composition and growth of fully hydrated biofilm grown on glass in a flow cell. Andrews et al. [329] demonstrated that the attachment of 11 bacterial strains on different surfaces was influenced by several extracellular compounds such as lipids, proteins, and nucleic acids, and the effects of these molecules were important to the genus level. A study reported by Wagner et al. [328] showed a change of EPS composition in heterotrophic biofilms over time, which was not detected by CLSM.

Scanning transmission X-ray microscopy (STXM) is extensively employed in mapping biofilm composition without using a probe, and also with reduced radiation damage to the sample. STXM is employed to study polymers as well as metal distribution at a microscale [330,331]. STXM can be applied to fully hydrated biological materials. This is a consequence of the ability of X-rays to penetrate water with reduced radiation damage to samples.

#### 6.2.5. Optical Coherence Tomography (OCT)

Optical coherence tomography (OCT) measures depth-resolved reflection signals from translucent samples such as biofilms, and allows extended sample areas to be examined within millimetres [332]. Furthermore, the rapidity of measurement and the fact that there is no need for probes or fluorochromes are distinct OCT advantages over other conventional imaging techniques for structural description, including CLSM. OCT allows the acquisition of in situ and real time images, in a non-ionizing and non-invasive way [333]. Indeed, the cross-sectional images of biofilms on the micron scale of OCT offer a quantitative, high-resolution, spatially-resolved means to analyse biofilm growth, detachment, thickness, and structural heterogeneity [334]. Other advantages are the small size and mobility of an OCT device, allowing the investigation of biofilm inside the cultivation device itself [327].

Several papers have reported dynamic biofilm development on polymeric membrane surfaces under different operating conditions [334,335,336] as well as on medical devices [333,337] and other materials, e.g., polystyrene [338].

### 6.3. Mechanical and Physical Properties

Studies of the mechanical and physical behaviour of biofilms (e.g., viscosity, elasticity, adhesion, cohesion, permeability, etc.) are crucial to an understanding of biofilm physical stability, and thus of the performance of new anti-biofilm material. Indeed, materials based on matrix-degrading enzymes, or quorum-sensing inhibitors, can change a biofilm’s mechanical and physical properties, making the biofilm more prone to detachment and less resistant to antimicrobial treatments. Therefore, mechanical and physical parameters can be considered quantitative biomarkers of EPS integrity. Furthermore, mechanical and physical features should also be useful to optimize cleaning strategies in an industrial setting, e.g., by determining the best flow stress to override biofilm presence, or to assess the antimicrobial penetration in the biofilm. For example, it has been hypothesized that the formation of viscoelastic extended structures by *S. aureus* in intravenous catheters could block the catheters, and that the breaking of these viscoelastic chains, e.g., by using matrix degrading polymeric materials, could dislodge the biofilm from the surfaces [339,340].

There are a number of varied testing methods available to study biofilm mechanical and physical parameters. Recently, Billings et al. [341] and Boudarel et al. [342] provided a good overview of such methods and guidelines by which the mechanical and physical properties of biofilms can be measured. However, the mechanical properties of biofilm remain a concern in biofilm research because there is a lack of standardized protocols for both mechanical and physical testing and associated identification methods [342]. This complicates the comparison of different materials, and thus the improvement of material engineering processes and screening. Furthermore, none of the existing methods cover the whole spectrum of biofilm behaviour as the properties describing the mechanical capacities of a biofilm are both numerous and varied [343]. Indeed, the combination of in situ experimental measurements and biofilm modelling seems a promising approach to ascertain the mechanical and physical properties of biofilms [344,345,346,347]. Unfortunately, the complexity of biofilm structure makes conventional mechanical and physical testing often unsuitable for engineered materials.

#### 6.3.1. Atomic Force Microscopy (AFM)

Atomic force microscopy (AFM) techniques can be used to measure viscoelastic forces and those driving cell–cell and cell–substrate interactions [348]. AFM consists of a tip held in intimate contact with the surface. When the tip is scanned across the surface, it encounters surface forces and the cantilever is deflected, generating force–sample distance curves [349]. As samples can be examined in their native state, sample preparation is minimal, greatly reducing the development of artefacts [350].

The review of Lau et al. [350] reports a list of biofilm viscoelastic and adhesive studies using AFM. For example, Harapanahalli et al. [351] investigated the influence of *S. aureus* adhesion forces to different biomaterials, noting that the adhesion forces modulate the production of some matrix molecules. Also Feuille et al. [352] used AFM to study the forces guiding the self-association of *S. aureus*, focusing on a key surface protein. El-Kirat-Chatel et al. [353] measured adhesion forces between live bacteria and two copolymers based on tert-butyldimethylsilyl methacrylate dedicated to ship hulls.

These researches have provided a novel insight into the interplay of interaction forces and mechanical properties that govern the behaviour of biofilms, and their response to chemical and physical attack.

The main drawback is that AFM requires physical contact between the probe and the biofilm, which poses a challenge for biofilms grown in closed systems, e.g., in flow cells [354].

Modifications of standard AFM have been made, including AFM-based single-cell force spectroscopy (AFM-SCFS). In AFM-SCFS, the AFM tip is replaced by a single cell to measure cell–cell and cell–solid interaction forces. Taubenberger et al. [355] highlighted that AFM-SCFS is a suitable tool to quantify cell-biomaterial interactions, thus greatly contributing to the optimization of new materials for implants, scaffolding, and medical devices. For instance, Spengler et al. [356] used AFM-SCFS to investigate the adhesion strength between a single cell of *S. aureus* and a solid hydrophobic silane and hydrophilic silicon. The study showed a strong influence of the hydrophobic interaction on microbial adhesion, corroborating the notion that the adhesive strength of bacteria is not a matter of contact area, but rather a matter of which, and how many, molecules of the bacterial cell wall are involved in the contact with the surface.

#### 6.3.2. Rheometry

Measurement of viscoelasticity includes both macro- and microrheology methods that quantify biofilm deformation under constant stress, in a compressive, shear or tensile mode, or measuring the stress needed to maintain constant deformation, also referred as stress relaxation [357,358]. The rheometer consists of either parallel plates or a cone and plate between which the material of interest is placed, providing data about the viscoelastic behaviour of the biofilm. In some studies the biofilms were dislodged from their original growth locations and placed in the rheometer [25,359,360], whereas in others the biofilms were grown directly on a rheometer plate [361,362,363], potentially altering the microarchitecture [363].

Macro-rheological studies have provided a lot of information about the viscoelastic properties of biofilms, but this is at the macro-scale level and often requires an ex situ analysis and an impractical amount of biofilm [354].

Particle-tracking microrheology uses micro-beads, embedded in the biofilm without disrupting the natural state of the biofilm system. The number of beads can be one, in single-particle tracking (SPT), or multiple, in the multi-particle tracking (MPT) technique [341]. In passive particle tracking, the beads are not manipulated by any external perturbation, but move in response to thermal or energy fluctuations. In active microrheology, external forces are used to move the probe particle through the biofilm rather than relying solely on fluctuations in thermal energy [364].

In the last decade, particle tracking micro-rheology has been combined with CLSM [341]. For example, Cao et al. [365], used SPT micro-rheology combined with CLSM to analyze biofilm behavior at different horizontal z-planes (bottom, middle, top) and to track trajectories of green fluorescent carboxylate micro-bead particles segregated in two different biofilm regions (voids and clusters). Similarly, Chew et al. [366] measured the rheological properties of *P. aeruginosa* biofilm at different stages of development, tracking, by CSLM, the natural Brownian movement of the spherical particles with marked intrinsic fluorescence within the sample. Alternatively, Galy et al. [364] used CSLM to track the motion of multiple magnetic beads embedded in growing biofilm.

#### 6.3.3. Quartz Crystal Microbalance (QCM)

Quartz crystal microbalance (QCM) technology has been used to detect both the mass and viscoelastic changes in biofilms, in real time and in a non-destructive way. This technique is based on the measurement of changes in frequency of a system composed of a surface or thin coating adsorbed on a piezoelectric quartz sensor due to the additional of removal of small masses [367,368].

A special variation of this technique is called QCM with dissipation (QCM-D). Compared with conventional QCM, the QCM-D technique is able to simultaneously measure changes in resonant frequency and energy loss, or dissipation, of the system, allowing the detection of mass increase and structural changes of cells at the same time [369,370,371].

QCM and QCM-D are well suited to investigating the processes occurring at or near the anti-biofilm surface during initial cell adhesion, including the binding properties between the cell and the material [367,372,373,374]. Wang et al. [374] modified the QCM-D sensor surface with different glycopolymers and cationic polymers and studied the bacteria−substratum interactions on different polymer-treated sensor surfaces. The authors found that lectin-carbohydrate interactions play a significant role in *E. coli* and *P. aeruginosa* adhesion, compared to other non-specific forces, e.g., electrostatic interactions. Indeed, *P. aeruginosa* adhered to the glycopolymer surface with strong contact point stiffness in comparison to *E. coli*. In another work, Knowles et al. [375] used QCM to understand the protein interactions with a new silica nanoparticle anti-biofilm coating. This study elucidated the effect of nanoscale surface topography on fouling processes.

Both QCM and QCM-D systems are capable of detecting the kinetics of biofilm formation on materials, without differentiating between biotic and abiotic components [376]. Moreover, the miniaturization of the mass sensitive detector permits its integration into industrial plants, including the food industry, water distribution systems and clinical settings [373]. However, the interpretation of QCM and QCM-D is often difficult, especially in the case of microbial adhesion. Indeed, many studies on microbial adhesion and biofilm formation report a decrease in resonance frequency upon microbial adhesion whereas other studies report an increase [377].

#### 6.3.4. Surface Plasma Resonance (SPR)

Surface plasma resonance (SPR) is an emerging technique to investigate the binding kinetics and adhesion of *microorganisms* to various natural and synthetic materials. For instance, Pranzetti et al. [378] used SPR to study the initial stages of bacterial adhesion to surfaces, including non-specific contributions from electrostatic, van der Waals, and hydrophobic forces. In this study, SPR provided real-time observations of both EPS and adhesion of two marine bacteria with different hydrophobicity to model surfaces. Indeed, SPR spectral data reveal kinetics of adhesion depending on both the marine bacterial species and the backbone structures and functional groups of the substrate. Zhang et al. [379] demonstrated by SPR that nanosized TiO_2_ decreased the adhesive ability of both *B. subtilis* cell and EPS, and induced biofilm detachment from different surfaces in some hours. Indeed, the decrease in adhesive strength worked against microbial aggregation. These experiments show that the differences in binding were dependent on the type of surface and microbial strains.

#### 6.3.5. Fluid Dynamic Gauging (FDG)

Fluid dynamic gauging (FDG) has been developed to quantify the in situ and real time strength of soft deposits, immersed in a liquid environment [380]. FDG can be used to estimate the adhesive and cohesive strength of biofilm by means of flow data. For instance, Peck et al. [381] investigated *E. coli* biofilm removal by FDG during cleaning treatments under controlled hydrodynamic conditions. The authors compared three different substrates and found that mature biofilm grown on glass has a stronger surface attachment than that on stainless steel and polyethylene.

#### 6.3.6. Microsensors

Microsensors, consisting of a needle-type sensor with a tip diameter of 10–20 microns, measure the gradient concentration of particular chemicals, e.g., the concentrations of oxygen, carbon dioxide, sulfide, pH, oxidation-reduction potential, ammonium, nitrate and nitrite. These probes are small enough to not significantly harm the biofilm upon entry, providing an in situ direct measurement of the chemical concentrations within the biofilm, in a non-invasive way [382]. Microelectrodes have the advantage of making both spatial and temporal measurements within the biofilm. Moreover, multiple microbial taxa can be profiled using different metabolic fingerprints, and the use of multiple sensors allows a better understanding of how such communities are arranged. By creating an array of microelectrodes, it is also possible to measure multiple chemical signatures simultaneously in the same location [383,384,385]. Microelectrodes have been widely used to analyze biofilm on a wide range of materials, including medical surfaces [386], membranes used in wastewater treatments [387,388] and other polymeric materials such as polycarbonate slides [389]. Indeed, equations for macroscale models to gain an insight into flow and transport processes within the biofilm have received much attention [390,391,392,393].

#### 6.3.7. Magnetic Resonance Imaging (MRI) and Pulse-Field Gradient Nuclear Magnetic Resonance (PFG-NMR)

Magnetic resonance imaging (MRI) is a NMR-based technique that has been used in situ and in vivo to characterize water dynamics within biofilm, e.g., determination of flow velocity, as well as the molecular dynamics and diffusion of biomolecules in biofilm, in a non-invasive way. Notably, MRI is not impeded by biofilm thickness and is thus quite suitable for thick biofilm. Studies of mass transport processes inside biofilm on polymeric surfaces using MRI have included water diffusion measurements in biofilm on a polyether ether ketone plastic disc [394] and the transport and fate of heavy metals in biofilm on plastic surfaces [395].

Alternatively, pulse-field gradient NMR (PFG-NMR) can also be employed for the diffusion analysis of water and small molecules within the biofilm. PFG-NMR employs pulsed magnetic field gradients to obtain information about the average displacement of the spins. With PFG-NMR it is possible, in principle, to measure the self-diffusion coefficients of all NMR-detectable molecules [396].

## 7. Conclusions

In recent years, new techniques to study biofilm grown on polymeric surfaces have been proposed. These include microcosms able to accurately reproduce human conditions, and microfluidics that allow the precise manipulation and control of fluids in microscale channels, typically less than 100 μm, and omic-based approaches revealing physiological differences occurring in the course of sessile development in response to interactions with materials [115,397,398]. However, despite their notable contribution to the study of biofilm, these techniques are still far from being widely applied to study anti-biofilm properties. Indeed, these approaches often require specific equipment, a high level of expertise and often long experimental procedures, which make them less appropriate for screening purposes on a large and/or industrial scale. Therefore, while the development of new microbiological techniques is proceeding fast, the evolution of standards for testing the new anti-biofilm materials is proceeding rather slowly.

A key aspect toward the standardization of testing anti-biofilm materials is the sharing of specific details about the experimental technique employed and the corresponding experimental conditions. This is key aspect to replicate studies at an interlaboratory scale, favouring the spreading of methodologies that are expecting to become the standard for the field in the feature. Already there has been a lot of effort put into developing dedicated public web platforms, like that of MIABiE [399] and BiofOmics [400], for the systematic and standardized collection of guidelines, experiments and data. However, the efforts so far performed are not enough and standardization in anti-biofilm surface testing methods remains a blind spot in the biofilm material community. Indeed, standardization is still a challenging matter as biofilms are living, complex, highly heterogeneous and constantly evolving structures. Such features yield intra-sample and sample-to-sample high variability in results, which is, among others, one of the main obstacles when dealing with standardization [342]. Variability has been even reported in the results of the same test on a biofilm grown in the same conditions [342]. To complicate the scenarios, there is still a need to study the response of biofilms throughout their life-cycle in the different environment. Moreover, studies establishing the validity of laboratory biofilm models are still lacking [401].

Beside the number of issues in the field, the implementation of such standardized methods is technically demanding, and efforts to overcome limitations in their development is the direction of the future. Companies in the field urgently ask for new standardized tests to demonstrate that their new discoveries provide statistically relevant results and motivation for spreading the new products on the market. On the other hand, regulatory agencies need standard methods to assess the performance of the new materials and compare their features with the existing technology on the market.

The literature shows that inexpensive and easy assays can be performed routinely, and are more suitable to be standardized, compared to a more accurate but sophisticated assay. Indeed, although often regarded as over-simplistic, these models have contributed greatly to today’s knowledge of biofilm physiology. Certainly, the direction of the future is to set up simple standardized anti-biofilm procedures that on an industrial scale can establish correlation with real outcomes, facilitating data comparison.

Given the lack of standardized procedures to evaluate the anti-biofilm properties of new polymeric materials, today the only possible approach for the scientific community is to follow common bases and good practices. These guidelines are a preliminary step in the direction of standardized protocols. At the present time, there is no single method for studies on the nature of biofilm on surfaces. Indeed, each method has its drawbacks, but it also has distinct strengths, that are important to consider when interpreting the results. Since biofilms are heterogeneous in their organization and structure, the suggestion is to use complementary approaches to confirm the anti-biofilm activity of a selected material.

## Figures and Tables

**Table 1:** Technical information of the most popular laboratory devices used to study anti-biofilm polymeric surfaces.

Apparatus	Flow Dynamic	Interface	Biofilm Analysis	Advantage	Limitations	Standard Methods
Microtiter plate	Static	Solid–liquid	- Adhesion - Detachment - Susceptibility to antimicrobial	- Easy to use - Cheap - Reproducible - Simple equipment - High-throughput screening - Multiple materials tested simultaneously - Multiple conditions tested simultaneously - Well-designed to investigate early stage of biofilm formation - Comparable experiments among different laboratories	- Not suitable to study late stage of biofilm formation - Sensitive to cell sedimentation - Poor reproducibility of real environment - Close system - Washing step can remove not loosely attached cells - Designed to study biofilms only at solid-liquid interface	None
Calgary biofilm device	Static	Solid–liquid	- Adhesion - Detachment - Susceptibility to antimicrobial	- Easy to use - Cheap - Simple equipment - Reproducible - High-throughput screening - Multiple materials tested simultaneously - Multiple conditions tested simultaneously - Well-designed to investigate early stage of biofilm formation - Reduced interference of cell sedimentation - Comparable experiments among different laboratories	- Not suitable to study late stage of biofilm formation - Poor reproducibility of real environment - Close system - Washing step can remove not loosely attached cells - Designed to study biofilms only at solid-liquid interface - Difficulty to collect individual pegs for cell enumeration	ASTM E2799-17 Standard test method for testing disinfectantefficacy against *Pseudomonas aeruginosa* biofilm using the MBEC assay [104]
Biofilm ring test	Static	Solid–liquid	- Adhesion - Detachment - Susceptibility to antimicrobial	- Easy to use - Reproducible - Rapid - High-throughput screening - Automatic analysis - Very low standard deviation - No need for staining or washing steps - Multiple conditions tested simultaneously - Comparable experiments among different laboratories - Well-designed to investigate early stage of biofilm formation	- Not suitable to study late stage of biofilm formation - Poor reproducibility of real environment - Close system - Expensive - Sensitive to cell sedimentation - Only suitable for thick biofilms - Requires specific magnetic device and scanner - Need of specified software and biofilm index to provide the results - Not suitable for multi-species biofilm - Designed to study biofilms only at solid-liquid interface - the material must display magnetic properties - Not suitable to compare multiple materials simultaneously	None
Real Time xCelligence	Static	Solid–liquid	- Adhesion - Detachment - Susceptibility to antimicrobial	- Easy to use - Reproducible - High-throughput screening - Non-invasive - automatic analysis - Very low standard deviation - No need for staining or washing steps - Multiple conditions tested simultaneously - Comparable experiments among different laboratories - Well-designed to investigate early stage of biofilm formation	- Not suitable to study late stage of biofilm formation - Poor reproducibility of real environment - Close system - Expensive - Sensitive to cell sedimentation - Requires specific equipment - Not suitable for multi-species biofilm - Designed to study biofilms only at solid-liquid interface - Not suitable to compare multiple materials simultaneously - Materials must have electrical properties	None
Colony biofilm	Static	Solid–air	- Adhesion - Susceptibility to antimicrobial	- Easy to use - Cheap - Simple equipment - Reproducible - Large biomass in a short period	- Planktonic cells may interfere with the biofilm assays - Biofilm detachment can not be studied - Material must be customized only in the form of semipermeable membrane-like thin film - Difficulty in manipulating surfaces when colony biomass become large - Designed to study biofilms only at solid-air interface	None
Transwell	Static	Solid–air	- Adhesion - Susceptibility to antimicrobial	- Easy to use - Cheap - Simple equipment - Reproducible - High-throughput screening - Large biomass in a short period - Possibility to collect the metabolites from biofilm culture - Independent studies in compartments with different condition	- Biofilm detachment can not be studied - Material must be customized only in the form of semipermeable membrane-like thin film - Difficulty in manipulating surfaces when colony biomass become large - Designed to study biofilms only at air-solid interface	None
Robbins Device (RD)	Dynamic (variable laminar/turbulent shear)	Solid–liquid	- Adhesion - Maturation - Detachment - Susceptibility to antimicrobial	- Multiple materials tested simultaneously (up to 12) - Suitable to study early and late stage of biofilm formation - Reproducibility of real environments - Large biomass in a short period - Can operate under differenthydrodynamic conditions (e.g., laminar/turbulent) - Autoclavable and re-useable	- Not suitable for high-throughput screening - Necessity of specialized equipment - Technically challenging - Poor experimental reproducibility - Multiple microorganisms can not be tested simultaneously - Designed to study biofilms only at solid-liquid interface - No direct observation of biofilm development - Low air-liquid exchange - Flow inside the device is difficult to be accurately adjusted - Fixed geometry of the surface (disk) - Expensive - Requires specific equipment	None
Center for Disease Control (CDC) reactor	Dynamic (high laminar/turbulent shear)	Solid–liquid	- Adhesion - Maturation - Detachment - Susceptibility to antimicrobial	- Multiple materials tested simultaneously (up to 24) - Suitable to study early and late stage of biofilm formation - Reproducibility of real environments - Large biomass in a short period - Well controlled flow within the device - All surface within the device experience the share stress - Suitable for time-course studies - Can operate under differenthydrodynamic conditions (e.g., low/high and laminar/turbulent share) - Standardized biofilm method - Autoclavable and re-useable	- Not suitable for high-throughput screening - Requires specific equipment - Technically challenging - Multiple microorganisms can not be tested simultaneously - Low air-liquid exchange - Designed to study biofilms only at solid-liquid interface - No direct observation of biofilm development - Fixed geometry of the surface (disk) - Expensive - Requires a large volume of medium	- ASTM E2562-17 Standard test method for quantification of *Pseudomonas aeruginosa* biofilm grown with high shear and continuous flow using CDC biofilm reactor [105] - ASTM E2871-19 Standard test method for determining disinfectant efficacy against biofilm grown in the CDC biofilm reactor using the single tube method [106]
Rotating Disk (RD) reactor	Dynamic (intermediate laminar/turbulent shear)	Solid–liquid	- Adhesion - Maturation - Detachment - Susceptibility to antimicrobial	- Multiple materials tested simultaneously (up to 6) - Suitable to study early and late stage of biofilm formation - Reproducibility of real environments - Large biomass in a short period - Different shear can be tested simultaneously - Standardized biofilm method - Autoclavable and re-useable	- Not suitable for high-throughput screening - Requires specific equipment - Technically challenging - Multiple microorganisms can not be tested simultaneously - Low air–liquid exchange - Designed to study biofilms only at solid-liquid interface - Less suitable for time-course studies - No direct observation of biofilm development - Fixed geometry of the surface (disk) - Requires a large volume of medium - Expensive	ASTM E2196-17 Standard test method for quantification of a *Pseudomonas aeruginosa* biofilm grown with shear and continuous flow using a rotating disk reactor [107]
Annular (RA) reactor	Dynamic (variable laminar/turbulent shear)	Solid–liquid	- Adhesion - Maturation - Detachment - Susceptibility to antimicrobial	- Multiple materials tested simultaneously (up to 20) - Suitable to study early and late stage of biofilm formation - Reproducibility of real environments - Large biomass in a short period - Suitable for time-course studies - Can operate under differenthydrodynamic conditions (e.g., low/high and laminar/turbulent share) - Autoclavable and re-useable	- Not suitable for high-throughput screening - Requires specific equipment - Technically challenging - Multiple microorganisms can not be tested simultaneously - Low air-liquid exchange - Designed to study biofilms only at solid-liquid interface - No direct observation of biofilm development - Fixed geometry of the surface (rectangular) - Requires a large volume of medium - Expensive	None
Drip flow (DF) reactor	Dynamic (low laminar shear)	Solid–air	- Adhesion - Maturation - Detachment - Susceptibility to antimicrobial	- Multiple materials tested simultaneously (up to 4 or 6) - Suitable to study early and late stage of biofilm formation - Reproducibility of real environments - Large biomass in a short period - Suitable for time-course studies - Standardized biofilm method - Multiple microorganisms can be tested simultaneously - Autoclavable and re-useable - Compatible with surface of variousgeometry - High gas transfer	- Heterogeneity of biofilm developmenton the coupons - Not suitable for high-throughput screening - Requires specific equipment - Technically challenging - Designed to study biofilms only at solid-liquid interface - Direct observation of the biofilm development is not allowed - Expensive	ASTM E2647-13 Standard test method for quantification of *Pseudomonas aeruginosa* biofilm grown using drip flow biofilm reactor with low shear and continuous flow [108]
Flow chamber	Dynamic (variable laminar/turbulent shear)	Solid–liquid	- Adhesion - Maturation - Detachment - Susceptibility to antimicrobial	- Multiple materials tested simultaneously (up to 6) - Suitable to study early and late stage of biofilm formation - Reproducibility of real environments - Direct inspection of living biofilm development in a non-destructive way - Multiple microorganisms can be tested simultaneously - Autoclavable and re-useable	- Low biomass - Not suitable for high-throughput screening - Requires specific equipment - Technically challenging - Designed to study biofilms only at solid-liquid interface - Biofilm recovery during experiment is difficult - Expensive - Problem of air bubble	None
Microplate under flow (Bioflux)	Dynamic (variable laminar shear)	Solid–liquid	- Adhesion - Maturation - Detachment - Susceptibility to antimicrobial	- Easy to use - Cheap - Reproducible - Simple equipment - High-throughput screening - Multiple materials tested simultaneously (up to 96) - Multiple conditions tested simultaneously - Suitable to study early and late stage of biofilm formation - Reproducibility of real environments - Direct inspection of living biofilm development in a non-destructive way - Suitable for time-course studies - Multiple microorganisms can be tested simultaneously	- Low biomass - Designed to study biofilms only at solid-liquid interface - No biofilm recovery during experiment	
Microcosm	Static, Dynamic (variable laminar/turbulent shear)	Solid–liquid, solid–air	- Adhesion - Maturation - Detachment - Susceptibility to antimicrobial	- Hight reproducibility of real environments - Multiple materials tested simultaneously - Suitable to study early and late stage of biofilm formation - Multiple microorganisms can be tested simultaneously - Both static and dynamic systems - Compatible with surface of variousgeometry	- Less reproducibility - Not suitable for high-throughput screening - Technically challenging - Biofilm recovery during experiment is difficult - Complicated interpretation of results - Not suitable for time-course studies - No direct observation of biofilm development	None

**Table 2:** Biofilm assays suitable for the main categories of anti-biofilm surfaces. The anti-biofilm surfaces are categorized according to Cattò et al. [36].

Topic	Method	In Situ	Ex Situ	Surface Chemistry Modification	Surface Topography Modification	Surface with Microstructure	Antimicrobial Coating	Antimicrobial-Releasing Surface	Antimicrobial-Responsive Surface	Covalent Immobilization of Antimicrobials	Metal Coating	Nanoparticles Based Materials	Anti-Biofilm Biocide-Free Surfaces
Viable cellular biomass	Plate count assay	✖	✔	✔	✔	✔	✔	✔	✔	✔	✔	✔	✔
	Biomarkers quantification (Phospholipid fatty acids, ergosterol)	✖	✔	✔	✔	✔	✔	✔	✔	✔	✔	✔	✔
	Metabolic colorimetric dyes	✔	✖	✔	✔	✔	✔	✔	✔	✔	✔	✔	✔
	ATP bioluminescence	✖	✔	✔	✔	✔	✔	✔	✔	✔	✔	✔	✔
	Isothermal microcalorimetry	✔	✖	✔	✔	✔	✔	✔	✔	✔	✔	✔	✔
	Tunable Diode Laser Absorption Spectroscopy	✔	✖	✔	✔	✔	✔	✔	✔	✔	✔	✔	✔
	Propidium monoazide-qPCR	✖	✔	✔	✔	✔	✔	✔	✔	✔	✔	✔	✔
Total (viable and not viable) cellular biomass	Chamber counting	✖	✔	✔	✔	✖	✖	✖	✖	✖	✖	✔	✔
	Dyes binding	✔	✖	✔	✔	✖	✖	✖	✖	✖	✖	✔	✔
	Biomarker quantification (total organic carbon, proteins, chlorophyll)	✖	✔	✔	✔	✖	✖	✖	✖	✖	✖	✔	✔
	Quantitative polymerase chain reaction (q-PCR)	✖	✔	✔	✔	✖	✖	✖	✖	✖	✖	✔	✔
	Flow-based cell counting	✖	✔	✔	✔	✖	✖	✖	✖	✖	✖	✔	✔
Total biofilm (cellular biomass + EPS)	Dry weight	✔	✖	✔	✔	✖	✖	✖	✖	✖	✖	✔	✔
	Optical density	✖	✔	✔	✔	✖	✖	✖	✖	✖	✖	✔	✔
	Dye-based methods	✔	✖	✔	✔	✖	✖	✖	✖	✖	✖	✔	✔
	Colour measurements	✔	✔	✔	✔	✔	✔	✔	✔	✔	✔	✔	✔
EPS matrix	Proteins quantification	✖	✔	✔	✔	✔	✔	✔	✔	✔	✔	✔	✔
	Polysaccharides quantification	✖	✔	✔	✔	✔	✔	✔	✔	✔	✔	✔	✔
	Extracellular DNA quantification	✖	✔	✔	✔	✔	✔	✔	✔	✔	✔	✔	✔
	Antibody microarrays	✖	✔	✔	✔	✔	✔	✔	✔	✔	✔	✔	✔
	Fluorescent microscopies	✔	✖	✔	✔	✔	✔	✔	✔	✔	✔	✔	✔
	Spectroscopic techniques (ATR-IR, Raman, SERS)	✔	✔	✔	✔	✔	✔	✔	✔	✔	✔	✔	✔
	Advanced techniques (HPLC, LC–UV-ESI-MS/MS, GC/MS, NMR, TXRF, ICP-MS)	✖	✔	✔	✔	✔	✔	✔	✔	✔	✔	✔	✔
Identification and spatial distribution of biofilm members	FISH and FISH-based techniques	✔	✖	✔	✔	✔	✔	✔	✔	✔	✔	✔	✔
	Immunofluorescence detection	✔	✖	✔	✔	✔	✔	✔	✔	✔	✔	✔	✔
Biofilm morphology	Confocal Laser Scanning Microscopy (CLSM, FRAP, FCS, FLIM)	✔	✖	✔	✔	✔	✔	✔	✔	✔	✔	✔	✔
	Multiphoton microscopy (MPM)	✔	✖	✔	✔	✔	✔	✔	✔	✔	✔	✔	✔
	Electronic microscopies (SEM, TEM, cryo-SEM, ESEM, FIB-SEM, ASEM)	✔	✖	✔	✔	✔	✔	✔	✔	✔	✔	✔	✔
	Raman microscopy (RM, CRM)	✔	✖	✔	✔	✔	✔	✔	✔	✔	✔	✔	✔
	Scanning transmission X-ray microscopy (STXM)	✔	✖	✔	✔	✔	✔	✔	✔	✔	✔	✔	✔
	Super-resolution microscopies (STED, GSD, RESOLFT, PALM, STORM, SIM, SSIM)	✔	✖	✔	✔	✔	✔	✔	✔	✔	✔	✔	✔
	Optical coherence tomography (OCT)	✔	✖	✔	✔	✔	✔	✔	✔	✔	✔	✔	✔
Mechanical and physical properties	Atomic force microscopy (AFM, AFM-SCFS)	✔	✖	✔	✔	✔	✔	✔	✔	✔	✔	✔	✔
	Rheometry (macro and micro-reometry, SPT, MPT)	✔	✔	✔	✔	✔	✔	✔	✔	✔	✔	✔	✔
	Quartz crystal microbalance (QCM, QCM-D)	✔	✖	✔	✔	✔	✔	✔	✔	✔	✔	✔	✔
	Surface plasma resonance (SPR)	✔	✖	✔	✔	✔	✔	✔	✔	✔	✔	✔	✔
	Fluid dynamic gauging (FDG)	✔	✖	✔	✔	✔	✔	✔	✔	✔	✔	✔	✔
	Microsensors	✔	✖	✔	✔	✔	✔	✔	✔	✔	✔	✔	✔
	Nuclear magnetic resonance (NMR, MRI, PFG-NMR)	✔	✖	✔	✔	✔	✔	✔	✔	✔	✔	✔	✔

**Table 3:** Examples of the most common fluorescent stains used for staining biofilm grown on polymeric surfaces with anti-biofilm properties.

Staining Pattern	Stain	Colour (Maxima Excitation/ Emission)	Cells (Viability: Live/Dead/ Both)	Matrix	Polymeric Substrate
Nucleic acids	DAPI	Blue (358/461 nm)	X (both)		- Polyurethane coated with N-vanillylnonanamide [292] - Permanox plastic coated with B-type proanthocyanidins [293] - Surfactant polymer dressing (PluroGel) [294]
	SYTO 9	Green (485/498 nm)	X (both)		- Poly(ε-caprolactone-co-δ-valerolactone) mixed with dibromohemibasta-din-1 [81] - Silycon containing (3-acrylamidopropyl) trimethylammonium chloride (AMPTMA) or quaternized polyethylenimine methacrylate [99] - Siloxane modified with non-ionic surfactants and antioxidant [295]
	SYBR green-I	Green (495/537 nm)	X (both)		- Low density polyethylene functionalyzed with p-aminocinnamic acid and p-aminosalicylic acid [284] - Low density polyethylene functionalized with α-chymotrypsin [74]
	Live/Dead Bac-Light	Green (485/498 nm) Red (585/617 nm)	X (both)		- Nanostructured silicon [55] - Black silicon with nanoprotrusion [296] - Nanoporous 1,2-polybutadiene-b-polydimethylsiloxane loaded with sodium dodecyl sulfate [297] - Nanostructured poly (methyl methacrylate) [298] - Polydimethylsiloxane with immobilized polymyxins B and E [28] - NO-releasing amine plasma polymer [299] - Low density polyethylene functionalyzed with p-aminocinnamic acid and p-aminosalicylic acid [284] - Low density polyethylene functionalized with α-chymotrypsin [74] - Poly(methyl methacrylate) with incorporated Juglone [113] - Ciprofloxacin-incorporated polyurethane polymers [300]
	Propidium Iodide	Red (585/617 nm)	X (dead)		- Nanoporous 1,2-polybutadiene-b-polydimethylsiloxane leaded with sodium dodecyl sulfate [297] - Polymer brushes based on poly(cysteine methacrylate) grafted on on the nanocellulose membranes [301]
	Acridine orange	Green (500/526 nm)	X (both)		- Urinary catheters coated with lipase-embedded polycaprolactone (PCL), coimpregnated with the antibiotic gentamicin sulfate [302]
Plasma membrane	CellMask plasma membrane orange	Red (554/567 nm)	X (both)		- Low density polyethylene functionalyzed with p-aminocinnamic acid and p-aminosalicylic acid [76]
Cell wall	Wheat Germ Agglutinin (FITC conjugated)	Green (490/525 nm)	X (both)		- Surfactant polymer dressing (PluroGel) [294]
	Calcofluor white	Blue (355/433 nm)	X (both)		- 1H,1H,2H,2H-Perfluoro-octyl-methacrylate, methyl methacrylate, Paraloid B72 acrylic resin [303]
Metabolic activity	Resazurin	Blu (575/585 nm)	X (live)		- Poly(vinyl alcohol-co-ethylene) [101]
	Calcein or fluorescein diacetate	Green (495/515 nm)	X (live)		- Poly(vinyl alcohol-co-ethylene) [101]
	SNARF-1	Green (580/640 nm)		X	- Poly(methacrylic acid) antibiotic-loaded [304]
Proteins	FITC	Green (490/525 nm)	X (both)		- Poly (ε-caprolactone)-based polyurethane coated with butanolide [53]
	SYPRO Ruby	Red (450/610 nm)		X	- Surfactant polymer dressing (PluroGel) [294] - Polymer brushes based on poly(cysteine methacrylate) grafted on on the nanocellulose membranes [301]
Polysaccharides	Concanavalin A (Texas red, Alexa Fluor 594 conjugated)	Red (Texas red: 595/613 nm; Alexa Fluor 594: 591/618 nm;)	X (both)	X	- Low density polyethylene functionalyzed with p-aminocinnamic acid and p-aminosalicylic acid [284] - Low density polyethylene functionalized with α-chymotrypsin [74] - Silica particles attached to polydimethylsiloxane [305]

## References

[1] Namazi H. (2017). Polymers in our daily life. Bioimpacts.

[2] Teo A.J.T., Mishra A., Park I., Kim Y.J., Park W.T., Yoon Y.J. (2016). Polymeric biomaterials for medical implants and devices. ACS Biomater. Sci. Eng..

[3] Siracusa V. (2012). Food packaging permeability behaviour: A report. Int. J. Polym. Sci..

[4] Agrillo B., Balestrieri M., Gogliettino M., Palmieri G., Moretta R., Proroga Y.T., Rea I., Cornacchia A., Capuano F., Smaldone G. (2019). Functionalized polymeric materials with bio-derived antimicrobial peptides for “active” packaging. Int. J. Mol. Sci..

[5] Chen G.F.R., Zhao X.Y., Sun Y., He C.B., Tan M.C., Tan D.T.H. (2017). Low loss nanostructured polymers for chip-scale waveguide amplifiers. Sci. Rep..

[6] Cappitelli F., Zanardini E., Sorlini C. (2004). The biodeterioration of synthetic resins used in conservation. Macromol. Biosci..

[7] Giacomucci L., Toja F., Sanmartin P., Toniolo L., Prieto B., Villa F., Cappitelli F. (2012). Degradation of nitrocellulose-based paint by *Desulfovibrio desulfuricans* ATCC 13541. Biodegradation.

[8] Haque S.K.M., Ardila-Rey J.A., Umar Y., Rahman H., Mas’ud A.A., Muhammad-Sukki F., Albarracin R. (2018). Polymeric materials for conversion of electromagnetic waves from the sun to electric power. Polymers.

[9] Koniuszewska A.G., Kaczmar J.W. (2016). Application of polymer based composite materials in transportation. Prog. Rubber Plast. Recycl. Technol..

[10] Prabhu T.N., Prashantha K.A. (2018). Review on present status and future challenges of starch based polymer films and their composites in food packaging applications. Polym. Compos..

[11] Costerton J.W. (1999). Introduction to biofilm. Int. J. Antimicrob. Agents.

[12] Plyuta V.A., Lipasova V.A., Kuznetsov A.E., Khmel I.A. (2013). Effect of salicylic, indole-3-acetic, gibberellic, and abscisic acids on biofilm formation by *Agrobacterium tumefaciens* c58 and *Pseudomonas aeruginosa* PAO1. Appl. Biochem. Microbiol..

[13] Riga E.K., Vohringer M., Widyaya V.T., Lienkamp K. (2017). Polymer-based surfaces designed to reduce biofilm formation: From antimicrobial polymers to strategies for long-term applications. Macromol. Rapid Commun..

[14] Vigneron A., Alsop E.B., Chambers B., Lomans B.P., Head I.M., Tsesmetzis N. (2016). Complementary microorganisms in highly corrosive biofilms from an offshore oil production facility. Appl. Environ. Microbiol..

[15] Cappitelli F., Polo A., Villa F. (2014). Biofilm formation in food processing environments is still poorly understood and controlled. Food Eng. Rev..

[16] Douterelo I., Jackson M., Solomon C., Boxall J. (2017). Spatial and temporal analogies in microbial communities in natural drinking water biofilms. Sci. Total Environ..

[17] Galie S., Garcia-Gutierrez C., Miguelez E.M., Villar C.J., Lombo F. (2018). Biofilms in the food industry: Health aspects and control methods. Front. Microbiol..

[18] Francolini I., Donelli G., Crisante F., Taresco V., Piozzi A. (2015). Antimicrobial polymers for anti-biofilm medical devices: State-of-art and perspectives. Adv. Exp. Med. Biol..

[19] Sadekuzzaman M., Yang S., Mizan M.F.R., Ha S.D. (2015). Current and recent advanced strategies for combating biofilms. Compr. Rev. Food Sci. Food Saf..

[20] Anderson D.J., Podgorny K., Berrios-Torres S.I., Bratzler D.W., Dellinger E.P., Greene L., Nyquist A.C., Saiman L., Yokoe D.S., Maragakis L.L. (2014). Strategies to prevent surgical site infections in acute care hospitals: 2014 update. Infect. Control Hosp. Epidemiol..

[21] Badia J.M., Casey A.L., Petrosillo N., Hudson P.M., Mitchell S.A., Crosby C. (2017). Impact of surgical site infection on healthcare costs and patient outcomes: A systematic review in six European countries. J. Hosp. Infect..

[22] Lo J., Lange D., Chew B.H. (2014). Ureteral stents and foley catheters-associated urinary tract infections: The role of coatings and materials in infection prevention. Antibiotics (Basel).

[23] Johnson J.R., Kuskowski M.A., Wilt T.J. (2006). Systematic review: Antimicrobial urinary catheters to prevent catheter-associated urinary tract infection in hospitalized patients. Ann. Intern. Med..

[24] Stewart P.S. (2015). Antimicrobial tolerance in biofilms. Microbiol. Spectr..

[25] Bas S., Kramer M., Stopar D. (2017). Biofilm surface density determines biocide effectiveness. Front. Microbiol..

[26] Hoiby N., Bjarnsholt T., Givskov M., Molin S., Ciofu O. (2010). Antibiotic resistance of bacterial biofilms. Int. J. Antimicrob. Agents.

[27] Nisnevitch M. (2016). Antibiotic resistance and antibiotic alternatives: Looking towards the future. Sci. Prog..

[28] Alves D., Pereira M.O. (2016). Bio-inspired coating strategies for the immobilization of polymyxins to generate contact-killing surfaces. Macromol. Biosci..

[29] Sjollema J., Zaat S.A.J., Fontaine V., Ramstedt M., Luginbuehl R., Thevissen K., Li J.Y., van der Mei H.C., Busscher H.J. (2018). In vitro methods for the evaluation of antimicrobial surface designs. Acta Biomater..

[30] Phillips K.S., Patwardhan D., Jayan G. (2015). Biofilms, medical devices, and antibiofilm technology: Key messages from a recent public workshop. Am. J. Infect. Control.

[31] Miquel S., Lagrafeuille R., Souweine B., Forestier C. (2016). Anti-biofilm activity as a health issue. Front. Microbiol..

[32] Roy R., Tiwari M., Donelli G., Tiwari V. (2018). Strategies for combating bacterial biofilms: A focus on anti-biofilm agents and their mechanisms of action. Virulence.

[33] Yu O.Y., Zhao I.S., Mei M.L., Lo E.C., Chu C.H. (2017). Dental biofilm and laboratory microbial culture models for cariology research. Dent. J. (Basel).

[34] Haney E.F., Trimble M.J., Cheng J.T., Valle Q., Hancock R.E.W. (2018). Critical assessment of methods to quantify biofilm growth and evaluate antibiofilm activity of host defence peptides. Biomolecules.

[35] Azeredo J., Azevedo N.F., Briandet R., Cerca N., Coenye T., Costa A.R., Desvaux M., Di Bonaventura G., Hébraud M., Jaglic Z. (2017). Critical review on biofilm methods. Crit. Rev. Microbiol..

[36] Cattò C., Villa F., Cappitelli F. (2018). Recent progress in bio-inspired biofilm-resistant polymeric surfaces. Crit. Rev. Microbiol..

[37] Lichter J.A., Van Vliet K.J., Rubner M.F. (2009). Design of antibacterial surfaces and interfaces: Polyelectrolyte multilayers as a multifunctional platform. Macromolecules.

[38] Ganewatta M.S., Miller K.P., Singleton S.P., Mehrpouya-Bahrami P., Chen Y.P., Yan Y., Nagarkatti M., Nagarkatti P., Decho A.W., Tang C. (2015). Antibacterial and biofilm-disrupting coatings from resin acid-derived materials. Biomacromolecules.

[39] Antoci V., Adams C.S., Parvizi J., Davidson H.M., Composto R.J., Freeman T.A., Wickstrom E., Ducheyne P., Jungkind D., Shapiro I.M. (2008). The inhibition of *Staphylococcus epidermidis* biofilm formation by vancomycin-modified titanium alloy and implications for the treatment of periprosthetic infection. Biomaterials.

[40] Francolini I., Donelli G. (2010). Prevention and control of biofilm-based medical-device-related infections. FEMS Immunol. Med. Microbiol..

[41] Swartjes J., Sharma P.K., van Kooten T.G., van der Mei H.C., Mahmoudi M., Busscher H.J., Rochford E.T.J. (2015). Current developments in antimicrobial surface coatings for biomedical applications. Curr. Med. Chem..

[42] Ashbaugh A.G., Jiang X.S., Zheng J., Tsai A.S., Kim W.S., Thompson J.M., Miller R.J., Shahbazian J.H., Wang Y., Dillen C.A. (2016). Polymeric nanofiber coating with tunable combinatorial antibiotic delivery prevents biofilm-associated infection in vivo. Proc. Natl. Acad. Sci. USA.

[43] Barde M., Davis M., Rangari S., Mendis H.C., De La Fuente L., Auad M.L. (2018). Development of antimicrobial-loaded polyurethane films for drug-eluting catheters. J. Appl. Polym. Sci..

[44] Coenye T., Nelis H.J. (2010). In vitro and in vivo model systems to study microbial biofilm formation. J. Microbiol. Methods.

[45] Gao P., Nie X., Zou M.J., Shi Y.J., Cheng G. (2011). Recent advances in materials for extended-release antibiotic delivery system. J. Antibiot..

[46] Chen M., Yu Q.S., Sun H.M. (2013). Novel strategies for the prevention and treatment of biofilm related infections. Int. J. Mol. Sci..

[47] Zanini S., Polissi A., Maccagni E.A., Dell’Orto E.C., Liberatore C., Riccardi C. (2015). Development of antibacterial quaternary ammonium silane coatings on polyurethane catheters. J. Colloid Interface Sci..

[48] Ergene C., Palermo E.F. (2017). Cationic poly (benzyl ether) s as self-Immolative antimicrobial polymers. Biomacromolecules.

[49] Li X., Wu B., Chen H., Nan K.H., Jin Y.Y., Sun L., Wang B.L. (2018). Recent developments in smart antibacterial surfaces to inhibit biofilm formation and bacterial infections. J. Mater. Chem. B.

[50] Gbejuade H.O., Lovering A.M., Webb J.C. (2015). The role of microbial biofilms in prosthetic joint infections. Acta Orthop..

[51] Romano C.L., Scarponi S., Gallazzi E., Romano D., Drago L. (2015). Antibacterial coating of implants in orthopaedics and trauma: A classification proposal in an evolving panorama. J. Orthop. Surg. Res..

[52] Adlhart C., Verran J., Azevedo N.F., Olmez H., Keinanen-Toivola M.M., Gouveia I., Melo L.F., Crijns F. (2018). Surface modifications for antimicrobial effects in the healthcare setting: A critical overview. J. Hosp. Infect..

[53] Ding W., Ma C.F., Zhang W.P., Chiang H.Y., Tam C., Xu Y., Zhang G.Z., Qian P.Y. (2018). Anti-biofilm effect of a butenolide/polymer coating and metatranscriptomic analyses. Biofouling.

[54] Francolini I., Silvestro I., Di Lisio V., Martinelli A., Piozzi A. (2019). Synthesis, characterization, and bacterial fouling-resistance properties of polyethylene glycol-grafted polyurethane elastomers. Int. J. Mol. Sci..

[55] Hsu L.C., Fang J., Borca-Tasciuc D.A., Worobo R.W., Moraru C.I. (2013). Effect of micro- and nanoscale topography on the adhesion of bacterial cells to solid surfaces. Appl. Environ. Microbiol..

[56] Fernandez I.C.S., van der Mei H.C., Lochhead M.J., Grainger D.W., Busscher H.J. (2007). The inhibition of the adhesion of clinically isolated bacterial strains on multi-component cross-linked poly(ethylene glycol)-based polymer coatings. Biomaterials.

[57] Epstein A.K., Wong T.S., Belisle R.A., Boggs E.M., Aizenberg J. (2012). Liquid-infused structured surfaces with exceptional anti-biofouling performance. Proc. Natl. Acad. Sci. USA.

[58] Zaltsman N., Ionescu A.C., Weiss E.I., Brambilla E., Beyth S., Beyth N. (2017). Surface-modified nanoparticles as anti-biofilm filler for dental polymers. PLoS ONE.

[59] Cao W.W., Zhang Y., Wang X., Li Q., Xiao Y.H., Li P.L., Wang L.N., Ye Z.W., Xing X.D. (2018). Novel resin-based dental material with anti-biofilm activity and improved mechanical property by incorporating hydrophilic cationic copolymer functionalized nanodiamond. J. Mater. Sci. Mater. Med..

[60] Knowles B.R., Wagner P., Maclaughlin S., Higgins M.J., Molino P.J. (2017). Silica nanoparticles functionalized with zwitterionic sulfobetaine siloxane for application as a versatile antifouling coating system. ACS Appl. Mater. Interfaces.

[61] Gkana E.N., Doulgeraki A.I., Chorianopoulos N.G., Nychas G.J.E. (2017). Anti-adhesion and anti-biofilm potential of organosilane nanoparticles against foodborne pathogens. Front. Microbiol..

[62] Sufian M.M., Khattak J.Z.K., Yousaf S., Rana M.S. (2017). Safety issues associated with the use of nanoparticles in human body. Photodiagnosis Photodyn. Ther..

[63] Reed R.B., Zaikova T., Barber A., Simonich M., Lankone R., Marco M., Hristovski K., Herckes P., Passantino L., Fairbrother D.H. (2016). Potential environmental impacts and antimicrobial efficacy of silver and nanosilver-containing textiles. Environ. Sci. Technol..

[64] Resnik D.B. (2019). How should engineered nanomaterials be regulated for public and environmental health?. AMA J. Ethics.

[65] Villa F., Cappitelli F. (2013). Plant-derived bioactive compounds at sub-lethal concentrations: Towards smart biocide-free antibiofilm strategies. Phytochem. Rev..

[66] Rémy B., Mion S., Plener L., Elias M., Chabrière E., Daudé D. (2018). Interference in bacterial quorum sensing: A biopharmaceutical perspective. Front. Pharmacol..

[67] Kaplan J.B. (2010). Biofilm dispersal: Mechanisms, clinical implications, and potential therapeutic uses. J. Dent. Res..

[68] Kostakioti M., Hadjifrangiskou M., Hultgren S.J. (2013). Bacterial biofilms: Development, dispersal, and therapeutic strategies in the dawn of the postantibiotic era. Cold Spring Harb. Perspect. Med..

[69] Chifiriuc C., Grumezescu V., Grumezescu A.M., Saviuc C., Lazăr V., Andronescu E. (2012). Hybrid magnetite nanoparticles/*Rosmarinus officinalis* essential oil nanobiosystem with antibiofilm activity. Nanoscale Res. Lett..

[70] Nowatzki P.J., Koepsel R.R., Stoodley P., Min K., Harper A., Murata H., Donfack J., Hortelano E.R., Ehrlich G.D., Russell A.J. (2012). Salicylic acid-releasing polyurethane acrylate polymers as anti-biofilm urological catheter coatings. Acta Biomater..

[71] Marcano A., Ba O., Thebault P., Crétois R., Marais S., Duncan A.C. (2015). Elucidation of innovative antibiofilm materials. Colloids Surf. B.

[72] Villa F., Secundo F., Polo A., Cappitelli F. (2015). Immobilized hydrolytic enzymes exhibit antibiofilm activity against *Escherichia coli* at sub-lethal concentrations. Curr. Microbiol..

[73] Spadoni-Andreani E., Villa F., Cappitelli F., Krasowska A., Biniarz P., Lukaszewicz M., Secundo F. (2017). Coating polypropylene surfaces with protease weakens the adhesion and increases the dispersion of *Candida albicans* cells. Biotechnol. Lett..

[74] Cattò C., Secundo F., James G., Villa F., Cappitelli F. (2018). α-Chymotrypsin immobilized on a low-density polyethylene surface successfully weakens *Escherichia coli* biofilm formation. Int. J. Mol. Sci..

[75] Kim Y.G., Lee J.H., Gwon G., Kim S.I., Park J.G., Lee J. (2016). Essential oils and eugenols inhibit biofilm formation and the virulence of *Escherichia coli* O157:H7. Sci. Rep..

[76] Dell’orto S., Catto C., Villa F., Forlani F., Vassallo E., Morra M., Cappitelli F., Villa S., Gelain A. (2017). Low density polyethylene functionalized with antibiofilm compounds inhibits *Escherichia coli* cell adhesion. J. Biomed. Mater. Res. A.

[77] Sajeevan S.E., Chatterjee M., Paul V., Baranwal G., Kumar V.A., Bose C., Banerji A., Nair B.G., Prasanth B.P., Biswas R. (2018). Impregnation of catheters with anacardic acid from cashew nut shell prevents *Staphylococcus aureus* biofilm development. J. Appl. Microbiol..

[78] Pu Y., Liu A.B., Zheng Y.Q., Ye B. (2014). In vitro damage of *Candida albicans* biofilms by chitosan. Exp. Ther. Med..

[79] Bregnocchi A., Zanni E., Uccelletti D., Marra F., Cavallini D., De Angelis F., De Bellis G., Bossu M., Ierardo G., Polimeni A. (2017). Graphene-based dental adhesive with anti-biofilm activity. J. Nanobiotechnol..

[80] Namivandi-Zangeneh R., Sadrearhami Z., Bagheri A., Sauvage-Nguyen M., Ho K.K.K., Kumar N., Wong E.H.H., Boyer C. (2018). Nitric oxide-loaded antimicrobial polymer for the synergistic eradication of bacterial biofilm. ACS Macro Lett..

[81] Le Norcy T., Niemann H., Proksch P., Linossier I., Vallee-Rehel K., Hellio C., Fay F. (2017). Anti-biofilm effect of biodegradable coatings based on hemibastadin derivative in marine environment. Int. J. Mol. Sci..

[82] Liu H.Y., Shukla S., Vera-Gonzalez N., Tharmalingam N., Mylonakis E., Fuchs B.B., Shukla A. (2019). Auranofin releasing antibacterial and antibiofilm polyurethane intravascular catheter coatings. Front. Cell. Infect. Microbiol..

[83] Zhang N., Zhang K., Melo M.A.S., Weir M.D., Xu D.J., Bai Y.X., Xu H.H.K. (2017). Effects of long-term water-aging on novel anti-biofilm and protein-repellent dental composite. Int. J. Mol. Sci..

[84] Perez-Nadales E., Nogueira M.F.A., Baldin C., Castanheira S., El Ghalid M., Grund E., Lengeler K., Marchegiani E., Mehrotra P.V., Moretti M. (2014). Fungal model systems and the elucidation of pathogenicity determinants. Fungal Genet. Biol..

[85] Nett J.E., Andes D.R. (2015). Fungal Biofilms: In vivo models for discovery of anti-biofilm drugs. Microbiol. Spectr..

[86] Villa F., Pitts B., Lauchnor E., Cappitelli F., Stewart P.S. (2015). Development of a laboratory model of a phototroph-heterotroph mixed-species biofilm at the stone/air interface. Front. Microbiol..

[87] Peng C., Vishwakarma A., Li Z.R., Miyoshi T., Barton H.A., Joy A. (2018). Modification of a conventional polyurethane composition provides significant anti-biofilm activity against *Escherichia coli*. Polym. Chem..

[88] Karathia H., Vilaprinyo E., Sorribas A., Alves R. (2011). *Saccharomyces cerevisiae* as a Model Organism: A Comparative Study. PLoS ONE.

[89] Russell J.J., Theriot J.A., Sood P., Marshall W.F., Landweber L.F., Fritz-Laylin L., Polka J.K., Oliferenko S., Gerbich T., Gladfelter A. (2017). Non-model model organisms. BMC Biol..

[90] Lebeaux D., Chauhan A., Rendueles O., Beloin C. (2013). From in vitro to in vivo models of bacterial biofilm-related infections. Pathogens.

[91] Roder H.L., Sorensen S.J., Burmolle M. (2016). Studying Bacterial multispecies biofilms: Where to start?. Trends Microbiol..

[92] Rzhepishevska O., Limanska N., Galkin M., Lacoma A., Lundquist M., Sokol D., Hakobyan S., Sjostedt A., Prat C., Ramstedt M. (2018). Characterization of clinically relevant model bacterial strains of *Pseudomonas aeruginosa* for anti-biofilm testing of materials. Acta Biomater..

[93] Eydallin G., Ryall B., Maharjan R., Ferenci T. (2014). The nature of laboratory domestication changes in freshly isolated *Escherichia coli* strains. Environ. Microbiol..

[94] Elias S., Banin E. (2012). Multi-species biofilms: Living with friendly neighbors. FEMS Microbiol. Rev..

[95] Liu W.Z., Roder H.L., Madsen J.S., Bjarnsholt T., Sorensen S.J., Burmolle M. (2016). Interspecific bacterial interactions are reflected in multispecies biofilm spatial organization. Front. Microbiol..

[96] Tay W.H., Chong K.K.L., Kline K.A. (2016). Polymicrobial-host interactions during infection. J. Mol. Biol..

[97] Parijs I., Steenackers H.P. (2018). Competitive inter-species interactions underlie the increased antimicrobial tolerance in multispecies brewery biofilms. ISME J..

[98] Tan C.H., Lee K.W.K., Burmolle M., Kjelleberg S., Rice S.A. (2017). All together now: Experimental multispecies biofilm model systems. Environ. Microbiol..

[99] Zhou C., Wu Y., Thappeta K.R.V., Subramanian J.T.L., Pranantyo D., Kang E.T., Duan H.W., Kline K., Chan-Park M.B. (2017). In vivo anti-biofilm and anti-bacterial non-leachable coating thermally polymerized on cylindrical catheter. ACS Appl. Mater. Interfaces.

[100] Albuquerque M.T.P., Nagata J., Bottino M.C. (2017). Antimicrobial efficacy of triple antibiotic-eluting polymer nanofibers against multispecies biofilm. Acta Biomater..

[101] Cossu A., Si Y., Sun G., Nitin N. (2017). Antibiofilm effect of poly(vinyl alcohol-co-ethylene) halamine film against *Listeria innocua* and *Escherichia coli* O157:H7. Appl. Environ. Microbiol..

[102] Kommerein N., Stumpp S.N., Musken M., Ehlert N., Winkel A., Haussler S., Behrens P., Buettner F.F.R., Stiesch M. (2017). An oral multispecies biofilm model for high content screening applications. PLoS ONE.

[103] Roeselers G., Zippel B., Staal M., van Loosdrecht M., Muyzer G. (2006). On the reproducibility of microcosm experiments-different community composition in parallel phototrophic biofilm microcosms. FEMS Microbiol. Ecol..

[104] American Society for Testing and Materials (2017). ASTM E2799-17. Standard Test Method for Testing Disinfectant Efficacy Against Pseudomonas Aeruginosa Biofilm Using the MBEC Assay.

[105] American Society for Testing and Materials (2017). ASTM E2562-17. Standard Test Method for Quantification of Pseudomonas Aeruginosa Biofilm Grown with High Shear and Continuous Flow Using CDC Biofilm Reactor.

[106] American Society for Testing and Materials (2019). ASTM E2871-19. Standard Test Method for Determining Disinfectant Efficacy Against Biofilm Grown in the CDC Biofilm Reactor Using the Single Tube Method.

[107] American Society for Testing and Materials (2017). ASTM E2196-17. Standard Test Method for Quantification of Pseudomonas Aeruginosa Biofilm Grown with Medium Shear and Continuous Flow Using Rotating Disk Reactor.

[108] American Society for Testing and Materials (2013). ASTM E2647-13. Standard Test Method for Quantification of Pseudomonas Aeruginosa Biofilm Grown Using Drip Flow Biofilm Reactor with Low Shear and Continuous Flow.

[109] Merritt J.H., Kadouri D.E., O’Toole G.A. (2005). Growing and analyzing static biofilms. Curr. Protoc. Microbiol..

[110] O’Toole G.A. (2011). Microtiter dish biofilm formation assay. J. Vis. Exp..

[111] Lin M.H., Chang F.R., Hua M.Y., Wu Y.C., Liu S.T. (2011). Inhibitory effects of 1,2,3,4,6-penta-O-galloyl-beta-D-glucopyranose on biofilm formation by *Staphylococcus aureus*. Antimicrob. Agents Chemother..

[112] Swartjes J., Das T., Sharifi S., Subbiahdoss G., Sharma P.K., Krom B.P., Busscher H.J., van der Mei H.C. (2013). A Functional DNase I coating to prevent adhesion of bacteria and the formation of biofilm. Adv. Funct. Mater..

[113] Salta M., Dennington S.P., Wharton J.A. (2018). Biofilm inhibition by novel natural product- and biocide-containing coatings using high-throughput screening. Int. J. Mol. Sci..

[114] Ceri H., Olson M.E., Stremick C., Read R.R., Morck D., Buret A. (1999). The Calgary biofilm device: New technology for rapid determination of antibiotic susceptibilities of bacterial biofilms. J. Clin. Microbiol. Infect..

[115] Franklin M.J., Chang C., Akiyama T., Bothner B. (2015). New Technologies for studying biofilms. Microbiol. Spectr..

[116] Harrison J.J., Ceri H., Yerly J., Stremick C.A., Hu Y.P., Martinuzzi R., Turner R.J. (2006). The use of microscopy and three-dimensional visualization to evaluate the structure of microbial biofilms cultivated in the Calgary Biofilm Device. Biol. Proced. Online.

[117] Olivares E., Badel-Berchoux S., Provot C., Jaulhac B., Prevost G., Bernardi T., Jehl F. (2016). The biofilm ring test: A rapid method for routine analysis of *Pseudomonas aeruginosa* biofilm formation kinetics. J. Clin. Microbiol..

[118] Magana M., Sereti C., Ioannidis A., Mitchell C.A., Ball A.R., Magiorkinis E., Chatzipanagiotou S., Hamblin M.R., Hadjifrangiskou M., Tegos G.P. (2018). Options and limitations in clinical investigation of bacterial biofilms. Clin. Microbiol. Rev..

[119] Stadelmaier H.H. (2000). Magnetic properties of materials. Mater Sci. Eng. A Struct. Mater..

[120] Junka A.F., Janczura A., Smutnicka D., Maczynska B., Secewicz A., Nowicka J., Bartoszewicz M., Gosciniak G. (2012). Use of the real time xCelligence system for purposes of medical microbiology. Pol. J. Microbiol..

[121] Gutierrez D., Hidalgo-Cantabrana C., Rodriguez A., Garcia P., Ruas-Madiedo P. (2016). Monitoring in real time the formation and removal of biofilms from clinical related pathogens using an impedance-based technology. PLoS ONE.

[122] Ferrer M.D., Rodriguez J.C., Alvarez L., Artacho A., Royo G., Mira A. (2017). Effect of antibiotics on biofilm inhibition and induction measured by real-time cell analysis. J. Appl. Microbiol..

[123] Gutierrez D., Fernandez L., Martinez B., Ruas-Madiedo P., Garcia P., Rodriguez A. (2017). Real-Time assessment of *Staphylococcus aureus* biofilm disruption by phage-derived proteins. Front. Microbiol..

[124] Aggas J.R., Harrell W., Lutkenhaus J., Guiseppi-Elie A. (2018). Metal-polymer interface influences apparent electrical properties of nano-structured polyaniline films. Nanoscale.

[125] Wu C.C., Lin C.T., Wu C.Y., Peng W.S., Lee M.J., Tsai Y.C. (2015). Inhibitory effect of *Lactobacillus salivarius* on *Streptococcus mutans* biofilm formation. Mol. Oral Microbiol..

[126] Wang Z.L., Xiang Q.Q., Yang T., Li L.Q., Yang J.L., Li H.G., He Y., Zhang Y.H., Lu Q., Yu J.L. (2016). Autoinducer-2 of *Streptococcus mitis* as a target molecule to inhibit pathogenic multi-species biofilm formation in vitro and in an endotracheal intubation rat model. Front. Microbiol..

[127] Powell L.C., Pritchard M.F., Ferguson E.L., Powell K.A., Patel S.U., Rye P.D., Sakellakou S.M., Buurma N.J., Brilliant C.D., Copping J.M. (2018). Targeted disruption of the extracellular polymeric network of *Pseudomonas aeruginosa* biofilms by alginate oligosaccharides. Npj Biofilms Microbiomes.

[128] Kim B.Y., Thyiam G., Kang J.E., Lee S.H., Park S.H., Kim J.S., Abraham M. (2012). Development of an *Escherichia coli* biofilm model on transwell. Korean J. Clin. Lab. Sci..

[129] Standar K., Kreikemeyer B., Redanz S., Munter W.L., Laue M., Podbielski A. (2010). Setup of an in vitro test system for basic studies on biofilm behavior of mixed-species cultures with dental and periodontal pathogens. Plos ONE.

[130] Peterson S.B., Irie Y., Borlee B.R., Murakami K., Harrison J.J., Colvin K.M., Parsek M.R., Bjarnsholt T., Jensen P., Moser C., Høiby N. (2011). Different Methods for culturing biofilms in vitro. Biofilm Infections.

[131] Tran P.L., Hammond A.A., Mosley T., Cortez J., Gray T., Colmer-Hamood J.A., Shashtri M., Spallholz J.E., Hamood A.N., Reid T.W. (2009). Organoselenium coating on cellulose inhibits the formation of biofilms by *Pseudomonas aeruginosa* and *Staphylococcus aureus*. Appl. Environ. Microbiol..

[132] Bakker D.P., van der Mats A., Verkerke G.J., Busscher H.J., van der Mei H.C. (2003). Comparison of velocity profiles for different flow chamber designs used in studies of microbial adhesion to surfaces. Appl. Environ. Microbiol..

[133] Goeres D.M., Loetterle L.R., Hamilton M.A., Murga R., Kirby D.W., Donlan R.M. (2005). Statistical assessment of a laboratory method for growing biofilms. Microbiology.

[134] Gomes I.B., Meireles A., Goncalves A.L., Goeres D.M., Sjollema J., Simoes L.C., Simoes M. (2018). Standardized reactors for the study of medical biofilms: A review of the principles and latest modifications. Crit. Rev. Biotechnol..

[135] Schwartz K., Stephenson R., Hernandez M., Jambang N., Boles B.R. (2010). The use of drip flow and rotating disk reactors for *Staphylococcus aureus* biofilm analysis. J. Vis. Exp..

[136] Sebestyen P., Blanken W., Bacsa I., Toth G., Martin A., Bhaiji T., Dergez A., Kesseru P., Koos A., Kiss I. (2016). Upscale of a laboratory rotating disk biofilm reactor and evaluation of its performance over a half-year operation period in outdoor conditions. Algal Res..

[137] Linton C.J., Sherriff A., Millar M.R. (1999). Use of a modified Robbins device to directly compare the adhesion of *Staphylococcus epidermidis* RP62A to surfaces. J. Appl. Microbiol..

[138] McCoy W.F., Bryers J.D., Robbins J., Costerton J.W. (1981). Observations of fouling biofilm formation. Can. J. Microbiol..

[139] Oosterhof J.J.H., Buijssen K., Busscher H.J., van der Laan B., van der Mei H.C. (2006). Effects of quaternary ammonium silane coatings on mixed fungal and bacterial biofilms on tracheoesophageal shunt prostheses. Appl. Environ. Microbiol..

[140] Ramage G., Wickes B.L., Lopez-Ribot J.L. (2008). A seed and feed model for the formation of Candida albicans biofilms under flow conditions using an improved modified Robbins device. Rev. Iberoam. Micol..

[141] Ginige M.P., Garbin S., Wylie J., Krishna K.C.B. (2017). Effectiveness of devices to monitor biofouling and metals deposition on plumbing materials exposed to a full-scale drinking water distribution system. PLoS ONE.

[142] Cai W.Y., Wu J.F., Xi C.W., Meyerhoff M.E. (2012). Diazeniumdiolate-doped poly(lactic-co-glycolic acid)-based nitric oxide releasing films as antibiofilm coatings. Biomaterials.

[143] Li Y., Carrera C., Chen R., Li J., Lenton P., Rudney J.D., Jones R.S., Aparicio C., Fok A. (2014). Degradation in the dentin-composite interface subjected to multi-species biofilm challenges. Acta Biomater..

[144] Pitts B., Willse A., McFeters G.A., Hamilton M.A., Zelver N., Stewart P.S. (2001). A repeatable laboratory method for testing the efficacy of biocides against toilet bowl biofilms. J. Appl. Microbiol..

[145] Cotter J.J., O’Gara J.P., Stewart P.S., Pitts B., Casey E. (2010). Characterization of a modified rotating disk reactor for the cultivation of *Staphylococcus epidermidis* biofilm. J. Appl. Microbiol..

[146] Barry D.M., McGrath P.B. (2016). Rotation disk process to assess the influence of metals and voltage on the growth of biofilm. Materials.

[147] Gomes I.B., Simoes M., Simoes L.C. (2014). An overview on the reactors to study drinking water biofilms. Water Res..

[148] Pintar K.D.M., Slawson R.M. (2003). Effect of temperature and disinfection strategies on ammonia-oxidizing bacteria in a bench-scale drinking water distribution system. Water Res..

[149] Ndiongue S., Huck P.M., Slawson R.M. (2005). Effects of temperature and biodegradable organic matter on control of biofilms by free chlorine in a model drinking water distribution system. Water Res..

[150] Jang H.J., Choi Y.J., Ka J.O. (2011). Effects of diverse water pipe materials on bacterial communities and water quality in the annular reactor. J. Microbiol. Biotechnol..

[151] Goeres D.M., Hamilton M.A., Beck N.A., Buckingham-Meyer K., Hilyard J.D., Loetterle L.R., Lorenz L.A., Walker D.K., Stewart P.S. (2009). A method for growing a biofilm under low shear at the air-liquid interface using the drip flow biofilm reactor. Nat. Protoc..

[152] Sawant S.N., Selvaraj V., Prabhawathi V., Doble M. (2013). Antibiofilm properties of silver and gold incorporated PU, PCLm, PC and PMMA nanocomposites under two shear conditions. PLoS ONE.

[153] Goodwin D.G., Xia Z., Gordon T.B., Gao C., Bouwer E.J., Fairbrother D.H. (2016). Biofilm development on carbon nanotube/polymer nanocomposites. Environ. Sci. Nano.

[154] Salli K.M., Ouwehand A.C. (2015). The use of in vitro model systems to study dental biofilms associated with caries: A short review. J. Oral Microbiol..

[155] Jaramillo D.E., Arriola A., Safavi K., de Paz L.E.C. (2012). Decreased bacterial adherence and biofilm growth on surfaces coated with a solution of benzalkonium chloride. J. Endod..

[156] Francolini I., Norris P., Piozzi A., Donelli G., Stoodley P. (2004). Usnic acid, a natural antimicrobial agent able to inhibit bacterial biofilm formation on polymer surfaces. Antimicrob. Agents Chemother..

[157] Fabbri S., Dennington S.P., Price C., Stoodley P., Longyear J. (2018). A marine biofilm flow cell for in situ screening marine fouling control coatings using optical coherence tomography. Ocean. Eng..

[158] Tremblay Y.D.N., Vogeleer P., Jacques M., Harel J. (2015). High-throughput microfluidic method to study biofilm formation and host-pathogen interactions in pathogenic *Escherichia coli*. Appl. Environ. Microbiol..

[159] Benoit M.R., Conant C.G., Ionescu-Zanetti C., Schwartz M., Matin A. (2010). New device for high-throughput viability screening of flow biofilms. Appl. Environ. Microbiol..

[160] Moormeier D.E., Endres J.L., Mann E.E., Sadykov M.R., Horswill A.R., Rice K.C., Fey P.D., Bayles K.W. (2013). Use of microfluidic technology to analyze gene expression during *Staphylococcus aureus* biofilm formation reveals distinct physiological niches. Appl. Environ. Microbiol..

[161] Abdulkareem E.H., Memarzadeh K., Allaker R.P., Huang J., Pratten J., Spratt D. (2015). Anti-biofilm activity of zinc oxide and hydroxyapatite nanoparticles as dental implant coating materials. J. Dent..

[162] Li F., Weir M.D., Fouad A.F., Xu H.H.K. (2014). Effect of salivary pellicle on antibacterial activity of novel antibacterial dental adhesives using a dental plaque microcosm biofilm model. Dent. Mater..

[163] Wood T.K., Knabel S.J., Kwan B.W. (2013). Bacterial persister cell formation and dormancy. Appl. Environ. Microbiol..

[164] Hooijmans C.M., Abdin T.A., Alaerts G.J. (1995). Quantification of viable biomass in biofilm reactors by extractable lipid phosphate. Appl. Microbiol. Biotechnol..

[165] Quideau S.A., McIntosh A.C.S., Norris C.E., Lloret E., Swallow M.J.B., Hannam K. (2016). Extraction and analysis of microbial phospholipid fatty acids in soils. J. Vis. Exp..

[166] Willers C., van Rensburg P.J.J., Claassens S. (2015). Phospholipid fatty acid profiling of microbial communities-a review of interpretations and recent applications. J. Appl. Microbiol..

[167] Gehron M.J., White D.C. (1983). Sensitive assay of phospholipid glycerol in environmental sample. J. Microbiol. Met..

[168] Oursel D., Loutelier-Bourhis C., Orange N., Chevalier S., Norris V., Lange C.M. (2007). Identification and relative quantification of fatty acids in *Escherichia coli* membranes by gas chromatography/mass spectrometry. Rapid Commun. Mass Spectrom..

[169] Li L., Han J.J., Wang Z.P., Liu J.A., Wei J.C., Xiong S.X., Zhao Z.W. (2014). Mass spectrometry methodology in lipid analysis. Int. J. Mol. Sci..

[170] Jain S., Caforio A., Driessen A.J.M. (2014). Biosynthesis of archaeal membrane ether lipids. Front. Microbiol..

[171] Gors S., Schumann R., Haubner N., Karsten U. (2007). Fungal and algal biomass in biofilms on artificial surfaces quantified by ergosterol and chlorophyll a as biomarkers. Int. Biodeterior. Biodegrad..

[172] Hippelein M., Rugamer M. (2004). Ergosterol as an indicator of mould growth on building materials. Int. J. Hyg. Environ. Health.

[173] Ng H.E., Raj S.S.A., Wong S.H., Tey D., Tan H.M. (2008). Estimation of fungal growth using the ergosterol assay: A rapid tool in assessing the microbiological status of grains and feeds. Lett. Appl. Microbiol..

[174] Pantanella F., Valenti P., Natalizi T., Passeri D., Berlutti F. (2013). Analytical techniques to study microbial biofilm on abiotic surfaces: Pros and cons of the main techniques currently in use. Ann. Ig..

[175] Welch K., Cai Y., Strømme M. (2012). A method for quantitative determination of biofilm viability. J. Funct. Biomater..

[176] Trafny E.A., Lewandowski R., Zawistowska-Marciniak I., Stepinska M. (2013). Use of MTT assay for determination of the biofilm formation capacity of microorganisms in metalworking fluids. World J. Microbiol. Biotechnol..

[177] Nante N., Ceriale E., Messina G., Lenzi D., Manzi P. (2017). Effectiveness of ATP bioluminescence to assess hospital cleaning: A review. J. Prev. Med. Hyg..

[178] AlLuhaybi K.A.R., Alghaith G.Y., Moneib N.A., Yassien M.A.M. (2015). Generation of recombinant bioluminescent *Escherichia coli* for quantitative determination of bacterial adhesion. Pak. J. Pharm. Sci..

[179] Ivanova E.P., Alexeeva Y.V., Pham D.K., Wright J.P., Nicolau D.V. (2006). ATP level variations in heterotrophic bacteria during attachment on hydrophilic and hydrophobic surfaces. Int. Microbiol..

[180] Braissant O., Wirz D., Gopfert B., Daniels A.U. (2010). Use of isothermal microcalorimetry to monitor microbial activities. FEMS Microbiol. Lett..

[181] Solokhina A., Bruckner D., Bonkat G., Braissant O. (2017). Metabolic activity of mature biofilms of *Mycobacterium tuberculosis* and other non-tuberculous mycobacteria. Sci. Rep..

[182] Said J., Walker M., Parsons D., Stapleton P., Beezer A.E., Gaisford S. (2015). Development of a flow system for studying biofilm formation on medical devices with microcalorimetry. Methods.

[183] Brueckner D., Roesti D., Zuber U.G., Schmidt R., Kraehenbuehl S., Bonkat G., Braissant O. (2016). Comparison of tunable diode laser absorption spectroscopy and isothermal micro-calorimetry for non-invasive detection of microbial growth in media fills. Sci. Rep..

[184] Wilson C., Lukowicz R., Merchant S., Valquier-Flynn H., Caballero J., Sandoval J., Okuom M., Huber C., Brooks T.D., Wilson E. (2017). Quantitative and qualitative assessment methods for biofilm growth: A mini-review. Res. Rev. J. Eng. Technol..

[185] Bogachev M.I., Volkov V.Y., Markelov O.A., Trizna E.Y., Baydamshina D.R., Melnikov V., Murtazina R.R., Zelenikhin P.V., Sharafutdinov I.S., Kayumov A.R. (2018). Fast and simple tool for the quantification of biofilm-embedded cells sub-populations from fluorescent microscopic images. PLoS ONE.

[186] Shi L., Gunther S., Hubschmann T., Wick L.Y., Harms H., Muller S. (2007). Limits of propidium iodide as a cell viability indicator for environmental bacteria. Cytom. A.

[187] Netuschil L., Auschill T.M., Sculean A., Arweiler N.B. (2014). Confusion over live/dead stainings for the detection of vital microorganisms in oral biofilms—Which stain is suitable?. BMC Oral Health.

[188] Stiefel P., Schmidt-Emrich S., Maniura-Weber K., Ren Q. (2015). Critical aspects of using bacterial cell viability assays with the fluorophores SYTO9 and propidium iodide. BMC Microbiol..

[189] Dobor J., Varga M., Zaray G. (2012). Biofilm controlled sorption of selected acidic drugs on river sediments characterized by different organic carbon content. Chemosphere.

[190] Bradford M.M. (1976). A rapid and sensitive method for the quantitation of microgram quantities of protein utilizing the principle of protein-dye binding. Anal. Biochem..

[191] Lowry O.H., Rosebrough N.J., Farr A.L., Randall R.J. (1951). Protein measurement with the folin phenol reagent. J. Biol. Chem..

[192] Smith P.K., Krohn R.I., Hermanson G.T., Mallia A.K., Gartner F.H., Provenzano M.D., Fujimoto E.K., Goeke N.M., Olson B.J., Klenk D.C. (1985). Measurement of protein using bicinchoninic acid. Anal. Biochem..

[193] Jesus B., Perkins R.G., Mendes C.R., Brotas V., Paterson D.M. (2006). Chlorophyll fluorescence as a proxy for microphytobenthic biomass: Alternatives to the current methodology. Mar. Biol..

[194] Sanmartin P., Aira N., Devesa-Rey R., Silva B., Prieto B. (2010). Relationship between color and pigment production in two stone biofilm-forming cyanobacteria (*Nostoc* sp PCC 9104 and *Nostoc* sp PCC 9025). Biofouling.

[195] Sendersky E., Simkovsky R., Golden S.S., Schwarz R. (2017). Quantification of chlorophyll as a proxy for biofilm formation in the cyanobacterium *Synechococcus Elongatus*. Bio-Protoc..

[196] Chamizo S., Adessi A., Mugnai G., Simiani A., De Philippis R. (2018). Soil Type and Cyanobacteria species influence the macromolecular and chemical characteristics of the polysaccharidic matrix in induced biocrusts. Microb. Ecol..

[197] Fernandez-Silva I., Sanmartin P., Silva B., Moldes A., Prieto B. (2011). Quantification of phototrophic biomass on rocks: Optimization of chlorophyll-a extraction by response surface methodology. J. Ind. Microbiol. Biotechnol..

[198] Vazquez-Nion D., Silva B., Prieto B. (2018). Bioreceptivity index for granitic rocks used as construction material. Sci. Total Environ..

[199] Vazquez-Nion D., Silva B., Prieto B. (2018). Influence of the properties of granitic rocks on their bioreceptivity to subaerial phototrophic biofilms. Sci. Total Environ..

[200] Dalwai F., Spratt D.A., Pratten J. (2007). Use of quantitative PCR and culture methods to characterize ecological flux in bacterial biofilms. J. Clin. Microbiol..

[201] Alvarez G., Gonzalez M., Isabal S., Blanc V., Leon R. (2013). Method to quantify live and dead cells in multi-species oral biofilm by real-time PCR with propidium monoazide. Amb. Express.

[202] Taylor M.J., Bentham R.H., Ross K.E. (2014). Limitations of using propidium monoazide with qpcr to discriminate between live and dead *Legionella* in biofilm samples. Microbiol. Insights.

[203] Soto-Munoz L., Teixido N., Usall J., Vinas I., Crespo-Sempere A., Torres R. (2014). Development of PMA real-time PCR method to quantify viable cells of Pantoea agglomerans CPA-2, an antagonist to control the major postharvest diseases on oranges. Int. J. Food Microbiol..

[204] Ambriz-Aviña V., Contreras-Garduño J.A., Pedraza-Reyes M. (2014). Applications of flow cytometry to characterize bacterial physiological responses. Biomed. Res. Int..

[205] Kerstens M., Boulet G., Van Kerckhoven M., Clais S., Lanckacker E., Delputte P., Maes L., Cos P. (2015). A flow cytometric approach to quantify biofilms. Folia Microbiol..

[206] Bakke R., Kommedal R., Kalvenes S. (2001). Quantification of biofilm accumulation by an optical approach. J. Microbiol. Methods.

[207] Stiefel P., Rosenberg U., Schneider J., Mauerhofer S., Maniura-Weber K., Ren Q. (2016). Is biofilm removal properly assessed? Comparison of different quantification methods in a 96-well plate system. Appl. Microbiol. Biotechnol..

[208] O’Toole G.A., Kolter R. (1998). Initiation of biofilm formation in *Pseudomonas fluorescens* WCS365 proceeds via multiple, convergent signalling pathways: A genetic analysis. Mol. Microbiol..

[209] Young M.E., Wakefield R., Urquhart D.C.M., Nicholson K., Tonge K. (1995). Assesment in a field setting of the efficacy of various biocides on sandstone. Int. Coll Methods of Evaluating Products for the Conservation of Porous Building Materials in Monuments.

[210] Prieto B., Rivas T., Silva B. (2002). Rapid quantification of phototrophic microorganisms and their physiological state through their colour. Biofouling.

[211] Vazquez-Nion D., Sanmartin P., Silva B., Prieto B. (2013). Reliability of color measurements for monitoring pigment content in a biofilm-forming cyanobacterium. Int. Biodeterior. Biodegrad..

[212] Sanmartin P., Villa F., Polo A., Silva B., Prieto B., Cappitelli F. (2015). Rapid evaluation of three biocide treatments against the cyanobacterium *Nostoc* sp PCC 9104 by color changes. Ann. Microbiol..

[213] Sanmartin P., Vazquez-Nion D., Arines J., Cabo-Dominguez L., Prieto B. (2017). Controlling growth and colour of phototrophs by using simple and inexpensive coloured lighting: A preliminary study in the Light4Heritage project towards future strategies for outdoor illumination. Int. Biodeterior. Biodegrad..

[214] Prieto B., Vazquez-Nion D., Silva B., Sanmartin P. (2018). Shaping colour changes in a biofilm-forming cyanobacterium by modifying the culture conditions. Algal Res..

[215] Fairchild M.D. (2018). International Commission on Illumination. CIE 015:2018 Colorimetry.

[216] Sanmartin P., Villa F., Silva B., Cappitelli F., Prieto B. (2011). Color measurements as a reliable method for estimating chlorophyll degradation to phaeopigments. Biodegradation.

[217] Di Martino P. (2018). Extracellular polymeric substances, a key element in understanding biofilm phenotype. Aims Microbiol..

[218] Blanco Y., Rivas L.A., González-Toril E., Ruiz-Bermejo M., Moreno-Paz M., Parro V., Palacín A., Aguilera Á., Puente-Sánchez F. (2019). Environmental parameters, and not phylogeny, determine the composition of extracellular polymeric substances in microbial mats from extreme environments. Sci. Total Environ..

[219] Jachlewski S., Jachlewski W.D., Linne U., Bräsen C., Wingender J., Siebers B. (2015). Isolation of extracellular polymeric substances from biofilms of the thermoacidophilic archaeon *Sulfolobus acidocaldarius*. Front. Bioeng. Biotechnol..

[220] McSwain B.S., Irvine R.L., Hausner M., Wilderer P.A. (2005). Composition and distribution of extracellular polymeric substances in aerobic flocs and granular sludge. Appl. Environ. Microbiol..

[221] Cho J., Hermanowicz S.W., Hur J. (2012). Effects of experimental conditions on extraction yield of extracellular polymeric substances by cation exchange resin. Sci. World J..

[222] Rossi F., Mugnai G., De Philippis R. (2018). Complex role of the polymeric matrix in biological soil crusts. Plant Soil.

[223] Pan X.L., Liu J., Zhang D.Y., Chen X., Li L.H., Song W.J., Yang J.Y. (2010). A comparison of five extraction methods for extracellular polymeric substances (EPS) from biofilm by using three-dimensional excitation-emission matrix (3DEEM) fluorescence spectroscopy. Water Sa.

[224] Liu H., Fang H.H.P. (2002). Extraction of extracellular polymeric substances (EPS) of sludges. J. Biotechnol..

[225] Villa F., Remelli W., Forlani F., Gambino M., Landini P., Cappitelli F. (2012). Effects of chronic sub-lethal oxidative stress on biofilm formation by *Azotobacter vinelandii*. Biofouling.

[226] Cattò C., Grazioso G., Dell’Orto S., Gelain A., Villa S., Marzano V., Vitali A., Villa F., Cappitelli F., Forlani F. (2017). The response of *Escherichia coli* biofilm to salicylic acid. Biofouling.

[227] Masuko T., Minami A., Iwasaki N., Majima T., Nishimura S.I., Lee Y.C. (2005). Carbohydrate analysis by a phenol-sulfuric acid method in microplate format. Anal. Biochem..

[228] Nielsen S.S. (2010). Total carbohydrate by phenol-sulfuric acid method. Food Analysis Laboratory Manual.

[229] Ruhmann B., Schmid J., Sieber V. (2015). Methods to identify the unexplored diversity of microbial exopolysaccharides. Front. Microbiol..

[230] Mojica K., Elsey D., Cooney M.J. (2007). Quantitative analysis of biofilm EPS uronic acid content. J. Microbiol. Methods.

[231] Khodse V.B., Bhosle N.B. (2010). Differences in carbohydrate profiles in batch culture grown planktonic and biofilm cells of *Amphora rostrata* Wm. Sm.. Biofouling.

[232] Tielen P., Rosenau F., Wilhelm S., Jaeger K.E., Flemming H.C., Wingender J. (2010). Extracellular enzymes affect biofilm formation of mucoid *Pseudomonas Aeruginosa*. Microbiology.

[233] Blumenkr N., Asboehan G. (1973). New method for quantitative-determination of uronic acids. Anal. Biochem..

[234] van den Hoogen B.M., van Weeren P.R., Lopes-Cardozo M., van Golde L.M.G., Barneveld A., van de Lest C.H.A. (1998). A microtiter plate assay for the determination of uronic acids. Anal. Biochem..

[235] Wu J.F., Xi C.W. (2009). Evaluation of different methods for extracting extracellular DNA from the biofilm matrix. Appl. Environ. Microbiol..

[236] Das T., Sehar S., Manefield M. (2013). The roles of extracellular DNA in the structural integrity of extracellular polymeric substance and bacterial biofilm development. Environ. Microbiol. Rep..

[237] Steinberger R.E., Holden P.A. (2005). Extracellular DNA in single- and multiple-species unsaturated biofilms. Appl. Environ. Microbiol..

[238] Tang L., Schramm A., Neu T.R., Revsbech N.P., Meyer R.L. (2013). Extracellular DNA in adhesion and biofilm formation of four environmental isolates: A quantitative study. FEMS Microbiol. Ecol..

[239] Jiao Y.Q., Cody G.D., Harding A.K., Wilmes P., Schrenk M., Wheeler K.E., Banfield J.F., Thelen M.P. (2010). Characterization of extracellular polymeric substances from acidophilic microbial biofilms. Appl. Environ. Microbiol..

[240] Nan L., Yang K., Ren G.G. (2015). Anti-biofilm formation of a novel stainless steel against *Staphylococcus aureus*. Mat. Sci. Eng. C.

[241] Kelestemur S., Avci E., Culha M. (2018). Raman and surface-enhanced raman scattering for biofilm characterization. Chemosensors.

[242] Ramirez-Mora T., Davila-Perez C., Torres-Mendez F., Valle-Bourrouet G. (2019). Raman spectroscopic characterization of endodontic biofilm matrices. J. Spectrosc..

[243] Chao Y.Q., Zhang T. (2012). Surface-enhanced Raman scattering (SERS) revealing chemical variation during biofilm formation: From initial attachment to mature biofilm. Anal. Bioanal. Chem..

[244] Xu H.C., He P.J., Wang G.Z., Shao L.M. (2010). Three-dimensional excitation emission matrix fluorescence spectroscopy and gel-permeating chromatography to characterize extracellular polymeric substances in aerobic granulation. Water Sci. Technol..

[245] Bales P.M., Renke E.M., May S.L., Shen Y., Nelson D.C. (2013). Purification and characterization of biofilm-associated eps exopolysaccharides from ESKAPE organisms and other pathogens. PLoS ONE.

[246] Ruhmann B., Schmid J., Sieber V. (2014). Fast carbohydrate analysis via liquid chromatography coupled with ultra violet and electrospray ionization ion trap detection in 96-well format. J. Chromatogr. A.

[247] Ramirez-Mora T., Retana-Lobo C., Valle-Bourrouet G. (2018). Biochemical characterization of extracellular polymeric substances from endodontic biofilms. PLoS ONE.

[248] Hasan N., Gopal J., Wu H.F. (2011). Rapid, sensitive and direct analysis of exopolysaccharides from biofilm on aluminum surfaces exposed to sea water using MALDI-TOF MS. J. Mass Spectrom..

[249] Gonzalez-Gil G., Thomas L., Emwas A.H., Lens P.N., Saikaly P.E. (2015). NMR and MALDI-TOF MS based characterization of exopolysaccharides in anaerobic microbial aggregates from full-scale reactors. Sci. Rep..

[250] Yildiz F., Fong J., Sadovskaya I., Grard T., Vinogradov E. (2014). Structural characterization of the extracellular polysaccharide from *Vibrio cholerae* O1 El-Tor. PLoS ONE.

[251] Neu T.R., Lawrence J.R. (2014). Advanced techniques for in situ analysis of the biofilm matrix (structure, composition, dynamics) by means of laser scanning microscopy. Methods Mol. Biol..

[252] Cowan S.E., Gilbert E., Liepmann D., Keasling J.D. (2000). Commensal interactions in a dual-species biofilm exposed to mixed organic compounds. Appl. Environ. Microbiol..

[253] Chen M.Y., Lee D.J., Tay J.H., Show K.Y. (2007). Staining of extracellular polymeric substances and cells in bioaggregates. Appl. Microbiol. Biotechnol..

[254] Berk V., Fong J.C.N., Dempsey G.T., Develioglu O.N., Zhuang X.W., Liphardt J., Yildiz F.H., Chu S. (2012). Molecular architecture and assembly principles of *Vibrio cholerae* biofilms. Science.

[255] Sportelli M.C., Tutuncu E., Picca R.A., Valentini M., Valentini A., Kranz C., Mizaikoff B., Barth H., Cioffi N. (2017). Inhibiting, *P. Fluorescens* biofilms with fluoropolymer-embedded silver nanoparticles: An in-situ spectroscopic study. Sci. Rep..

[256] Feng J., de la Fuente-Núñez C., Trimble M.J., Xu J., Hancock R.E., Lu X. (2015). An in situ Raman spectroscopy-based microfluidic “lab-on-a-chip” platform for non-destructive and continuous characterization of Pseudomonas aeruginosa biofilms. Chem. Commun..

[257] Greuter D., Loy A., Horne M., Ratteil T. (2016). probeBase-an online resource for rRNA-targeted oligonucleotide probes and primers: New features 2016. Nucleic Acids Res..

[258] Schimak M.P., Kleiner M., Wetzel S., Liebeke M., Dubilier N., Fuchs B.M. (2016). MiL-FISH: Multilabeled oligonucleotides for fluorescence in situ hybridization improve visualization of bacterial cells. Appl. Environ. Microbiol..

[259] Almeida C., Azevedo N.F., Santos S., Keevil C.W., Vieira M.J. (2011). Discriminating multi-species populations in biofilms with peptide nucleic acid fluorescence in situ hybridization (PNA FISH). PLoS ONE.

[260] Azevedo A.S., Almeida C., Pereira B., Madureira P., Wengel J., Azevedo N.F. (2015). Detection and discrimination of biofilm populations using locked nucleic acid/2 ‘-O-methyl-RNA fluorescence in situ hybridization (LNA/2 ‘ OMe-FISH). Biochem. Eng. J..

[261] Kubota K. (2013). CARD-FISH for environmental microorganisms: Technical advancement and future applications. Microbes Environ..

[262] Escudero C., Vera M., Oggerin M., Amils R. (2018). Active microbial biofilms in deep poor porous continental subsurface rocks. Sci. Rep..

[263] Stoecker K., Dorninger C., Daims H., Wagner M. (2010). Double labeling of oligonucleotide probes for fluorescence in situ hybridization (DOPE-FISH) improves signal intensity and increases rRNA accessibility. Appl. Environ. Microbiol..

[264] Valm A.M., Oldenbourg R., Borisy G.G. (2016). Multiplexed spectral imaging of 120 different fluorescent labels. PLoS ONE.

[265] Valm A.M., Welch J.L.M., Borisy G.G. (2012). CLASI-FISH: Principles of combinatorial labeling and spectral imaging. Syst. Appl. Microbiol..

[266] Congestri R. (2008). FISH methods in phycology: Phototrophic biofilm and phytoplankton applications. Plant Biosyst..

[267] Musat N., Foster R., Vagner T., Adam B., Kuypers M.M.M. (2012). Detecting metabolic activities in single cells, with emphasis on nanoSIMS. FEMS Microbiol. Rev..

[268] Li T., Wu T.D., Mazeas L., Toffin L., Guerquin-Kern J.L., Leblon G., Bouchez T. (2008). Simultaneous analysis of microbial identity and function using NanoSIMS. Environ. Microbiol..

[269] Kniggendorf A.K., Nogueira R., Kelb C., Schadzek P., Meinhardt-Wollweber M., Ngezahayo A., Roth B. (2016). Confocal Raman microscopy and fluorescent in situ hybridization-a complementary approach for biofilm analysis. Chemosphere.

[270] Manti A., Boi P., Amalfitano S., Puddu A., Papa S. (2011). Experimental improvements in combining CARD-FISH and flow cytometry for bacterial cell quantification. J. Microbiol. Methods.

[271] Janse J.D., Kokoskova B. (2009). Indirect immunofluorescence microscopy for the detection and identification of plant pathogenic bacteria (in particular for *Ralstonla solanacearum*). Methods Mol. Biol..

[272] Bruneval P., Choucair J., Paraf F., Casalta J.P., Raoult D., Scherchen F., Mainardi J.L. (2001). Detection of fastidious bacteria in cardiac valves in cases of blood culture negative endocarditis. J. Clin. Pathol..

[273] Lin M., Todoric D., Mallory M., Luo B.S., Trottier E., Dan H.H. (2006). Monoclonal antibodies binding to the cell surface of *Listeria monocytogenes* serotype 4b. J. Med. Microbiol..

[274] Foulston L., Elsholz A.K.W., DeFrancesco A.S., Losick R. (2014). The extracellular matrix of *Staphylococcus aureus* biofilms comprises cytoplasmic proteins that associate with the cell surface in response to decreasing pH. MBio.

[275] Vejborg R.M., Klemm P. (2009). Cellular chain formation in *Escherichia coli* biofilms. Microbiology.

[276] Luo T.L., Eisenberg M.C., Hayashi M.A.L., Gonzalez-Cabezas C., Foxman B., Marrs C.F., Rickard A.H. (2018). A Sensitive thresholding method for confocal laser scanning microscope image stacks of microbial biofilms. Sci. Rep..

[277] Heydorn A., Nielsen A.T., Hentzer M., Sternberg C., Givskov M., Ersboll B.K., Molin S. (2000). Quantification of biofilm structures by the novel computer program COMSTAT. Microbiology.

[278] Mueller L.N., de Brouwer J.F., Almeida J.S., Stal L.J., Xavier J.B. (2006). Analysis of a marine phototrophic biofilm by confocal laser scanning microscopy using the new image quantification software PHLIP. BMC Ecol..

[279] Sommerfeld Ross S.S., Tu M.H., Falsetta M.L., Ketterer M.R., Kiedrowski M.R., Horswill A.R., Apicella M.A., Reinhardt J.M., Fiegel J. (2014). Quantification of confocal images of biofilms grown on irregular surfaces. J. Microbiol. Methods.

[280] Miura K. (2016). Bioimage Data Analysis.

[281] Neu T.R., Manz B., Volke F., Dynes J.J., Hitchcock A.P., Lawrence J.R. (2010). Advanced imaging techniques for assessment of structure, composition and function in biofilm systems. FEMS Microbiol. Ecol..

[282] Oubekka S.D., Briandet R., Fontaine-Aupart M.P., Steenkeste K. (2012). Correlative time-resolved fluorescence microscopy to assess antibiotic diffusion-reaction in biofilms. Antimicrob. Agents Chemother..

[283] Davison W.M., Pitts B., Stewart P.S. (2010). Spatial and temporal patterns of biocide action against *Staphylococcus epidermidis* biofilms. Antimicrob. Agents Chemother..

[284] Cattò C., James G., Villa F., Villa S., Cappitelli F. (2018). Zosteric acid and salicylic acid bound to a low density polyethylene surface successfully control bacterial biofilm formation. Biofouling.

[285] Schneider J.P., Basler M. (2016). Shedding light on biology of bacterial cells. Philos. Trans. R. Soc. Lond. B Biol. Sci..

[286] Power R.M., Huisken J. (2017). A guide to light-sheet fluorescence microscopy for multiscale imaging. Nat. Methods.

[287] Parthasarathy R. (2018). Monitoring microbial communities using light sheet fluorescence microscopy. Curr. Opin. Microbiol..

[288] Janissen R., Murillo D.M., Niza B., Sahoo P.K., Nobrega M.M., Cesar C.L., Temperini M.L.A., Carvalho H.F., de Souza A.A., Cotta M.A. (2015). Spatiotemporal distribution of different extracellular polymeric substances and filamentation mediate Xylella fastidiosa adhesion and biofilm formation. Sci. Rep..

[289] Yan J., Sharo A.G., Stone H.A., Wingreen N.S., Bassler B.L. (2016). *Vibrio cholerae* biofilm growth program and architecture revealed by single-cell live imaging. Proc. Natl. Acad. Sci. USA.

[290] Bryers J.D. (2001). Two-photon excitation microscopy for analyses of biofilm processes. Methods Enzymol..

[291] Thomsen H., Graf F.E., Farewell A., Ericson M.B. (2018). Exploring photoinactivation of microbial biofilms using laser scanning microscopy and confined 2-photon excitation. J. Biophotonics.

[292] Villa F., Giacomucci L., Polo A., Principi P., Toniolo L., Levi M., Turri S., Cappitelli F. (2009). N-vanillylnonanamide tested as a non-toxic antifoulant, applied to surfaces in a polyurethane coating. Biotechnol. Lett..

[293] Trentin D.S., Silva D.B., Frasson A.P., Rzhepishevska O., da Silva M.V., Pulcini E.E.L., James G., Soares G.V., Tasca T., Ramstedt M. (2015). Natural Green coating inhibits adhesion of clinically important bacteria. Sci. Rep..

[294] Das Ghatak P., Mathew-Steiner S.S., Pandey P., Roy S., Sen C.K. (2018). A surfactant polymer dressing potentiates antimicrobial efficacy in biofilm disruption. Sci. Rep..

[295] Akuzov D., Franca L., Grunwald I., Vladkova T. (2018). Sharply reduced biofilm formation from cobetia marina and in black sea water on modified siloxane coatings. Coatings.

[296] Ivanova E.P., Hasan J., Webb H.K., Gervinskas G., Juodkazis S., Truong V.K., Wu A.H.F., Lamb R.N., Baulin V.A., Watson G.S. (2013). Bactericidal activity of black silicon. Nat. Commun..

[297] Li L., Molin S., Yang L., Ndoni S. (2013). Sodium dodecyl sulfate (SDS)-loaded nanoporous polymer as anti-biofilm surface coating material. Int. J. Mol. Sci..

[298] Dickson M.N., Liang E.I., Rodriguez L.A., Vollereaux N., Yee A.F. (2015). Nanopatterned polymer surfaces with bactericidal properties. Biointerphases.

[299] Ho K.K.K., Ozcelik B., Willcox M.D.P., Thissen H., Kumar N. (2017). Facile solvent-free fabrication of nitric oxide (NO)-releasing coatings for prevention of biofilm formation. Chem. Commun..

[300] Xu Y.C., Wang J.Z., Hao Z.Y., Wang S., Liang C.Z. (2019). Biodegradable ciprofloxacin-incorporated waterborne polyurethane polymers prevent bacterial biofilm formation in vitro. Exp. Ther. Med..

[301] Valencia L., Kumar S., Jalvo B., Mautner A., Salazar-Alvarez G., Mathew A.P. (2018). Fully bio-based zwitterionic membranes with superior antifouling and antibacterial properties prepared via surface-initiated free-radical polymerization of poly(cysteine methacrylate). J. Mater. Chem. A.

[302] Dave R.N., Joshi H.M., Venugopalan V.P. (2011). Novel biocatalytic polymer-based antimicrobial coatings as potential ureteral biomaterial: Preparation and in vitro performance evaluation. Antimicrob. Agents Chemother..

[303] Sabatini V., Cattò C., Cappelletti G., Cappitelli F., Antenucci S., Farina H., Ortenzi M.A., Camazzola S., Di Silvestro G. (2018). Protective features, durability and biodegration study of acrylic and methacrylic fluorinated polymer coatings for marble protection. Prog. Org. Coat..

[304] Albright V., Zhuk I., Wang Y.H., Selin V., van de Belt-Gritter B., Busscher H.J., van der Mei H.C., Sukhishvili S.A. (2017). Self-defensive antibiotic-loaded layer-by-layer coatings: Imaging of localized bacterial acidification and pH-triggering of antibiotic release. Acta Biomater..

[305] Lagree K., Mon H.H., Mitchell A.P., Ducker W.A. (2018). Impact of surface topography on biofilm formation by *Candida albicans*. PLoS ONE.

[306] Alhede M., Qvortrup K., Liebrechts R., Hoiby N., Givskov M., Bjarnsholt T. (2012). Combination of microscopic techniques reveals a comprehensive visual impression of biofilm structure and composition. FEMS Immunol. Med. Microbiol..

[307] Sugimoto S., Okuda K., Miyakawa R., Sato M., Arita-Morioka K., Chiba A., Yamanaka K., Ogura T., Mizunoe Y., Sato C. (2016). Imaging of bacterial multicellular behaviour in biofilms in liquid by atmospheric scanning electron microscopy. Sci. Rep..

[308] Bridier A., Meylheuc T., Briandet R. (2013). Realistic representation of Bacillus subtilis biofilms architecture using combined microscopy (CLSM, ESEM and FESEM). Micron.

[309] Asahi Y., Miura J., Tsuda T., Kuwabata S., Tsunashima K., Noiri Y., Sakata T., Ebisu S., Hayashi M. (2015). Simple observation of *Streptococcus mutans* biofilm by scanning electron microscopy using ionic liquids. AMB Express.

[310] Gonzalez-Ramirez A.I., Ramirez-Granillo A., Medina-Canales M.G., Rodriguez-Tovar A.V., Martinez-Rivera M.A. (2016). Analysis and description of the stages of Aspergillus fumigatus biofilm formation using scanning electron microscopy. BMC Microbiol..

[311] Gomes L.C., Mergulhao F.J. (2017). SEM analysis of surface impact on biofilm antibiotic treatment. Scanning.

[312] Mohmmed S.A., Vianna M.E., Penny M.R., Hilton S.T., Mordan N., Knowles J.C. (2017). Confocal laser scanning, scanning electron, and transmission electron microscopy investigation of *Enterococcus faecalis* biofilm degradation using passive and active sodium hypochlorite irrigation within a simulated root canal model. Microbiologyopen.

[313] McCutcheon J., Southam G. (2018). Advanced biofilm staining techniques for TEM and SEM in geomicrobiology: Implications for visualizing EPS architecture, mineral nucleation, and microfossil generation. Chem. Geol..

[314] Hrubanova K., Nebesarova J., Ruzicka F., Krzyzanek V. (2018). The innovation of cryo-SEM freeze-fracturing methodology demonstrated on high pressure frozen biofilm. Micron.

[315] Liu M.H., Wu X.X., Li J.K., Liu L., Zhang R.G., Shao D.Y., Du X.D. (2017). The specific anti-biofilm effect of gallic acid on *Staphylococcus aureus* by regulating the expression of the ica operon. Food Control.

[316] Nishiyama H., Koizumi M., Ogawa K., Kitamura S., Konyuba Y., Watanabe Y., Ohbayashi N., Fukuda M., Suga M., Sato C. (2014). Atmospheric scanning electron microscope system with an open sample chamber: Configuration and applications. Ultramicroscopy.

[317] Sahl S.J., Hell S.W., Jakobs S. (2017). Fluorescence nanoscopy in cell biology. Nat. Rev. Mol. Cell. Biol..

[318] Blom H., Widengren J. (2017). Stimulated emission depletion microscopy. Chem. Rev..

[319] Coltharp C., Xiao J. (2012). Superresolution microscopy for microbiology. Cell Microbiol..

[320] Xiao J., Dufrêne Y.F. (2016). Optical and force nanoscopy in microbiology. Nat. Microbiol..

[321] Li C.K., Kuang C.F., Liu X. (2018). Prospects for fluorescence nanoscopy. ACS Nano.

[322] Sydor A.M., Czymmek K.J., Puchner E.M., Mennella V. (2015). Super-resolution microscopy: From single molecules to supramolecular assemblies. Trends Cell. Biol..

[323] Ball G., Demmerle J., Kaufmann R., Davis I., Dobbie I.M., Schermelleh L. (2015). SIMcheck: A toolbox for successful super-resolution structured illumination microscopy. Sci. Rep..

[324] Virdis B., Harnisch F., Batstone D.J., Rabaey K., Donose B.C. (2012). Non-invasive characterization of electrochemically active microbial biofilms using confocal Raman microscopy. Energy Environ. Sci..

[325] Wagner M., Horn H. (2017). Optical coherence tomography in biofilm research: A comprehensive review. Biotechnol. Bioeng..

[326] Sandt C., Smith-Palmer T., Comeau J., Pink D. (2009). Quantification of water and biomass in small colony variant PAO1 biofilms by confocal Raman microspectroscopy. Appl. Microbiol. Biotechnol..

[327] Sandt C., Smith-Palmer T., Pink J., Brennan L., Pink D. (2007). Confocal Raman microspectroscopy as a tool for studying the chemical heterogeneities of biofilms in situ. J. Appl. Microbiol..

[328] Wagner M., Ivleva N.P., Haisch C., Niessner R., Horn H. (2009). Combined use of confocal laser scanning microscopy (CLSM) and Raman microscopy (RM): Investigations on EPS-Matrix. Water Res..

[329] Andrews J.S., Rolfe S.A., Huang W.E., Scholes J.D., Banwart S.A. (2010). Biofilm formation in environmental bacteria is influenced by different macromolecules depending on genus and species. Environ. Microbiol..

[330] Lawrence J.R., Swerhone G.D.W., Dynes J.J., Hitchcock A.P., Korber D.R. (2016). Complex organic corona formation on carbon nanotubes reduces microbial toxicity by suppressing reactive oxygen species production. Environ. Sci. Nano.

[331] Yang S.I., George G.N., Lawrence J.R., Kaminskyj S.G.W., Dynes J.J., Lai B., Pickering I.J. (2016). Multispecies biofilms transform selenium oxyanions into elemental selenium particles: Studies using combined synchrotron x-ray fluorescence imaging and scanning transmission x-ray microscopy. Environ. Sci. Technol..

[332] Blauert F., Horn H., Wagner M. (2015). Time-resolved biofilm deformation measurements using optical coherence tomography. Biotechnol. Bioeng..

[333] Heidari A.E., Moghaddam S., Truong K.K., Chou L., Genberg C., Brenner M., Chena Z.P. (2016). Visualizing biofilm formation in endotracheal tubes using endoscopic three-dimensional optical coherence tomography. J. Biomed. Opt..

[334] Farid M.U., Guo J.X., An A.K. (2018). Bacterial inactivation and in situ monitoring of biofilm development on graphene oxide membrane using optical coherence tomography. J. Memb. Sci..

[335] Dreszer C., Wexler A.D., Drusova S., Overdijk T., Zwijnenburg A., Flemming H.C., Kruithof J.C., Vrouwenvelder J.S. (2014). In-situ biofilm characterization in membrane systems using optical coherence tomography: Formation, structure, detachment and impact of flux change. Water Res..

[336] Fortunato L., Jeong S., Leiknes T. (2017). Time-resolved monitoring of biofouling development on a flat sheet membrane using optical coherence tomography. Sci. Rep..

[337] Ogrodzki P., Cheung C.S., Saad M., Dahmani K., Coxill R., Liang H.D., Forsythe S.J. (2017). Rapid in situ imaging and whole genome sequencing of biofilm in neonatal feeding tubes: A clinical proof of concept. Sci. Rep..

[338] de Andrade M.C.L., de Oliveira M.A.S., dos Santos F.D.G., Vilela P.D.X., da Silva M.N., Macedo D.P.C., Neto R.G.D., Neves H.J.P., Brandao I.D.L., Chaves G.M. (2017). A new approach by optical coherence tomography for elucidating biofilm formation by emergent Candida species. PLoS ONE.

[339] Rupp C.J., Fux C.A., Stoodley P. (2005). Viscoelasticity of *Staphylococcus aureus* biofilms in response to fluid shear allows resistance to detachment and facilitates rolling migration. Appl. Environ. Microbiol..

[340] Kim M.K., Drescher K., Pak O.S., Bassler B.L., Stone H.A. (2014). Filaments in curved streamlines: Rapid formation of *Staphylococcus aureus* biofilm streamers. New J. Phys..

[341] Billings N., Birjiniuk A., Samad T.S., Doyle P.S., Ribbeck K. (2015). Material properties of biofilms—A review of methods for understanding permeability and mechanics. Rep. Progr. Phys..

[342] Boudarel H., Mathias J.D., Blaysat B., Grediac M. (2018). Towards standardized mechanical characterization of microbial biofilms: Analysis and critical review. NPJ Biofilms Microbiomes.

[343] Bol M., Ehret A.E., Albero A.B., Hellriegel J., Krull R. (2013). Recent advances in mechanical characterisation of biofilm and their significance for material modelling. Crit. Rev. Biotechnol..

[344] Martin K.J., Bolster D., Derlon N., Morgenroth E., Nerenberg R. (2014). Effect of fouling layer spatial distribution on permeate flux: A theoretical and experimental study. J. Membr. Sci..

[345] Li C.Y., Wagner M., Lackner S., Horn H. (2016). Assessing the influence of biofilm surface roughness on mass transfer by combining optical coherence tomography and two-dimensional modeling. Biotechnol. Bioeng..

[346] Jafari M., Desmond P., van Loosdrecht M.C.M., Derlon N., Morgenroth E., Picioreanu C. (2018). Effect of biofilm structural deformation on hydraulic resistance during ultrafiltration: A numerical and experimental study. Water Res..

[347] Picioreanu C., Blauert F., Horn H., Wagner M. (2018). Determination of mechanical properties of biofilms by modelling the deformation measured using optical coherence tomography. Water Res..

[348] Dufrene Y.F. (2015). Sticky microbes: Forces in microbial cell adhesion. Trends Microbiol..

[349] James S.A., Powell L.C., Wright C.J., Dhanasekaran D., Thajuddin N. (2016). Atomic force microscopy of biofilms-imaging, interactions, and mechanics. Microbial Biofilms—Importance and Applications.

[350] Lau P.C., Dutcher J.R., Beveridge T.J., Lam J.S. (2009). Absolute quantitation of bacterial biofilm adhesion and viscoelasticity by microbead force spectroscopy. Biophys. J..

[351] Harapanahalli A.K., Chen Y., Li J., Busscher H.J., van der Mei H.C. (2015). Influence of adhesion force on icaA and cidA Gene expression and production of matrix components in *Staphylococcus aureus* biofilms. Appl. Environ. Microbiol..

[352] Feuillie C., Formosa-Dague C., Hays L.M., Vervaeck O., Derclaye S., Brennan M.P., Foster T.J., Geoghegan J.A., Dufrêne Y.F. (2017). Molecular interactions and inhibition of the staphylococcal biofilm-forming protein SdrC. Proc. Natl. Acad. Sci. USA.

[353] El-Kirat-Chatel S., Puymege A., Duong T.H., Van Overtvelt P., Bressy C., Belec L., Dufrêne Y.F., Molmeret M. (2017). Phenotypic heterogeneity in attachment of marine bacteria toward antifouling copolymers unraveled by AFM. Front. Microbiol..

[354] Kundukad B., Seviour T., Liang Y., Rice S.A., Kjelleberg S., Doyle P.S. (2016). Mechanical properties of the superficial biofilm layer determine the architecture of biofilms. Soft Matter.

[355] Taubenberger A.V., Hutmacher D.W., Muller D.J. (2014). Single-cell force spectroscopy, an emerging tool to quantify cell adhesion to biomaterials. Tissue Eng. Part B Rev..

[356] Spengler C., Thewes N., Jung P., Bischoff M., Jacobs K. (2017). Determination of the nano-scaled contact area of staphylococcal cells. Nanoscale.

[357] Klapper I., Rupp C.J., Cargo R., Purvedorj B., Stoodley P. (2002). Viscoelastic fluid description of bacterial biofilm material properties. Biotechnol. Bioeng..

[358] Peterson B.W., He Y., Ren Y., Zerdoum A., Libera M.R., Sharma P.K., van Winkelhoff A.J., Neut D., Stoodley P., van der Mei H.C. (2015). Viscoelasticity of biofilms and their recalcitrance to mechanical and chemical challenges. FEMS Microbiol. Rev..

[359] Stojkovic B., Sretenovic S., Dogsa I., Poberaj I., Stopar D. (2015). Viscoelastic properties of levan-DNA mixtures important in microbial biofilm formation as determined by micro-and macrorheology. Biophys. J..

[360] Kesel S., Grumbein S., Gumperlein I., Tallawi M., Marel A.K., Lieleg O., Opitz M. (2016). Direct comparison of physical properties of *Bacillus subtilis* NCIB 3610 and B-1 Biofilms. Appl. Environ. Microbiol..

[361] Di Stefano A., D’Aurizio E., Trubiani O., Grande R., Di Campli E., Di Giulio M., Di Bartolomeo S., Sozio P., Iannitelli A., Nostro A. (2009). Viscoelastic properties of *Staphylococcus aureus* and *Staphylococcus epidermidis* mono-microbial biofilms. Microb. Biotechnol..

[362] Pavlovsky L., Younger J.G., Solomon M.J. (2013). In situ rheology of *Staphylococcus epidermidis* bacterial biofilms. Soft Matter.

[363] Grumbein S., Werb M., Opitz M., Lieleg O. (2016). Elongational rheology of bacterial biofilms in situ. J. Rheol..

[364] Galy O., Latour-Lambert P., Zrelli K., Ghigo J.M., Beloin C., Henry N. (2012). Mapping of bacterial biofilm local mechanics by magnetic microparticle actuation. Biophys. J..

[365] Cao H.Y., Habimana O., Safari A., Heffernan R., Dai Y.H., Casey E. (2016). Revealing region-specific biofilm viscoelastic properties by means of a micro-rheological approach. NPJ Biofilms Microbiomes.

[366] Chew S.C., Kundukad B., Seviour T., van der Maarel J.R.C., Yang L., Rice S.A., Doyle P., Kjelleberg S. (2014). Dynamic remodeling of microbial biofilms by functionally distinct exopolysaccharides. Mbio.

[367] Olofsson A.C., Hermansson M., Elwing H. (2005). Use of a quartz crystal microbalance to investigate the antiadhesive potential of N-acetyl-L-cysteine. Appl. Environ. Microbiol..

[368] Chen J.Y., Penn L.S., Xi J. (2018). Quartz crystal microbalance: Sensing cell-substrate adhesion and beyond. Biosens. Bioelectron..

[369] Dixon M.C. (2008). Quartz crystal microbalance with dissipation monitoring: Enabling real-time characterization of biological materials and their interactions. J. Biomol. Tech..

[370] Costa F., Sousa D.M., Parreira P., Lamghari M., Gomes P., Martins M.C.L. (2017). N-acetylcysteine-functionalized coating avoids bacterial adhesion and biofilm formation. Sci. Rep..

[371] Tonda-Turo C., Carmagnola I., Ciardelli G. (2018). Quartz crystal microbalance with dissipation monitoring: A powerful method to predict the in vivo behavior of bioengineered surfaces. Front. Bioeng. Biotechnol..

[372] Reipa V., Almeida J., Cole K.D. (2006). Long-term monitoring of biofilm growth and disinfection using a quartz crystal microbalance and reflectance measurements. J. Microbiol. Methods.

[373] Sprung C., Wahlisch D., Huttl R., Seidel J., Meyer A., Wolf G. (2009). Detection and monitoring of biofilm formation in water treatment systems by quartz crystal microbalance sensors. Water Sci. Technol..

[374] Wang Y.N., Narain R., Liu Y. (2014). Study of bacterial adhesion on different glycopolymer surfaces by quartz crystal microbalance with dissipation. Langmuir.

[375] Knowles B.R., Yang D., Wagner P., Maclaughlin S., Higgins M.J., Molino P.J. (2019). Zwitterion functionalized silica nanoparticle coatings: The effect of particle size on protein, bacteria, and fungal spore adhesion. Langmuir.

[376] Tam K., Kinsinger N., Ayala P., Qi F., Shi W., Myung N.V. (2007). Real-time monitoring of *Streptococcus mutans* biofilm formation using a quartz crystal microbalance. Caries Res..

[377] Olsson A.L., van der Mei H.C., Busscher H.J., Sharma P.K. (2009). Influence of cell surface appendages on the bacterium-substratum interface measured real-time using QCM-D. Langmuir.

[378] Pranzetti A., Salaun S., Mieszkin S., Callow M.E., Callow J.A., Preece J.A., Mendes P.M. (2012). Model organic surfaces to probe marine bacterial adhesion kinetics by surface plasmon resonance. Adv. Funct. Mater..

[379] Zhang P., Guo J.S., Yan P., Chen Y.P., Wang W., Dai Y.Z., Fang F., Wang G.X., Shen Y. (2018). Dynamic dispersal of surface layer biofilm induced by nanosized tio2 based on surface plasmon resonance and waveguide. Appl. Environ. Microbiol..

[380] Gordon P.W., Brooker A.D.M., Chew Y.M.J., Wilson D.I., York D.W. (2010). A scanning fluid dynamic gauging technique for probing surface layers. Meas. Sci. Technol..

[381] Peck O.P.W., Chew Y.M.J., Bird M.R., Bolhuis A. (2015). Application of fluid dynamic gauging in the characterization and removal of biofouling deposits. Heat Transf. Eng..

[382] Beyenal H., Babauta J. (2014). Microsensors and microscale gradients in biofilms. Adv. Biochem. Eng. Biotechnol..

[383] Lee J.H., Seo Y., Lim T.S., Bishop P.L., Papautsky I. (2007). MEMS needle-type sensor array for in situ measurements of dissolved oxygen and redox potential. Environ. Sci. Technol..

[384] Moya A., Guimera X., del Campo F.J., Prats-Alfonso E., Dorado A.D., Baeza M., Villa R., Gabriel D., Gamisans X., Gabriel G. (2014). Biofilm oxygen profiling using an array of microelectrodes on a micro fabricated needle. Procedia Eng..

[385] Masi E., Ciszak M., Santopolo L., Frascella A., Giovannetti L., Marchi E., Viti C., Mancuso S. (2015). Electrical spiking in bacterial biofilms. J. R. Soc. Interface.

[386] James G.A., Zhao A.G., Usui M., Underwood R.A., Nguyen H., Beyenal H., Pulcini E.D., Hunt A.A., Bernstein H.C., Fleckman P. (2016). Microsensor and transcriptomic signatures of oxygen depletion in biofilms associated with chronic wounds. Wound Repair Regen..

[387] Ito T., Okabe S., Satoh H., Watanabe Y. (2002). Successional development of sulfate-reducing bacterial populations and their activities in a wastewater biofilm growing under microaerophilic conditions. Appl. Environ. Microbiol..

[388] Hibiya K., Terada A., Tsuneda S., Hirata A. (2003). Simultaneous nitrification and denitrification by controlling vertical and horizontal microenvironment in a membrane-aerated biofilm reactor. J. Biotechnol..

[389] Lee W.H., Wahman D.G., Pressman J.G. (2013). Amperometric carbon fiber nitrite microsensor for in situ biofilm monitoring. Sens. Actuators B Chem..

[390] von der Schulenburg D.A.G., Pintelon T.R.R., Picioreanu C., Van Loosdrecht M.C.M., Johns M.L. (2009). Three-dimensional simulations of biofilm growth in porous media. AIChE J..

[391] Bottero S., Storck T., Heimovaara T.J., van Loosdrecht M.C.M., Enzien M.V., Picioreanu C. (2013). Biofilm development and the dynamics of preferential flow paths in porous media. Biofouling.

[392] Davit Y., Byrne H., Osborne J., Pitt-Francis J., Gavaghan D., Quintard M. (2013). Hydrodynamic dispersion within porous biofilms. Phys. Rev..

[393] Qin C.Z., Hassanizadeh S.M. (2015). Pore-network modeling of solute transport and biofilm growth in porous media. Transp. Porous Med..

[394] McLean J.S., Ona O.N., Majors P.D. (2008). Correlated biofilm imaging, transport and metabolism measurements via combined nuclear magnetic resonance and confocal microscopy. ISME J..

[395] Phoenix V.R., Holmes W.M. (2008). Magnetic resonance imaging of structure, diffusivity, and copper immobilization in a phototrophic biofilm. Appl. Environ. Microbiol..

[396] Vogt M., Flemming H.C., Veeman W.S. (2000). Diffusion in *Pseudomonas aeruginosa* biofilms: A pulsed field gradient NMR study. J. Biotechnol..

[397] Gabrilska R.A., Rumbaugh K.P. (2015). Biofilm models of polymicrobial infection. Future Microbiol..

[398] Brann M., Suter J.D., Addleman R.S., Larimer C. (2017). Monitoring bacterial biofilms with a microfluidic flow chip designed for imaging with white-light interferometry. Biomicrofluidics.

[399] Lourenco A., Coenye T., Goeres D.M., Donelli G., Azevedo A.S., Ceri H., Coelho F.L., Flemming H.C., Juhna T., Lopes S.P. (2014). Minimum information about a biofilm experiment ( MIABiE): Standards for reporting experiments and data on sessile microbial communities living at interfaces. Pathog. Dis..

[400] Lourenco A., Ferreira A., Veiga N., Machado I., Pereira M.O., Azevedo N.F. (2012). BiofOmics: A Web platform for the systematic and standardized collection of high-throughput biofilm data. PLoS ONE.

[401] Coenye T., Goeres D., Van Bambeke F., Bjarnsholt T. (2018). Should standardized susceptibility testing for microbial biofilms be introduced in clinical practice?. Clin. Microbiol. Infect..

